# Palaeohistology reveals a slow pace of life for the dwarfed Sicilian elephant

**DOI:** 10.1038/s41598-021-02192-4

**Published:** 2021-11-24

**Authors:** Meike Köhler, Victoria Herridge, Carmen Nacarino-Meneses, Josep Fortuny, Blanca Moncunill-Solé, Antonietta Rosso, Rossana Sanfilippo, Maria Rita Palombo, Salvador Moyà-Solà

**Affiliations:** 1 Institut Català de Paleontologia Miquel Crusafont, Universitat Autònoma de Barcelona, Cerdanyola del Vallès, Barcelona, Spain; 2 Institució Catalana de Recerca i Estudis Avançats (ICREA), Barcelona, Spain; 3 Earth Sciences, Natural History Museum, Cromwell Road, London, UK; 4 Department of Biological Sciences, University of Cape Town, Rondebosch, Cape Town, South Africa; 5 Dipartimento di Scienze, Università degli Studi Roma Tre, Roma, Italy; 6 Centro de Investigacións Científicas Avanzadas, Universidade da Coruña, A Coruña, Spain; 7 Dipartimento di Scienze Biologiche, Geologiche e Ambientali, Università di Catania, Catania, Italy; 8 c7o Earth Science Department, IGAG-CNR, Sapienza University of Rome, Rome, Italy

**Keywords:** Evolution, Palaeontology

## Abstract

The 1-m-tall dwarf elephant *Palaeoloxodon falconeri* from the Pleistocene of Sicily (Italy) is an extreme example of insular dwarfism and epitomizes the Island Rule. Based on scaling of life-history (LH) traits with body mass, *P. falconeri* is widely considered to be ‘*r*-selected’ by truncation of the growth period, associated with an early onset of reproduction and an abbreviated lifespan. These conjectures are, however, at odds with predictions from LH models for adaptive shifts in body size on islands. To settle the LH strategy of *P. falconeri*, we used bone, molar, and tusk histology to infer growth rates, age at first reproduction, and longevity. Our results from all approaches are congruent and provide evidence that the insular dwarf elephant grew at very slow rates over an extended period; attained maturity at the age of 15 years; and had a minimum lifespan of 68 years. This surpasses not only the values predicted from body mass but even those of both its giant sister taxon (*P. antiquus*) and its large mainland cousin (*L. africana*). The suite of LH traits of *P. falconeri* is consistent with the LH data hitherto inferred for other dwarfed insular mammals. *P. falconeri*, thus, not only epitomizes the Island Rule but it can also be viewed as a paradigm of evolutionary change towards a slow LH that accompanies the process of dwarfing in insular mammals.

Dwarfing, an adaptive evolutionary process^1^ by which the descendant grows to a smaller adult size than its ancestor, may evolve as a byproduct of selection acting primarily on LH characteristics, particularly on age at sexual maturity^2^ (ASM). LH theory posits that a small adult size can result from either advancement or postponement of sexual maturity, depending on the ecological scenario^3–5^. Broadly speaking, high extrinsic mortality (predation, parasite loads) curtails the *time-period of growth* (truncation) by forcing an early channeling of resources from growth to reproduction^6^, which results in growth arrest and an advanced onset of sexual maturity; the organism is small-at-maturity. Low resource availability, by contrast, forces a reduction in the *rate of growth*^2,7^, generally associated with prolonged allocation of resources to maintenance^6,7^ and a correlated delay in sexual maturity^2,4,8^; the organism remains small-for-age throughout ontogeny. Rate and duration of growth, hence, are the two variables (at least in determinate growers) that mediate the pace of life in response to prevailing environmental conditions.

Dwarfing is a ubiquitous phenomenon that is particularly pervasive on islands where it affects large mammals and dinosaurs and, to a lesser extent, other vertebrates and even plants^9–13^. Insular dwarfing is now widely considered to be an adaptive response to selection pressures imposed by environmental conditions where net primary production and, hence, per capita food resources are low, and/or under elevated population density^2,14–22^, but see^23–26^. Paradoxically, despite the widely accepted notion that insular environments are characterized by low resource availability, the concept of growth truncation as the developmental process behind insular dwarfing is pervasive throughout literature^23–28^, even when growth rates (GRs) are slow^27^.

Among herbivores, elephants have the slowest relative GRs^29^. Elephants form a group of very large mammals characterized by a slow LH with a slow GR, delayed age at maturity and long lifespan; indeed, they commonly live longer than 60 years in the wild^30^. Along with humans, they represent the slow end of the slow-fast LH continuum among terrestrial mammals, which is usually depicted as a mouse—elephant continuum. Through the scaling with body mass, their slow life-history is a direct result of their gigantic size. Unsurprisingly, hence, it is intuitively assumed that ‘dwarfed giants’ shifted towards a faster LH as in the case of *P. falconeri*^23^ from the late Middle Pleistocene of Sicily (Spinagallo cave, Syracuse, Supplementary material 1). At just 0.9 to 1.2 m tall, and with an estimated mean body mass of 252 kg (Supplementary material 2; Supplementary Tables 1, 2, 3), *P. falconeri* is the smallest elephant to have ever evolved; it weighted little more than 2% of its ancestor *P. antiquus* (11,500 kg^31^). Raia and colleagues^23^ calculated discrete LH values from interspecific scaling. Accordingly, the dwarf elephant attained sexual maturity at the age of 3–4 years, pregnancy took 189 days, and life span was 26 years. They considered a fast LH to be supported by an elevated number of unfused long bones interpreted as high calf mortality, and by the high number of tuskless females that supposedly arrested tusk growth to divert resources to reproduction. Because of their higher mass estimation for *P. falconeri*, Larramendi and Palombo^25^ provide similar but somewhat higher values. Roth^21^ suggested dwarfing in *P. falconeri* to be a consequence of selection for reduced energy use (through scaling of metabolic rate with body mass) and calculated a shortened growth period of 4 years based on body mass scaling. She furthermore speculated that by retention of a relatively long gestation period at a smaller body size, twins might have been more frequent than singletons. In current literature, *P. falconeri* is still being used as an example of evolution towards the fast end of the slow-fast LH continuum associated with insular dwarfing^28^.

In this study, we reconstruct the key LH traits ‘age at maturity’ and ‘longevity’, as well as the rate and duration of growth in *P. falconeri* from a histological analysis of bones (skeletochronology; beginning of the external fundamental system EFS; accretional bone area), molars (daily enamel secretion rate ESR; enamel extension rate EER; plate formation time PFT; crown formation time CFT), and tusks (dentine daily secretion rate DSR; dentine extension rate DER; first FOI, second SOI, and third TOI order increments), using multiple technical (microscopy, 3D imaging) and statistical (von Bertalanffy; segmented regression) tools. We perform phylogenetic generalized least square regressions (PGLS) to evaluate potential phylogenetic signals in allometric life-history/body mass analyses of *P. falconeri*, extant ungulates and elephants. We rely on external measurements and 178 histological slides from 29 tibiae, 1 upper fourth deciduous tooth, 1 lower third molar, and 6 tusks. We compare the resulting LH data with those from the extant, full-sized continental cousin *Loxodonta africana* and, as far as available, with PFT of fossil continental *Mammuthus columbi*^32^ *P. antiquus* (own data), and insular *P. cypriotes*^32^, to establish whether *P. falconeri* dwarfed via growth truncation or GR reduction (see “Materials and methods”). In doing so, we aim to bring the first comprehensive data on insular dwarf elephant LHs to bear on the debate over causality between the process of dwarfing and the evolution of LH strategies on islands.

## Results

**Figure 1:**
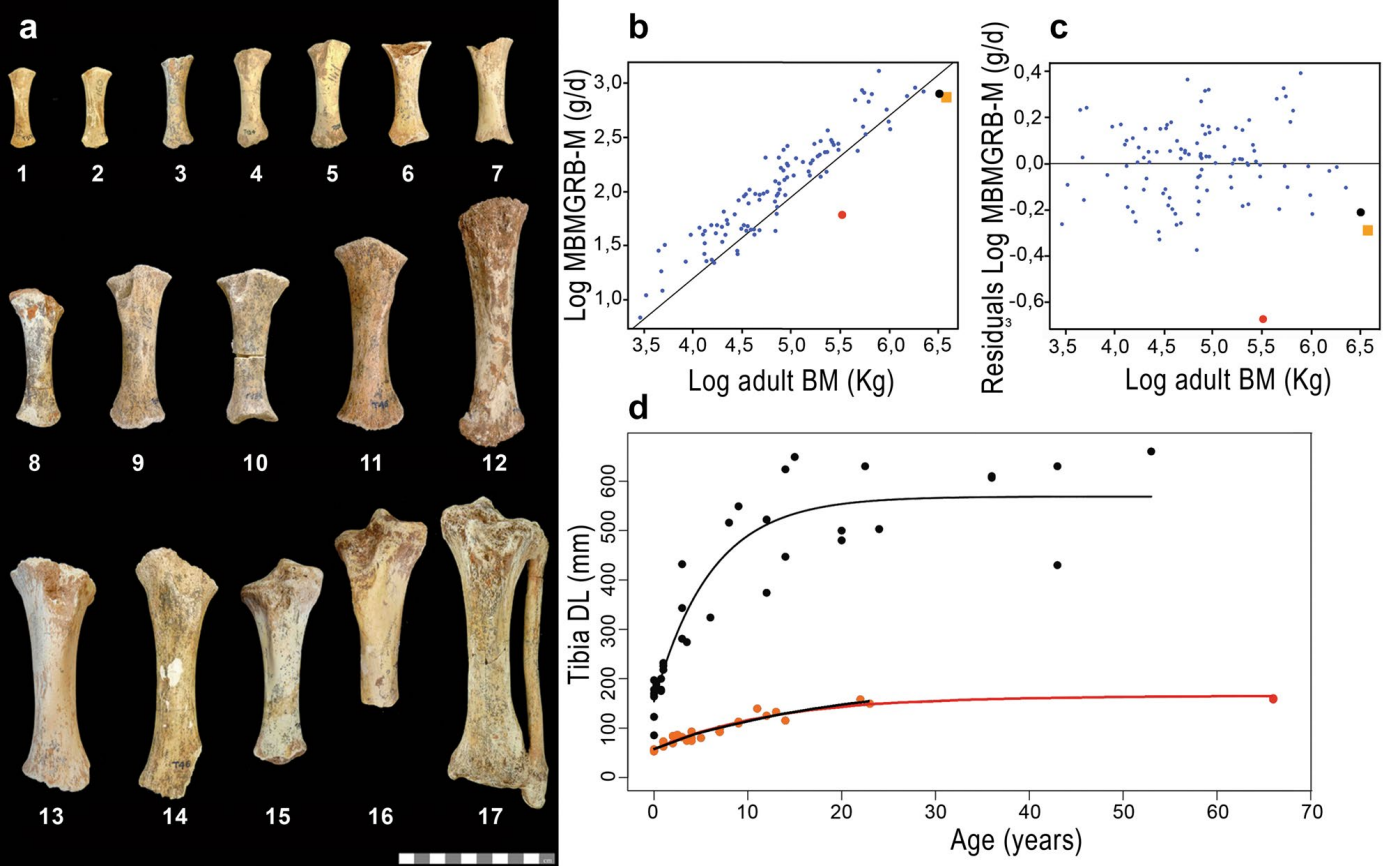
*P. falconeri* GR. (**a**) sample of the aged tibiae: 1,2: neonates (CAT-106; CAT-108); 3: one year (ELEPH1-T); 4: two years (CAT-124); 5: 3,5 years (CAT-128); 6,7,8: 4 years (CAT-132, Cat 133, ELEPH2-T); 9,10: 7 years (CAT 139, Cat 140); 11,12: 9 years (CAT-45, 142); 13: 12 years (ELEPH3-T); 14: 13 years (CAT-46); 15: 14 years (CAT-179); 16: 18 years (T-181);17: 23 years (Roma 6) (Graphic scale 10 cm). (**b**) Scaling of mean body mass GR from birth to maturity (MBMGR, expressed in grams/day) in respect to adult body mass, in log10, using phylogenetic generalized least square regressions (PGLS), in a large sample (n = 106) of species of ungulates (see Supplementary material 8 for results of PGLS). Blue dots: ungulates; red dots: *P. falconeri* (body mass from tibia LTC; Supplementary material 2; Supplementary Tables 2, 3), black dot: *Elephas maximus*; orange square: *L. africana*; data from PanTheria^33^. (**c**) distribution of residuals of the PGLS log10MBMGRB-M (adult-neonatal body mass, gr/age from birth in days) (y) regressed against log10 adult body mass (x). Extant elephants (orange square: *L. africana*, black dot: *E. maximus*) are within the lower limit of adult body mass-neonatal body mass GR in ungulates; *P. falconeri* (red dot) corresponds to the body mass estimation of tibia LTC (Supplementary material 2). (**d**) Von Bertalanffy growth curves of diaphyseal lengths of the tibiae (TDL) of *L. africana* (black dots and black curve) and *P. falconeri* (red dots; black and red curves). *P. falconeri* growth model 1 (short black curve) is based on ontogenetic data of specimens directly aged by skeletochronology (red dots). The long red curve corresponds to model 2 and is based on the inferred minimal longevity (Table 1; “Materials and methods”) and the maximal value of TDL.

**Figure 2:**
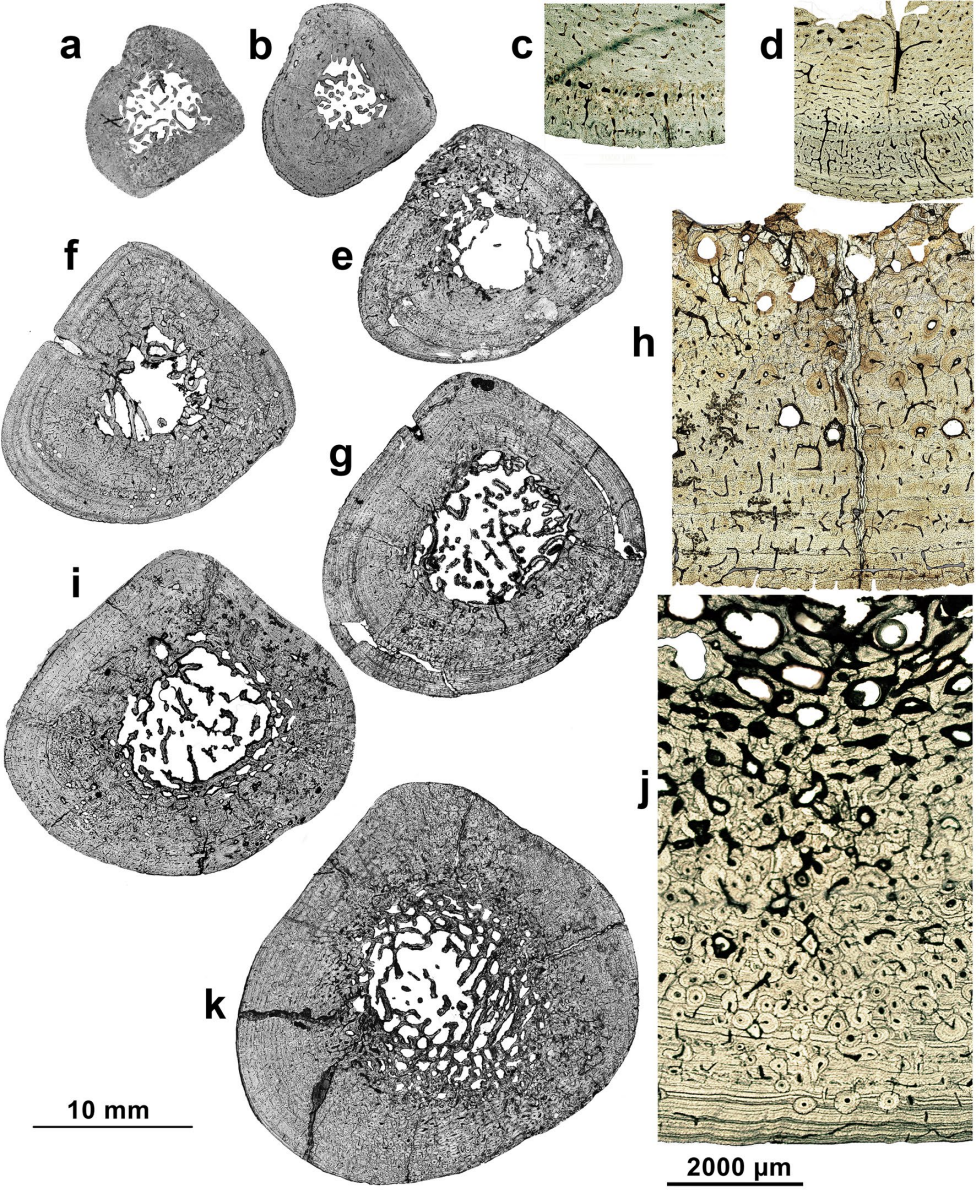
Tibia midshaft sections of *P. falconeri*. Representation of tissue types in histological sections of an ontogenetic series of tibiae ((**a**, **b**, **f**, **e**, **I**, **g**, **k**); anterior crest upper); magnifications (**c**, **d**, **h**, **j**; medullary cavity upper). (**a**) neonate (T106) showing parts of the birth line; (**b**, **c**) (T1): 1 year; longitudinal and radial osteons after the first winter LAG; (**d**) (T135), (**e**) (T138): 2.5–3 years ‘Fibro-lamellar complex’ (FLC) with longitudinal, oblique and radial osteons; (**f**) (T2): 4 years; (**g**) (T139): 7 years; (**h**) (T 45): 9 years, still showing active growth (open canals at the outer cortex); (**i**) (T142): 11 years; (**j**) (T 203): 22 years, showing an EFS; (**k**) (Roma6): 23 years;. Zeiss Scope A1 microscope with integrated digital camera (AxioCam ICc5), transmitted light. 10 mm scale: complete sections; 2000 µm scale: magnifications.

**Figure 3:**
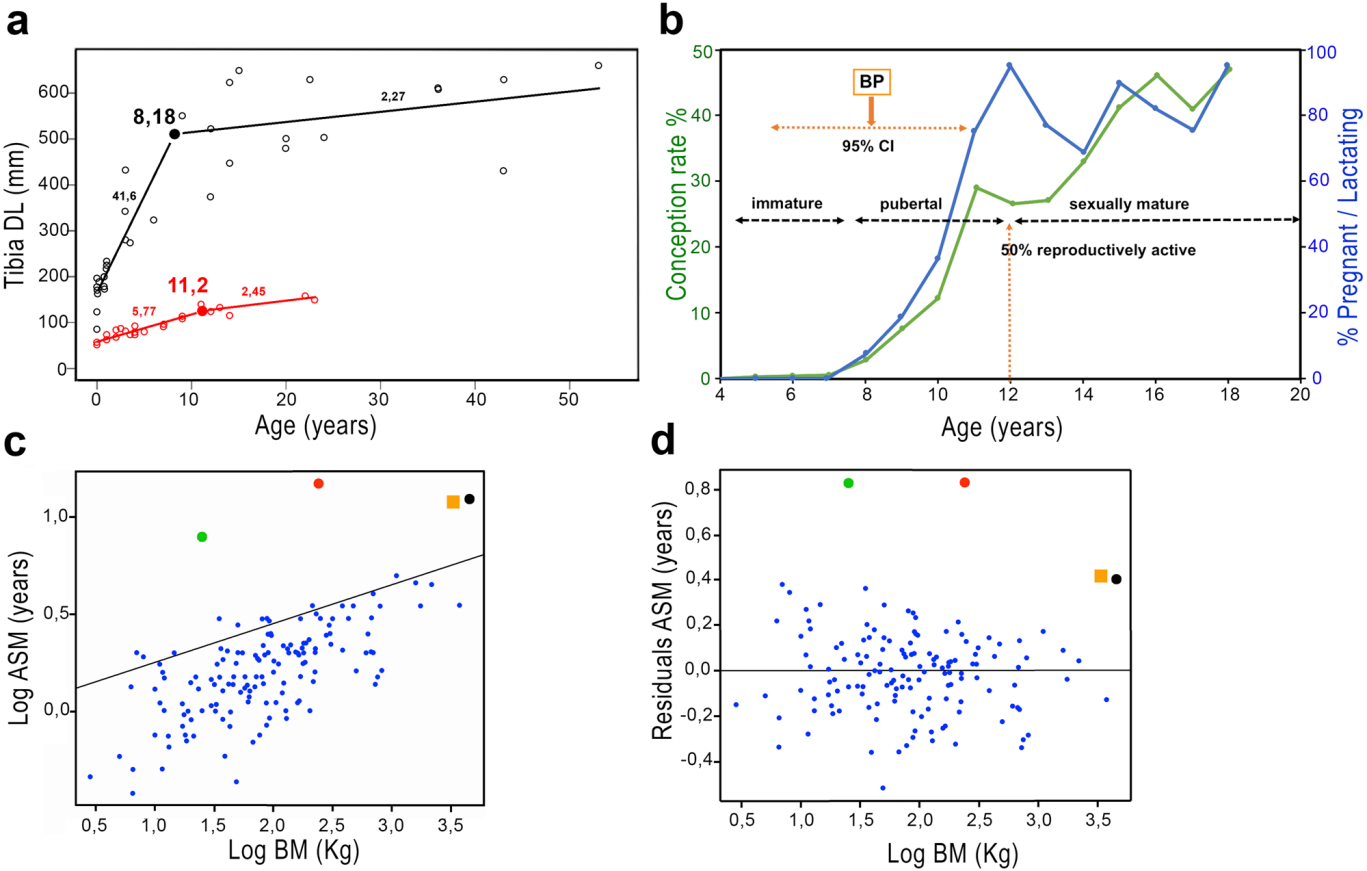
Inference of ASM in *P. falconeri*. (**a**) Piecewise regressions of tibia diaphyseal lengths (DL) against age (years) in *L. africana* and *P. falconeri*. Open black circles and black regression line represent *L. africana*; large black dot: break point (BP) with its value; open red circles and red regression line correspond to *P. falconeri*; large red dot: break point (BP) with its value; slopes of regressions are placed over the lines for each taxon (Table 2). (**b**) Ontogeny of the reproductive phases of *L. africana*, shown as age-specific conception rates and reproductive activity for a sample of 905 *L. africana* cows shot in the Krüger National Park (South Africa) between 1970 and 1974^34^, signaling the reproductive ontogenetic phases. Horizontal bar in the upper left corner indicates the timing of the break point (BP) obtained from DL of the tibia with the 95% confidence intervals (See “Materials and methods”; Supplementary material 6). (**c**) ASM: scatterplot of phylogenetic generalized least square regressions (PGLS) of log ASM (y) against body mass (x) for extant ungulates (n = 145) and elephants (Data base from AnAge^35^). Results of PGLS regression in Supplementary material 8. Blue dots: ungulates; red dot: *P. falconeri*; green dot: *Myotragus balearicus*^36^; black dot: *Elephas maximus*; orange square: *L. africana*; (**d**) Residuals of ASM in ungulates from PGLS regression of ASM against body mass. Blue dots: ungulates; Red dot: *P. falconeri*; Green dot: *Myotragus balearicus*^36^; Black dot: *Elephas maximus*; orange square: *Loxodonta africana* (Data from AnAge Data base^35^).

**Figure 4:**
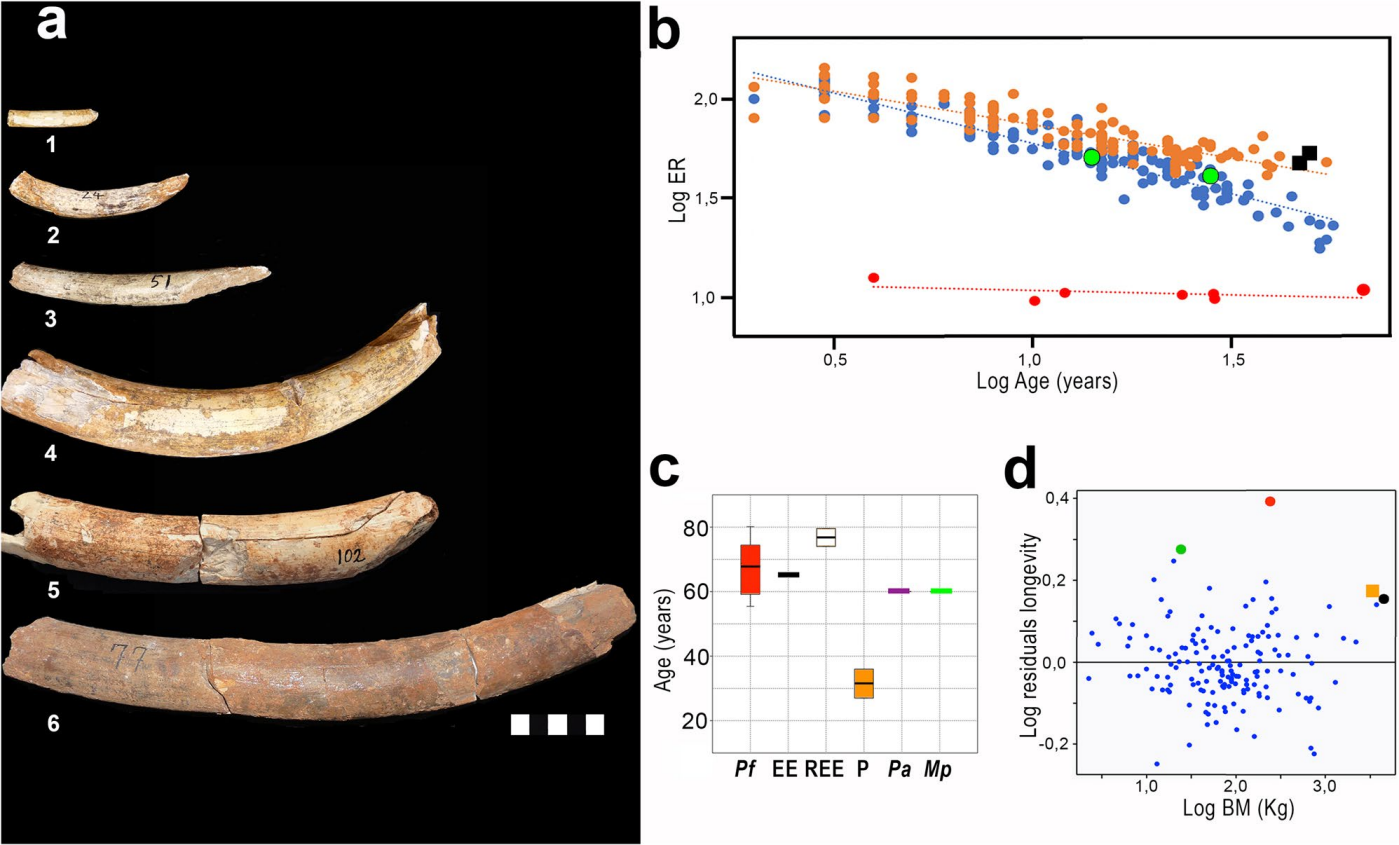
Tusk growth patterns of *P. falconeri*. (**a**) Spinagallo tusks sample analysed. 1: CAT-5; 2: CAT-24; 3: CAT-51; 4: CAT-100; 5: CAT-102; 6: CAT-77-76-78. Graphic scale 5 cm. (Supplementary material 5, Supplementary Table 5). (**b**) changes in ER over ontogeny (log-transformed age (x) and log of ER (y)) in *L. africana*^37^ and *P. falconeri*. Blue dots: *L. africana* females (y = − 0.5116x + 2.291, R^2^ = 0.88); orange dots: *L. africana* males (y = − 0.3402x + 2.2119, R^2^ = 0.77); red dots: *P. falconeri* (y = − 0.0446x + 1.0793, R^2^ = 0.22). Observe the low position of the *P. falconeri* tusk ER regression line and the low exponent, showing the small differences in ER between young and old individuals, which accounts for the stable ontogenetic growth pattern. Green dots *L. africana* specimens aged by Uno et al.^38^. Black squares correspond to tusks of *M. primigenius*^39^, *M. columbi*^40^ and *M. americanum*^41^ (**c**) Box-and-whisker plots comparing the lifespan inferred for *P. falconeri* (*Pf*) from the six tusks analysed, with the longevity values of extant elephants (EE) *L. africana* and *H. maximus* from AnAge data base^35^, and maximal records of longevity in extant elephants^42–46^ (REE); (P) lifespan predicted for *P. falconeri* from body mass allometry^23–25^; estimations of inferred lifespan for fossil large elephants *P. antiquus*^47^ (*Pa*) and *M. primigenius*^47^ (*Mp*). Horizontal lines denote the median, boxes the interquartile range (25–75 percent quartiles), and whiskers the maximum and minimum values; percentile ranks are computed by linear interpolation between the two nearest ranks. (**d**) Scatterplot of residuals of phylogenetic generalized least square regressions (PGLS) of body mass against longevity from the regression of ungulates (small blue dots); orange square: *L.* africana (body mass from Anage^35^); black dot: *E maximus* based on body mass^35^; red dot *P. falconeri*; green dot *M. balearicus* from the Pleistocene of the island of Majorca^36^. Results of PGLS regression in Supplementary material 8).

**Figure 5:**
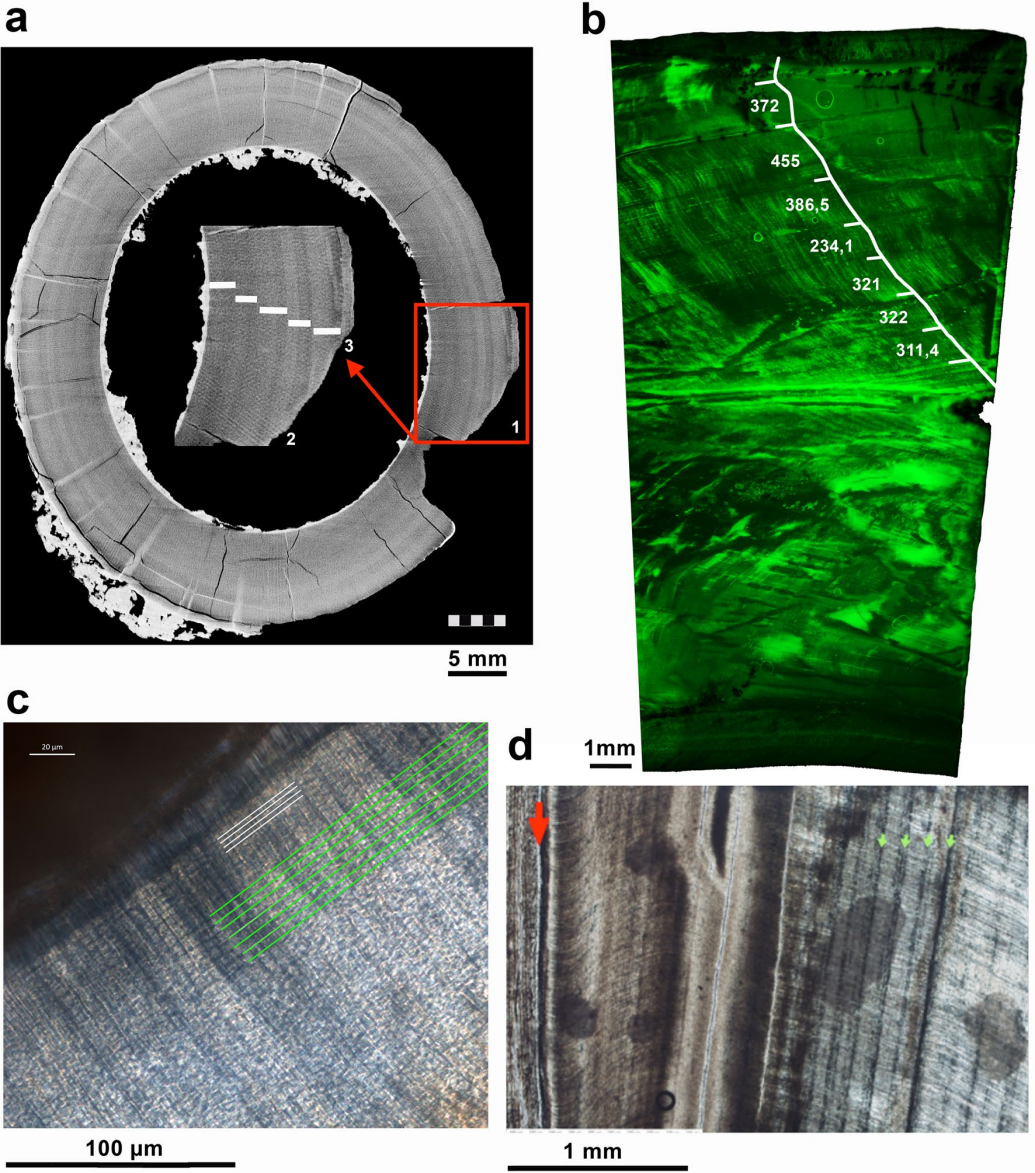
Tusk histology. (**a**) MicroCT—scan section of *P. falconeri* tusks. 1: Transversal microCT—scan section of CAT-102 tusk showing dark (winter) light (summer) bands; observe that the winter bands are thinner than summer bands. 2: magnification of (1) showing the last 5 annual incremental bands (pairs of dark–light dentine bands). 3: thin external cementum layer. (**b**) CAT-24 tusk (slide 24 B2-L1-110305.1) under green fluorescence light (for better visibility of the structures); measurements were taken between daily cross striations to calculate mean daily secretion rate (DSR); white line: prism; the prism was carefully followed as it decussated to estimate the days along the prism between the annual increments; white bars: key annual FOIs; the calculated number of days (white numbers) confirms the annual nature of the increments; Zeiss Scope.A1 microscope fluorescence light. (**c**) tusk 5 (slide 5-2–4) exemplifies the arrangement of daily increments (parallel green lines) and sub-daily increments (white lines) deposited along the border of the pulp cavity (dark area upper left). (**d**) CAT 100 tusk with monthly (second-order) increments; red arrow: cementum-dentine junction; (**b**) green arrows: second-order increments. Zeiss Scope A1 microscope with integrated digital camera (AxioCam ICc5).

**Figure 6:**
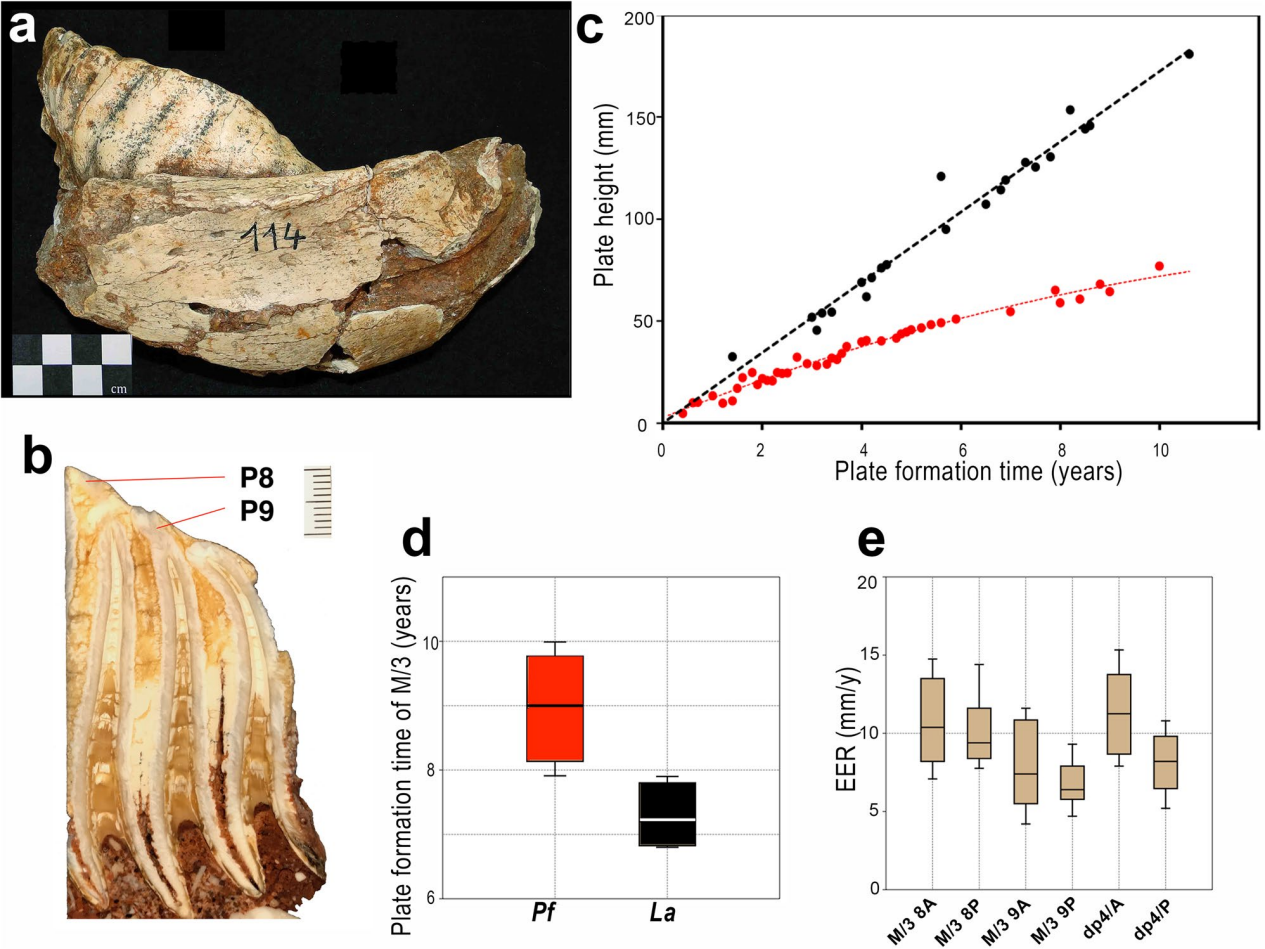
Molar growth pattern in *P. falconeri*. (**a**) Fragment of lower mandible with the *P. falconeri* M/3 (CAT-114) used in molar analysis (graphic scale 4 cm); (**b**) longitudinal section of the posterior part of M/3 (CAT-114) showing the measured plates 8 and 9 (Graphic scale 1 cm); it is still unworn, roots are closed. (**c**) Growth in plate height plotted against PFT (years) for upper and lower dentition together; black dots: continental elephants (*M. columbi*^32^; *P. antiquus* IPS-B1-L1-82167, the last supposed sister taxon of both insular taxa *P. cypriotes* and *P. falconeri*); red dots: insular elephants (*P. cypriotes*, Cyprus^32^; *P. falconeri*, Sicily). (**d**) Box-and-whisker plots for PFT of M/3 (red *P. falconeri*; black *L. africana*^38^). Mean values correspond to the mean of PFT of the lamellae of the different plates (Supplementary material 7). Horizontal lines denote the median, boxes the interquartile range (25–75 percent quartiles), and whiskers the maximum and minimum values; percentile ranks are computed by linear interpolation between the two nearest ranks. (**e**) Box-and-whisker plots for EER (mm/y) of the different *P. falconeri* lamellae and plates studied; EER decreases posteriorly. Horizontal lines denote the median, boxes the interquartile range (25–75 percent quartiles), and whiskers the maximum and minimum values; percentile ranks are computed by linear interpolation between the two nearest ranks (Supplementary material 7).

**Figure 7:**
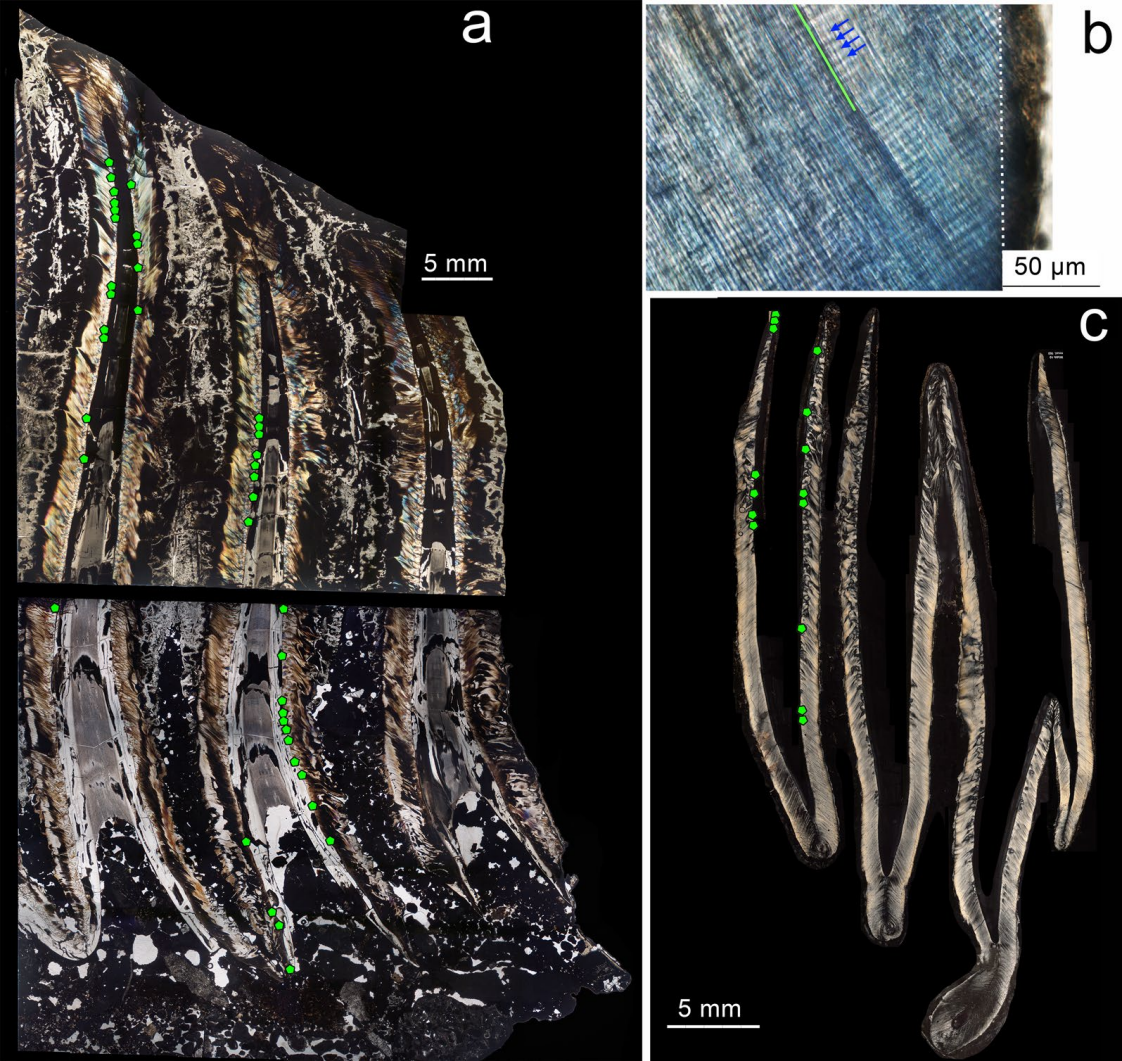
Molar histology of *P. falconeri*. (**a**) Composed slide of the third lower molar (CAT-114, slides IPS 96,003). The tooth is cut horizontally (3 mm lost); it shows the measured plates 8 and 9 (plate 10 was taphonomically heavily cracked and not measurable) with the location of our measurements (green dots). Depending on the state of preservation of each plate, we took 29 measurements on the upper and 25 on the lower half (there are less points because some of them are too close together as to be separated). (**b**) (slide IPS 85787_3a) illustrates cross striations (blue arrows) along an enamel prism (green), and the enamel-dentine junction (dotted white line) in a lower second molar. (**c**) unworn Dp4/superior (slide IPS-85040; Supplementary material 7) with green dots signaling location of 16 measurements (there are less points because some of them are too close together as to be separated). Zeiss Scope A1 microscope with integrated digital camera (AxioCam ICc5).

To reconstruct growth pattern and LH schedule, an analysis of ontogenetic GRs in an allometric context is mandatory. Though elephants are known to have the slowest relative GRs^29^, the results of our analysis show that, despite its small size, *P. falconeri* grew at even slower relative rates than his continental cousins, with a peculiarly small difference in GRs between pre- and post-sexual maturity. We used different anatomical parts (tibiae, molars, and tusks) and different methodological approaches to infer GRs for *P. falconeri*. Thus, based on the ontogenetic growth of tibial diaphyses (Figs. 1a, 2; Supplementary material 3, 4; Supplementary Table 4; Supplementary Fig. 1), we found an extremely low scaling of the accumulated mean specific body mass GR from birth to maturity (MBMGRB-M), compared with other ungulates, including extant elephants (Fig. 1b, c; Supplementary material 3, 4). In accordance with the inferred slow juvenile GRs in *P. falconeri*, piecewise regression of the ontogenetic increase in length of the tibial diaphysis shows only minimal differences in the slopes of pre- and post-sexual maturity (5.77–2.45), which contrasts with the important differences in *L. africana* (40.62–5.77) (Fig. 3a; Table 1). Similar results have been obtained using von Bertalanffy growth models (Fig. 1d; Table 2). The value of the growth constant K in *P. falconeri* is far lower (0.055–0.079) than that of *L. africana* (0.1666) (see “Materials and methods”, Table 1). A similar pattern of GR is observable in the dentition. Tusk histology provided the most unexpected result of this study: the absence of relevant differences in GR over ontogeny (Figs. 4a, b, 5; Supplementary material 5). The overall tusk GR is even lower than in senescent extant *Loxodonta*, where it varies from 140 mm/y in young individuals to 20–40 mm/y in senescent females/males. Young individuals of *P. falconeri*, however, had a mean GR of 12.5 mm/y while the oldest grew at 10.4 mm/y (general sample mean 10.16 mm/y) (Fig. 4a, b; Supplementary material 5). Despite the statistically significant albeit very small differences between juvenile and adult tusk DSRs (Supplementary material 5, Supplementary Fig. 2, Supplementary Tables 8, 9), a trend towards uniformity in DER among tusks emerges from our analysis (Supplementary Figs. 3, 4, Supplementary Table 10). The scatterplot of log-transformed age (years) and log of extension rate (years) in P. *falconeri* (y = − 0.0446x + 1.0793, R^2^ = 0.22) shows the very low exponent, expressing the lack of significant differences in DER between young and old individuals (Fig. 4b). The minimal change in tusk GR over ontogeny parallels the small differences in postcranial GRs during pre- and post-sexual maturity (Supplementary material 6; Figs. 1d, 3a). A similarly slow GR is observable in molars (Figs. 6c, 7). Plotted against PFT, the molar plates grew consistently slower in height in *P. falconeri* (and in *P. cypriotes*) than in continental elephants (*Mammutus columbi*, *P. antiquus*) (Fig. 6c; Supplementary material 7). Accordingly, insular dwarf taxa combine a slower rate of dental growth (plate height against PFT) with an unchanged time of formation. When postcranial (tibiae), tusks and molars are considered together, a pattern of systemic slow GRs emerges for *P. falconeri* compared with the element- and age-dependent variable GRs in *L. africana*.

**Table 1:** Statistical results from von Bertalanffy growth model for diaphyseal length of the tibia (DL) of *P. falconeri* and *L. africana*.

*P. falconeri*	K						
	R2	W-Wrt	Est	CI 95%	SE	t value	*p*
TDL (1)	0.997	− 1.60 (*p* = 0.10)	0.055	0.021–0.113	0.018	6.792	55.03E–07
TDL (2)	0.939	0 (*p* = 1)	0.079	0.059–0.103	0.012	6.504	8.22E–07
***L. africana***							
TDL	0.871	0 (*p* = 1)	0.166	0.108–0.248	0.037	4.464	8.85E–05

*P. falconeri*	L inf						
	R2	W-Wrt	Est	CI 95%	SE	t value	*p*
TDL (1)	0.997	− 1.60 (*p* = 0.10) 0 (*p* = 1)	192.75	160.20–327.5577	28.380	6.790	0.000
TDL (2)	0.939	0 (*p* = 1)	165.00	152.54–182.63	7.761	21.260	< 2E–16
***L. africana***							
TDL	0.871	0 (*p* = 1)	568.82	527.47–617.92	23.100	24.620	< 2E–16

Considering that in the case of *P. falconeri*, the equation (model 1) only includes data from a segment of the ontogeny that covers the period from birth until the recorded age (growth cessation, 23 years in our *P. falconeri* sample), we provided a second model (model 2) based on the measured ontogenetic trajectory of tibiae (model 1) plus the minimum longevity inferred for *P. falconeri* from the longest available tusk to make the VBGM of both taxa comparable (See “Materials and methods”; Table 3; Supplementary material 3, 4).

**Table 2:** Piecewise regressions of tibia diaphyseal lengths of *L. africana* and *P. falconeri.*

TAXA	Breakpoint			R^2^	*p*-score test (1)	Regression equation	
	Estimated value	L CI 95%	UCI 95%			Pre-breakpoint	Post-breakpoint
*L. africana* TDL	8.18	5.32	11.03	0.8744	7.79E–08	LTDL = 166.98 + 41.621*Age	LTDL = 488.91 + 2.237*Age
*P. falconeri* TDL	11.02	6.51	15.52	0.9332	0.003321	LTDL = 60.096 + 5.771*Age	LTDL = 96.67 + 2.453*Age

(1) If *p*-value < 0.05, the differences between slopes are significant. LCI lower confidence interval, UCI upper confidence interval.

ASM is a key fitness component of particular importance for population growth rate *r*^48^. Decelerated ontogenetic growth is usually associated with a delay in ASM along the slow-fast LH axis^7,49^. Applying piecewise regression to an ontogenetic data base of diaphyseal lengths of tibiae of *L. africana*^50^, (Fig. 3a) we found two slopes with an inflection point at 8.18 years that separates the early juvenile phase of fast growth from a later phase of slow growth. The placement of the breakpoint (BP) within the ontogenetic process of sexual maturation in the sample of *L. africana* from Kruger National Park^34^ suggests that BP occurs before sexual maturity and first calving, coinciding approximately with the pubertal phase of sexual development (Fig. 3b). Thus, BP coincides with the point at which energy is diverted from somatic growth to reproduction (Fig. 3a). In *P. falconeri*, this BP is at the age of 11 years, 3 years later than in *L. africana*. Because mean ASM is at 12 years in *L. africana*, 4 years later than BP in this species, we infer by analogy that mean ASM in *P. falconeri* was close to 15 years. Compared with other ungulates, elephants attain sexual maturity relatively late even when considering their elevated body mass as show phylogenetically corrected ASM / BM regression and residuals (Fig. 3c, d). *P. falconeri*, however, like the insular dwarf bovid *Myotragus balearicus*, is a complete outsider attaining sexual maturity much later than predicted from body mass (Fig. 3c, d). This result is supported by the similar age of epiphyseal fusion, despite the size differences between *L. africana* and *P. falconeri* (Supplementary material 6; Supplementary Fig. 6). Indeed, the earliest evidence of the beginning of the process of epiphyseal fusion in *P. falconeri* tibiae is at 14 years (proximal epiphysis T179), comparable to the first evidence of epiphyseal fusion in tibiae recorded for female *L. africana* (15 years). Several *P. falconeri* individuals at age 22, however, still show mixed patterns from no fusion at all over only proximal fusion to beginning of distal fusion. In male *L. africana*, several individuals still have unfused epiphyses proximally and distally in their early-mid twenties^50^. Thus, *P. falconeri* does not differ in timing of epiphyseal fusion of tibiae from extant *Loxodonta* despite the important size differences. Data on the age at fusion of tibial epiphyses in *P. antiquus* and *M. primigenius* are less well known; however, current evidence suggests a similar pattern^50,51^.

A delayed ASM is commonly associated with an increase in lifespan^8,48,52–54^ in accordance with theory^52,53^ and empirical evidence for early-late life trade-offs in the wild^55^. By reconstructing tusk GRs in 6 Spinagallo tusks (Fig. 4a, b), we calculated the minimum life expectancy of *P. falconeri* from the length of the largest known tusk from Spinagallo (Fig. 4c, d; Table 3; Supplementary material 5). Because tusks are reduced by wear and accident over lifetime, our values give a minimum age at death for this old individual. The time to form this tusk provided a minimum absolute age for *P. falconeri* (67.97 y) similar to that of extant elephants and older than that of both its ancestor *P. antiquus*, and *M. columbi* (Fig. 4c; Supplementary material 5) and by far older than that predicted from body weight^23,24^. Phylogenetically corrected residuals of least-square regressions of lifespan against body mass indicate that *P. falconeri* had a proportionally much longer lifespan than extant large elephants when body mass is taken into account (Fig. 4d; Table 3; Supplementary material 5, 8).

**Table 3:** Predicted and residual values for maximal longevity against body mass in extant proboscideans, *P. falconeri* and one ungulate insular taxon, *Myotragus balearicus*^36^.

TAXA	BM kg	Maximal longevity (years)	Predicted	Actual-predicted
*E. maximus*	4500	65.5	51.90	13.60
*L. africana*	3350	65	49.90	14.14
***P.falconeri***				
*Mean*	242	67.96	27.37	33.40
95% CI L	141	62.55	24.91	30.11
95% CI U	328	73.35	29.63	36.11
*M. balearicus*	25	35	25.65	9.35

Results of PGLS longevity for extant ungulates (n = 155; data from AnAge data base^35^) are: Y = a + bx, Intercept (a) 1.21941, slope (b) 0.135772, Residual standard error: 0.01342 on 153 degrees of freedom, Multiple R-squared: 0.3746, Adjusted R-squared: 0.3706, F-statistic: 91.66 on 1 and 153 DF, *p*-value: < 2.2e–16. The values of *P. falconeri* are the mean lifespan and the 95% CI, for the BM of *P. falconeri* L: lower bound, U: upper bound (Supplementary material 5, 8, Supplementary Figs. 12, 13).

The plots of phylogenetically corrected residuals reveal that *P. falconeri* is a clear outlier for all LH parameters here analysed (see Supplementary material 8, Supplementary Figs. 12, 13, 14, Supplementary Table 13). Its LH shifted towards the slow end of the slow-fast LH continuum independently of phylogeny.

## Discussion

Our various histological approaches to bone, molar, and tusks growth combine to provide solid evidence that *P. falconeri* reduced its body size by decreasing *growth rate* and not *growth period* as previously claimed. Even more, it reduced *growth rate* and *extended growth period*, delaying related LH traits *beyond* the values expected from body mass scaling in continental ungulates. Indeed, in line with the extreme nature of its insular dwarfism, *P. falconeri* appears to have the lowest relative values of body mass GR ever recorded for any ungulate or elephant species (Fig. 1c, d; Supplementary information 4, 8; Supplementary Figs. 8, 9); it attained maturity much later than predicted from interspecific scaling models and even later than large extant *Loxodonta* (Fig. 3; Supplementary information 7; Supplementary Figs. 10, 11; Table 2); and it was at least as longevous as its large putative sister taxon *P. antiquus*^47,50^ and other large continental elephants (*L. africana* and *Mammuthus*^47^) (Fig. 4c; Supplementary information 5; Supplementary Figs. 12, 13; Table 3). Thus, *P. falconeri* was an insular dwarf with an exceptionally low GR, delayed maturity, and an extended lifespan. Our study, hence, shows that *P. falconeri* occupies the slow end of the slow-fast LH continuum, debunking the widely held notion from LH/body size scaling that dwarfing on islands results from growth truncation associated with a shift towards a fast LH^21,23,24,26,27,56^, but in line with both mortality-driven LH models and theories of aging.

Optimization models for age and size at maturity predict that under most conditions^3^ optimal ASM increases as GR decreases^2–4,8^; when juvenile mortality is independent of GR, slower growth is expected to result in a delay in maturity associated with a smaller body size^2,3^. On islands, absence of mammalian predators in fossil^57^ and extant^19^ insular communities allows relaxation of GRs in juvenile large mammals; there is no need to accelerate GR to increase the chance of survival to maturity by escaping a risky size range. This strongly suggests that juvenile mortality on islands, at least in large mammals, is uncorrelated with the growth coefficient, a premise for delay in maturity at a smaller size (see also^2^).

The phenotype of *P. falconeri* is consistent with the predictions from optimization models as the important decrease in GR led to a delay in maturity much beyond expectations from allometric scaling, at an extremely small body size. Our results are also in accordance with theories that focus on the evolution of aging as a particular aspect of LH theory (mutation accumulation^58^, antagonistic pleiotropy^59^, disposable soma^53,60^), which predict a decrease in extrinsic mortality to select for evolution of longer lifespans; these predictions are supported by a growing number of experimental and comparative field studies^6^. *P. falconeri* is not an exception as, under absence of predation, its lifespan far exceeds expectations from body size scaling. Indeed, in accordance with phylogeny-corrected comparative studies of ageing, *P. falconeri*’s LH phenotype matches that of other outliers from body size allometries evolved under less hazardous environmental conditions with lower mortality rates, such as bats or subterranean animals, which combine small size with a slow GR, an extended developmental phase, and a longer longevity^52,60^.

In contrast, earlier ASM at a smaller size and short lifespan are predicted to evolve when juvenile mortality increases^3^, and the chance to survive to reproduce at later age is low (e.g. under elevated extrinsic mortality)^8,52^. Increased juvenile mortality has previously been invoked by Raia et al.^23^ (see also^26^ for *P. cypriotis*), based on the age structure of the fossil assemblage from Spinagallo cave^23,61^ where 56.7–57% of *P. falconeri* tibiae had detached epiphyses (e.g. were somatic ‘juveniles’). This pattern, represented by an L-shaped mortality profile^62^, is the ‘catastrophic’ model in which successive age classes contain progressively fewer individuals, reflecting the structure of a living population from which demographic structure can be inferred. To reconstruct the mortality profile of *P. falconeri* (Supplementary material 9; Supplementary Fig. 14), we used eruption and wear pattern of the dentition, which perfectly depicts all ontogenetic stages, instead of unsectioned tibiae, which only differentiate between pre- and post-skeletal maturity and have a low degree of resolution of the various ontogenetic stages. Our mortality analysis of the Spinagallo site (Supplementary material 9; Supplementary Fig. 8) indicates a different, U-shaped mortality profile with a moderately high representation of juveniles (47%), low representation of prime-age adults, and an elevated number of old-senescent individuals. This profile is in line with earlier work by Ambrosetti^61^. It fits the ‘attritional’ type, in which the very young and the old are best represented^62^, characteristic of fossil sites that cover an extended stratigraphic period, reflecting mortality due to accidents, predation, endemic diseases, and other factors that ordinarily have their greatest impact on the very young and the old. Thus, while it is difficult to reconstruct the taphonomic conditions under which the Spinagallo Cave *P. falconeri* material accumulated with certainty, the age structure of the fossil assemblage is not at odds with a low juvenile mortality environment.

Why do LH predictions from allometric scaling apparently fail for insular dwarfs? One reason for this is that LH strategies evolve along a slow-fast continuum independently of body size^48,63^. Key LH components, e.g. ASM, instead consistently correlate with patterns of extrinsic (particularly juvenile^3^) mortality when the effect of body size is controlled for^48^. Large-scale interspecific allometric studies are typically based on balanced ecosystems (large continental areas) with high levels of predation, where short growth periods increase reproductive fitness by decreasing generation time; body mass—LH trends are therefore driven by abundance of high extrinsic mortality/short LH taxa. As such, large-scale interspecific allometric studies are a poor predictor for LH traits in low mortality insular settings. While under most, generally continental, conditions natural selection targets developmental time and truncation is expected to be the predominant process behind dwarfing (increased extrinsic juvenile mortality leads to earlier maturity at a smaller size^3^), in insular environments with low mortality risk fitness benefits of fast growth (short generation time^4,8^) dwindle; instead, a decrease in GR maximizes individual fitness in those (usually larger mammalian) species for which low resource availability is a problem and small adult size is expected to prevail, as is the case of other typical outliers from body size allometries^52^.

## Conclusions

Our results provide evidence that *P. falconeri* did not shift towards the fast end of the LH continuum by truncation of the growth period, as widely claimed for insular dwarf elephants (*P. falconeri*^18,21,23–25^ and *P. cypriotis*^26^) and dwarfed slow-growing dinosaurs (*Telmatosaurus* and *Zalmoxes*^27^). Instead, they agree with the finding of similarly slow molar GRs in the other dwarf Mediterranean elephant, *P. cypriotis*^32^, and with LH traits seen among other insular dwarf mammals such as *Myotragus balearicus*^64–66^ and extant and fossil insular cervids^67–69^. In fact, the only empirical evidence for early onset of reproduction in an insular dwarf mammal comes from introduced feral Amsterdam Island cattle^70^ and is considered to result from artificial selection^71^. In the light of LH theory, our findings are consistent with mortality-centered demographic LH models, which predict body size reduction under low extrinsic mortality (and limited resource availability) to be associated with a shift towards a slow LH^2–4, 8^. This suggests dwarfing associated to a decrease in GR and a concomitant delay in age at first reproduction combined with an increase in lifespan, to be the rule in fossil and extant insular mammals. *P. falconeri* is an excellent example of the evolutionary change towards a slow LH that accompanies the process of insular dwarfing.

## Materials and methods

### A general statement about limitations

Sample size is the most evident and pervasive limitation in paleontology. The number of specimens available is usually low. In histological studies that typically involve the destruction of the specimens, the number of available specimens is even more constrained because of the reluctance of curators to authorize cutting large samples of fossil specimens. Therefore, we tried to obtain the largest sample size possible, which includes an ontogenetic series of 30 tibiae (only 29 of them were useful), two complete check teeth—M/3 and dP4/, and an ontogenetic series of eight tusks (only 6 of them were useful). This material represents the largest histological sample of a proboscidean species ever studied.

### *P. falconeri* postcranial material

To establish the age of epiphyseal fusion and the beginning of deposition of EFS by skeletochronology, we sampled an ontogenetic series of 29 tibiae (Figs. 1, 2; Supplementary Fig. 1; Supplementary material 3; Supplementary Table 4) (University of Catania Museum and Earth Science Department, Sapienza University of Rome). 24 of the tibiae were from the right side, and 5 were from the left side; left tibiae were different in size to any of the right tibiae to avoid using the same individuals.

### Preparation of histological thin sections

Histological slices of tibiae were prepared following standard methods in the histology laboratory of the Institut Català de Paleontologia Miquel Crusafont (ICP, Catalonia, Spain)^72,73^. The sample comprises 65 slides of 29 tibiae. For thin sections, a chunk of 3 cm was cut at the midshaft of each tibia, perpendicular to the long axis of the bone. This piece was then embedded in epoxy resin (Araldite 2020), forming a cubic block. Particular care was taken to ensure that the piece of bone was embedded in such a way that the subsequent cutting could be done at a right angle to the longitudinal axis of the bone. This block was then cut into two halves with a low-speed diamond saw (IsoMet, Buehler), perpendicular to the axis of the bone. The surfaces were polished with carborundum powder or with a MetaServ 250 (Buehler) and fixed to a frosted glass with the ultraviolet curing adhesive Loctite 358. The sample glued to glass was cut and ground with a diamond saw (PetroThin, Buehler) and again polished to a final thickness of approx. 100 ± 130 µm either with carborundum powder or with a grinding/polisher (MetaServ 250, Bühler). Lastly, the finished thin sections went through a final check and were then covered with glass in order to improve visualization of the histological features under the microscope. It was not possible in all cases to grind the slides to this thickness, as some of the bones were extremely brittle and would have been destroyed by further grinding. Thus, these bones were ground to a thicker final size (of up to 170 µm in two cases: IPS-96003, IPS-96266).

### Intra-/inter-observer error

Tibiae: LAG counts and area measurements between LAGs were repeated twice or more for each tibia; two of the authors (M.K. and S.M-S.) calculated the number of LAGs independently using the same thin sections.

Tusks: measurements of DSR and DER, as well as counting the number of TOIs, SOIs and FOIs, were taken on all tusks and repeated several times (M.K.); two of the authors (M.K. and S.M-S.) calculated the number of increments independently using the same thin sections.

Molars: measurements of ESR and EER were repeated several times on each plate (M.K.) at the same points.

The percentage error was very low, less than 1%, probably consequence of the good quality of both the thin sections and the micrographs.

### Skeletochronology and measurement of areas between LAGs

**Figure 8:**
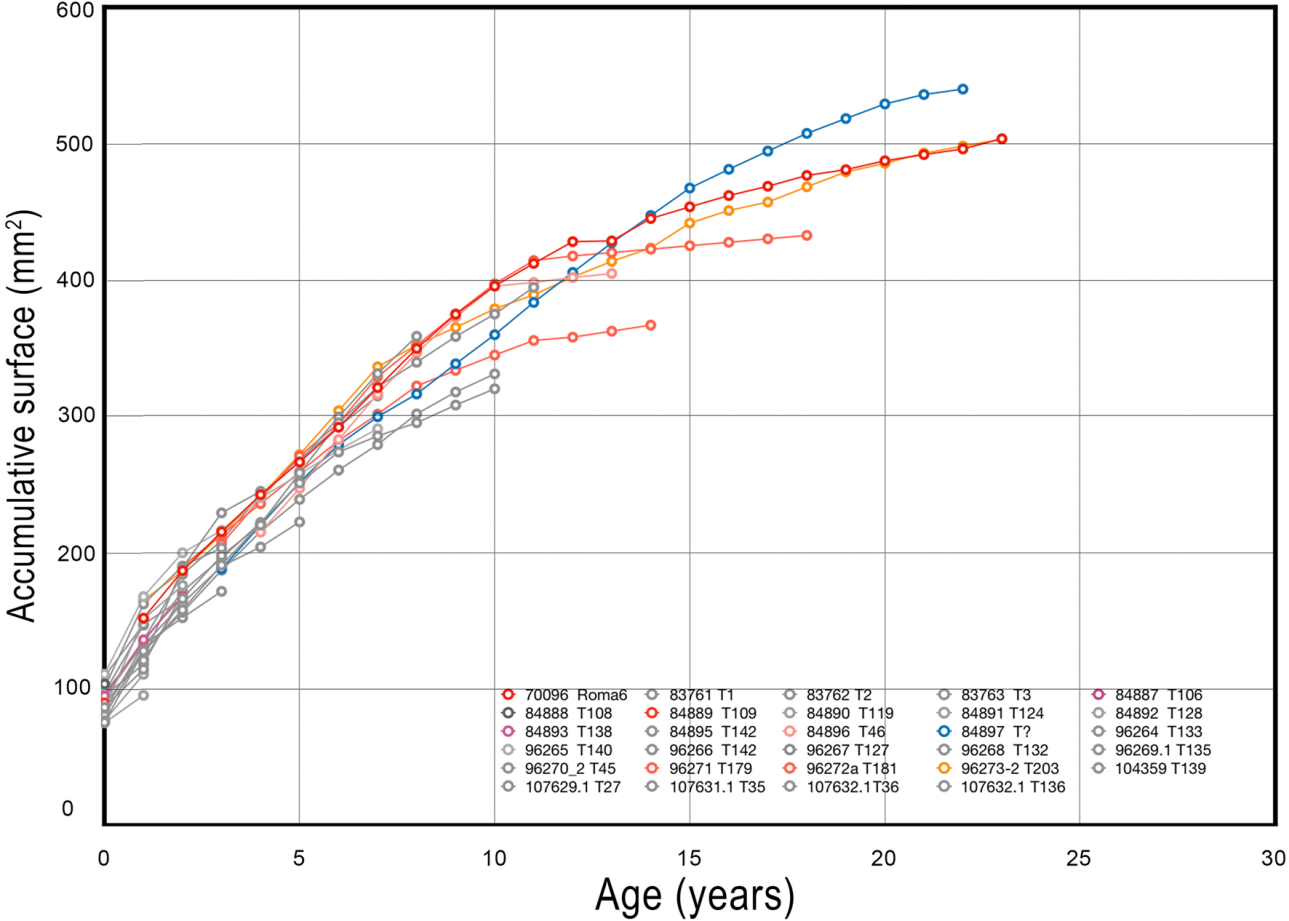
Accumulative bone area in transversal sections of 29 *P. falconeri* tibiae up to the age of 23 years when growth in bone circumference almost completely ceases. For simplicity, each consecutive annual surface area was calculated including the area of the medullary cavity (see “Materials and methods”). Grey: juveniles that died before onset of puberty; various shades of red and blue: individuals that survived onset of puberty. Individuals older than 15 years (adults) were likely much older than indicated by their last countable LAG because of the almost complete cessation of appositional growth a few years after onset of SM. Thus, for instance, small-sized (probably female) individual 70,096 (Roma6) shows a complete fusion not only of epiphyses but even of tibia and fibula.

We used histological slides for skeletochronology to determine the age at key LH events (birth, age at skeletal and sexual maturity and age at death) in *P. falconeri*. An in-depth description of bone histology in *P. falconeri* was beyond the scope of this study and is the focus of ongoing work. In contrast to most other ungulates, but similar to extant elephants, *P. falconeri* shows only insignificant to moderate expansion of the medullary bone cavity. Therefore, even in older individuals, LAGs formed at early ontogenetic stages are well preserved, including the neonatal line which remains partially preserved if not deleted by Haversian systems at older age. Except for the neonatal line, which records a single non-cyclical LH event^72^, we assumed LAGs to form annually. Infrequent sets of two (rarely three or more) closely spaced LAGs were counted as a single event. Until the age of approximately 4–5 years, all LAGs can be traced over their full extent. At later ages, LAGs are interrupted by Haversian systems that become increasingly denser invading more and more the external part of the bone (Figs. 1, 2, 8; Supplementary material 3, Supplementary material Fig. 1). Here, we reconstructed the LAGs by copying the next youngest rest line and expanding it with Photoshop CS6 (version 13.0) using ‘Free transform—Warp’ to adapt the outline to the still visible traces of the LAG. To reconstruct the outer LAGs in old individuals, we reduced the outline of the section with Photoshop CS6 (version 13.0) using ‘Free transform—Warp’ to adapt it to the visible traces of the younger LAG to reconstruct. While near complete and well-distinguishable LAGs were found until the age of approximately 12–14 years, the age at which the EFS begins to form, later deposited LAGs (within the EFS) often fused with one another and were only countable where bone was still deposited in sufficient quantity and, hence, where resolution remains acceptable (e.g. anterior or lateral crests; Fig. 2; Supplementary material 3). Thus, ages older than 12–14 years are estimated by counting these LAGs within the EFS to obtain the minimum age at death. We measured the cross-sectional areas between LAGs instead of radial distances from medullary cavity to outer surface, because 2-dimensional cross-sectional area is a better proxy of to body mass than 1-dimensional linear measurements. To do this, we selected the complete areas delineated by each consecutive LAG including the area of the medullary cavity using Photoshop C6 (select-color range). Next, we estimated the pixels and translated these into mm^2^. Inclusion of the area of the medullary cavity avoids introducing errors produced by changes in the extension of the medullary cavity through ontogenetic time and provides the real increase in area from one LAG (= year) to the next LAG (= year) (Fig. 8).

### External measurements

We measured tibial shaft lengths, least circumference and anteroposterior diameter at the mid-shaft (measures in mm) (Supplementary Table 1), as well as tusks and molars with a digital caliper.

### Methodological approaches to GR patterns

We used different approaches to analyse GR patterns of tibiae. Each approach offers a different perspective, allowing us to explore different aspects of the growth pattern of *P. falconeri*.

### Growth models

The use of growth models to infer ASM is a common method in palaeontology^74–76^. The inflection point in the growth curve, marking the transition from high to low GRs, has been used to estimate ASM. The von Bertalanffy growth model (vBGM) provides a good description of somatic growth after maturity but not before when GRs are highest^77–79^, while standard growth models require a priori fixation of the inflexion point (e.g. at 30% of the asymptotic growth curve in the case of the vBGM^75,80^). As we want to identify the timing of the inflexion point, we chose piecewise regression (segmented regression), which instead allows detection of such inflection points (breakpoint, BP) separating two (or more) segments. Piecewise regression (Fig. 3, Table 2) is frequently used in ecology and other biological fields^81^ and is more recently applied to estimations of size- and age-related mortality rates in fish, and the study of growth in fishes and other marine animals^82^. Models of biphasic somatic growth are used to detect the 'breakpoint' corresponding to the decrease in somatic growth occurring at maturation^76^ due to the allocation of energy to reproduction^77,78^. This growth model is considered to provide a better fit to data of growth involving ASM than the classical vBGM model^77,78^, an additional advantage to the fact that piecewise regression does not determine an a priori inflection point. Considering that juvenile growth in length (pre-sexual maturity) in biphasic models is a linear function^77,78^, the use of piecewise regression is appropriate to infer ASM from growth data. Therefore, we used this method to infer ASM from the ontogenetic trajectory of tibia diaphyseal length (Fig. 3a). Picewise regressions were obtained using ‘segmented’ packages in R studio (Version 1.2.1335). In extant species such as elephants, evidence of ASM and other reproductive parameters is usually obtained by direct observations and studies of the reproductive organs of culled populations^34,83,84^. Using the ontogeny of the same anatomical parameter (e.g. tibia diaphyseal length) in extant and fossil taxa and calculating the respective piecewise regressions, a homologous and adequate comparison is possible, as in both cases the same methodology and anatomical element are used. Thus, we applied piecewise regression using the database of tibia diaphyseal length and ontogenetic age of extant *L. africana* and our data of *P. falconeri*. We then compared the results from *L. africana* tibiae with the information on sexual maturity in living elephants, to interpret the biological meaning of the inflection point (BP in piecewise regression) in extant, and –consequentially- fossil, elephants (Fig. 3a, b).

### Growth parameter (K)

In the previous section, we discarded the use of vBGM to infer ASM in *P. falconeri*. Nevertheless, we applied vBGM to analyse GR patterns based on the calculated ages and the length of the tibia to infer the growth parameter (K) of *P. falconeri* and *L. africana*. For *P. falconeri*, we proposed two different models to make comparable the growth trajectories with that of *L. africana*. The first model (model 1) only includes data from a segment of the ontogeny that covers the period from birth until the end of the age record (growth cessation, 23 years in our *P. falconeri* sample). The second model (model 2) includes the longevity inferred for *P. falconeri* (Fig. 1d; Table 1; Supplementary material 5), thus making comparable the VBGM of both taxa, including in this case the complete ontogenies of both. In any case, the parameters of both trajectories are very similar (Table 1). vBGM was obtained using ‘FSA’ and ‘nlstools’ packages in R studio (Version 1.2.1335).

### GR scaling from birth to maturity in ungulates (MBMGRB-M)

We analysed the scaling of the mean specific body mass GR from birth to maturity (grams/day) with respect to adult body mass in a large sample (107) of species of ungulates including *P. falconeri* using the PanTheria database^33^. GR birth-maturity is computed as adult body mass—neonatal body mass/days (g/day). This approach allows a detailed comparison of the total pre-sexual maturity GR in a large array of ungulate taxa. We have updated some data, especially body mass and ASM of extant elephants based on more recent revisions for ASM in *E. maximus*^30^ and *L. africana*^83,85^. We added a few taxa not previously included by the original authors, using individual body masses from Oboussier’s database (Unpublished data field book H. Oboussier, collections of the Zoological Institute and Museum, University Hamburg, Germany). Ontogenetic body mass data for *P. falconeri* have been inferred on the basis of TDL equations, applied to our sample of 29 aged tibiae (Fig. 1b, c; Supplementary material 4).

### Skeletal maturity (timing of epiphyseal fusion in tibiae)

The sample of 29 tibiae of *P. falconeri*, with their age estimated from histological thin sections, allows inferring the timing of the epiphyses/diaphysis fusion until the age of 23 years (Supplementary material 6, Supplementary Fig. 6) Data on the age of tibae epiphyseal fusion of extant elephants and the fossil taxa *P. antiquus* and *M. primigenius* follow a similar pattern^50,51^.

### *P. falconeri* lifespan inference

We used two variables, tusk length and tusk GR, to calculate the minimum lifespan of *P. falconeri*. Tusk GRs in *L. africana* vary during ontogeny, being considerably higher in young individuals and decreasing rapidly towards senescence (Fig. 4b; Supplementary material 5). Therefore, we selected *P. falconeri* tusk specimens of different sizes and ontogenetic ages to obtain a wide spectrum of the potential GRs during ontogeny. We analysed a total number of 8 tusks from University of Catania Museum (CAT-5; CAT-24; CAT-51; CAT-100; CAT-102; CAT-77-76-78, CAT-94, CAT-37) using both, non-destructive methods (microCT -scans) and destructive methods (sagittal histological thin sections). In two of the eight specimens, CAT-94, CAT-37, however, the poor preservation of the dentine hampered calculation of reliable daily dentine secretion rates (DSR) or dentine extension rates (DER); only six tusks, hence, allowed visualisation of dentine increments (CAT-5; CAT-24; CAT-51; CAT-100; CAT-102; CAT-77-76-78) (Fig. 4a; Supplementary material 5, Supplementary Tables 5, 6).

Tusk length is proportionate to age, and thus maximum lifespan is best estimated from the longest tusks. Unfortunately, the longest Spinagallo tusk^61^ could not be found in the collections of either Catania or Rome, and its current whereabouts are unknown. The second-longest tusks (665 mm measured along the lower edge) from Spinagallo forms part of a mounted skeleton in the public exhibition and is not available for research. We therefore measured the DER and its range of ontogenetic variation in a sample of *P. falconeri* tusks or tusk fragments and combined this with the published length of the longest Spinagallo tusk^61^ (710 mm along inferior edge) to make a rough estimate of *P. falconeri’s* lifespan. Generic extension rate is habitually calculated using other methods than dentine histology (14^C^^38^, growth marks^86,87^). Tusk dentine grows by accretion in a hierarchic system of periodic growth lines following three different temporal arrangements: First-order increments (FOI) are annual; second-order increments (SOI) do not represent a clear temporal period and range from around weekly to fortnightly (two weeks) in continental elephants^86^; third-order increments (TOI) are daily^87^. The sample of Spinagallo tusks shows all three increments. We calculated TOIs from daily dentine secretion rates (DSR µm/day) and measured daily extension rates (DER µm/day); we also used FOIs and TOIs when possible^87^. Two different approaches are generally employed to calculate these features. The first one is a destructive method involving the analysis of the dentine increments by means of histological thin sections; the second one involves non-destructive methods (microCT-scans). In this study, we used both histological thin sections and microCT-scans, because third-order (daily) increments are only visible in histological thin sections in *P. falconeri* tusks (Fig. 5; Supplementary material 5).

TOIs: TOIs are needed to calibrate long-period increments FOIs and SOIs, which then can be used to infer GRs and age at death in elephant tusks^87^ (Fig. 5). Once identified as daily and distinguished from sub-daily, we measured both DSR for calibrating distances within SOIs and TOIs, and DER for estimating the time the whole tusk took to form. The process follows the same principles as estimations of CFT or PFT in molars by means of ESR and EER (Supplementary material 5). However, when calculating DER from TOIs (not from FOIs or SOIs), we did not use the dentine-cementum junction (equivalent to the enamel-dentine junction in molars) but instead the central axis because it is straighter and does not show the typical irregularities such as seasonal thickenings or strangulations where GRs are increased or decreased during favourable/unfavourable periods respectively.

SOIs: SOIs are visible in histological thin sections of several tusks. SOIs in *P. falconeri* have a different growth tempo than in continental elephants. The analysis of the number of daily increments within the SOIs of the CAT-100 tusk indicates that they generally have a monthly periodicity, though some are circum-weekly or, to a lesser extent, fortnightly.

FOIs: FOIs in mammalian dentine, annual in nature, are recognisable as dark–light bands^88^. Elephant tusks show such pairs of dark–light dentine bands in longitudinal sections (or concentric annulations in transversal sections) (Fig. 5; Supplementary material 5) forming nested ‘V’s. The open side of these ‘V’s points towards the posterior part reflecting the pulp cavity at the time of tusk formation^87^. FOI increments have been calibrated on several tusks by counting the number of daily (CAT-24) and second-order increments (CAT-100). Usually, FOI extension rates are measured between the tips of annual incremental dentine cones along the axis of the tusk^86,87^. For this study, we wanted to infer minimal lifespan (MLS) based on the longest Spinagallo tusk, from which the only available information is the length measured along its lower edge^61^. However, the length of the tusk varies if measured on the superior, inferior, or central axis and this does affect the length of the increments. Therefore, DER of FOIs was not measured along the central axis as usual but along the lower edge of the tusks when possible. Common problems in reading and counting the annual banding include difficulty in observing FOIs near to the cementum layer and, depending on the state of preservation of the tusk, the variable preservation of density differences in the dentine banding. The poor state of conservation of Spinagallo tusk dentine, which shows diagenetic alterations including cracking, decalcification, and mineral infiltration, altered their physical aspect and hampered the visualization of growth increments by CT-scanning. Fortunately, histological thin sections of these Spinagallo tusks, inspected at high magnification, allowed measuring DSRs and estimating DERs.

X-ray computed microtomography (microCT) was applied at CENIEH (Burgos, Spain) using a high-resolution x-ray tomography scanner microCT model V|Tome|X s 240 (GE Sensing & Inspections Technologies) (CAT-5, CAT-24, CAT-51, CAT-100 and CAT-102) (Fig. 5a). The last two specimens were scanned in two parts at 200 kV and 150 μA obtaining a final voxel size of 24.5 μm. A filter of 0.2 mm of Cu was used and exposition time during x-ray scans was increased up to 1 s per projection to improve final image contrast. The microCT data of the scanned specimens were imported to the software Avizo 7.1 (FEI-VSG company). The microCT data were re-sliced and reoriented to obtain orthogonal digital cuts in each one of the analysed specimens in a similar manner as obtained from histological slides. For this purpose, 3D models of each microCT scan were generated and aligned to finally obtain orthogonal slices. All measurements of annual features were done using Photoshop CS6 version 13.0.6.

### Phylogenetic generalized least square regressions (PGLS)

To compare the different life-history parameters (ASM, MBMGRB-M and longevity) in an allometric context, we performed phylogenetic generalized least square regressions (PGLS) to evaluate potential phylogenetic signals. For phylogenetic generalized least square regression (PGLS), Pagel’s lambda^89^, and Blomberg’s K^90^, we used package phytools v.0.7–90 using 10,000 simulation rounds to compute p^91^. The phylogeny was downloaded from https://vertlife.org/phylosubsets/, and slightly adjusted to incorporate the fossil species that were originally not present. PGLS analyses were performed in R v.4.1.0^92^ (See Supplementary material 8).

## Molar GRs and crown formation time

We prepared 48 histological thin sections of molars/premolars following standard procedures of our ICP laboratory^72,73^ (Figs. 6, 7, Supplementary material 7). Each tooth was embedded in epoxy resin (Araldite 2020) and longitudinally sectioned. Due to the overall size of the molars, the samples had to be cut in smaller blocks using a low-speed diamond saw (IsoMet, Buehler), and mounted on separate slides. The cut surfaces were then polished using a Metaserv®250 (Buehler) and fixed to a frosted glass with ultraviolet-curing adhesive (Loctite 358). Each sample was then cut and ground with a diamond saw (PetroThin, Buehler) up to a thickness of 150 μm and polished again to obtain a final thickness of approximately 120 μm. Finally, the thin sections obtained were immersed in a histological clearing agent (Histo-Clear II) and covered with a slide cover using DPX medium (Scharlau) to improve visualisation of the dental microscopic features.

We used Zeiss Scope.A1 microscope and images were captured with a digital camera mounted on the microscope (AxioCam ICc5). To measure DSRs and EERs, and to calculate PFT, we followed the methods previously applied to hypsodont *Myotragus balearicus*^66^, which are principally the same as those used by other authors^32^ for their analyses of *P. cypriotes* and *Mammuthus columbi*.

In a preliminary study^93^, some samples of upper and lower *P. falconeri* molar plates were used to infer DSR, EER and PFT. The preliminary results provided a first glimpse of these growth parameters in *P. falconeri*. Unfortunately, apart from the DSRs, the record of EERs was not based on a systematic sampling of the complete length of the plates from apex to cervix; even worse, the great majority of the plates used were not fully grown and/or fragments, which makes them unsuitable for PFT inferences. To overcome this problem, we intensively sampled two unworn but completely formed posterior plates (P8 and P9) of a lower M/3 (CAT-114; Figs. 6, 7, Supplementary material 7). The same method was applied to an upper dp4 to include values of EER and PFT from the deciduous dentition. To our knowledge, it is for the first time that the complete record of the growth period and, hence the formation time of identified entire molar plates of an insular dwarf elephant has been sampled and calculated. Incomplete plates from other molars^93^ were also re-analysed to precisely determine EERs and to expand our data including a wider range of teeth. We furthermore recalculated EERs and PFTs of two incomplete plates of an upper molar (M2 or M3) of the putative ancestor of *P. falconeri*, *P. antiquus*, from the middle Pleistocene site of Torralba (Soria, Spain)^93^. These data, together with the published information on *M. columbi* and *P. cypriotes*^32^ allow a direct comparison of molar growth parameters and PFTs between large continental and dwarf insular elephants.

## Supplementary Information

Supplementary Information.

## References

[1] Gould SJ (1977). Ontogeny and Phylogeny.

[2] Palkovacs EP (2003). Explaining adaptive shifts in body size on islands: a life history approach. Oikos.

[3] Berrigan D, Koella JC (1994). The evolution of reaction norms: Simple models for age and size at maturity. J. Evol. Biol..

[4] Stearns SC, Koella JC (1986). The evolution of phenotypic plasticity in life history traits: Predictions of reaction norms for age and size at maturity. Evolution.

[5] Hanken J, Wake DB (1993). Miniturization of body size: Organismal consequences and evolutionary significance. Annu. Rev. Ecol. Syst..

[6] Johnson AA, Shokhirev MN, Shoshitaishvili B (2019). Revamping the evolutionary theories of aging. Ageing Res. Rev..

[7] Dimitriew CM (2011). The evolution of growth trajectories: What limits growth rate?. Biol. Rev..

[8] Stearns SC (1992). The Evolution of Life Histories.

[9] Foster JB (1964). Evolution of mammals on islands. Nature.

[10] Grant PR (1998). Evolution on Islands.

[11] Biddick M, Hendriks A, Burns KC (2019). Plants obey (and disobey) the island rule. PNAS.

[12] Sander, M., Klein, N., Stein, K., & Wings, O. Sauropod bone histology and its implications for sauropod biology in *Biology of the sauropod dinosaurs: Understanding the life of giants*. (eds. Klein, N. *et al.*) 276–302 (Indiana University Press, 2011)

[13] de Buffrénil V, de Ricqlès A, Zylberberg L, Padian K (2021). Vertebrate Skeletal Histology and Paleohistology.

[14] Klein DR (1965). Ecology of deer range in Alsaka. Ecol. Monogr..

[15] Klein DR, Strandgaard H (1972). Factors affecting growth and body size of roe deer. J. Wildl. Managem..

[16] Clutton-Brock TH, Pemberton JM (2004). Soay-Sheep Dynamics and Selection in an Island Population.

[17] Meiri S, Meijaard E, Wich SA, Groves CP, Helgen KM (2008). Mammals of Borneo: Small size on a large island. J. Biogeogr..

[18] Meiri, S., & Raia, P. in Encyclopedia of Islands (eds. Gillespie, R.G. & Clague, D.A.) 235–239 (University of California Press, 2009).

[19] McNab BK (1994). Resource use and the survival of land and freshwater vertebrates on oceanic islands. Am. Nat..

[20] McNab BK (2010). Geographic and temporal correlations of mammalian size reconsidered: A resource rule. Oecologia.

[21] Roth VL (1992). Inferences from allometry and fossils: Dwarfing elephant on islands. Oxford Surv. Evol. Biol..

[22] Ozgul A (2009). The dynamics of phenotypic change and the shrinking sheep of St. Kilda. Science.

[23] Raia P, Barbera C, Conte M (2003). The fast life of a dwarfed giant. Evol. Ecol..

[24] Palombo MR (2007). How can endemic proboscideans help us understand the ‘‘island rule’’? A case study of Mediterranean islands. Quat. Inter..

[25] Larramendi A, Palombo MR (2015). Body size, biology and encephalization quotient of palaeoloxodon ex gr. *P. falconeri* from Spinagallo Cave (Hyblean plateau, Sicily). Hystrix.

[26] Bromage, T. *et al.* in World islands in prehistory: International insular investigations V Deia conference of prehistory (eds. Waldren, W. H. & Ensenyat, J. A.) 420–427 (British Archeol. Rep. Intern. Series, 2002).

[27] Benton MJ (2010). Dinosaurs and the island rule: The dwarfed dinosaurs from Hateg Island. Palaeogeogr. Palaeoclimatol. Palaeoecol..

[28] Diniz-Filho JA (2019). Quantitative genetics of body size evolution on islands: An individual-based simulation approach. Biol. Lett..

[29] Hanks J (1972). Growth of the African elephant (*Loxodonta africana*). E. Afr. Wildl. J..

[30] Lahdenperä M, Mar KU, Lummaa V (2014). Reproductive cessation and post-reproductive lifespan in Asian elephants and pre-industrial humans. Front. Zool..

[31] Larramendi A (2016). Shoulder height, body mass, and shape of proboscideans. Acta Palaeontol. Pol..

[32] Dirks W, Bromage TG, Agenbroad LD (2012). The duration and rate of molar plate formation in *Palaeoloxodon cypriotes* and *Mammuthus columbi* from dental histology. Quat. Int..

[33] Jones KE (2009). PanTHERIA: A species-level database of life history, ecology, and geography of extant and recently extinct mammals. Ecol..

[34] Smuts GL (1975). Reproduction and population characteristics of elephants in Kruger National Park. J. Sth. Afr. Wildl. Mgmt. Assoc..

[35] Marín-Moratalla N, Jordana X, García-Martínez R, Köhler M (2011). Tracing the evolution of fitness components in fossil bovids under different selective regimes. C. R. Palevol.

[36] Whyte IJ, Hall-Martin A (2018). Growth characteristics of tusks of elephants in Kruger National Park. Pachyderm.

[37] Tacutu R, Thornton D, Johnson E, Budovsky A, Barardo D, Craig T, Diana E, Lehmann G, Toren D, Wang J, Fraifeld VE, Magalhães JP (2018). Human ageing genomic resources: New and updated databases. Nucleic Acids Res..

[38] Uno KT (2013). Bomb-curve radiocarbon measurement of recent biologic tissues and applications to wildlife forensics and stable isotope (paleo)ecology. Proc. Natl. Acad. Sci. U.S.A..

[39] Lee PC, Sayialel S, Lindsay WK, Moss CJ (2012). African elephant age determination from teeth: Validation from known individuals. Afr. J. Ecol..

[40] Lee PC, Fishlock V, Webber C, Moss CJ (2016). The reproductive advantages of a long life: longevity and senescence in wild female African elephants. Behav. Ecol. Sociobiol..

[41] Mumby HS (2015). Distinguishing between determinate and indeterminate growth in a long-lived mammal. BMC Evol. Biol..

[42] Stansfield FJ (2015). A novel objective method of estimating the age of mandibles from African elephants (*Loxodonta africana africana*). PLoS ONE.

[43] Crawley JAH (2020). Taming age mortality in semi-captive Asian elephants. Sci. Rep..

[44] El Adli JJ (2015). Natural history museum of Los Angeles County. Sci. Ser..

[45] Fisher *et al.* Five Years in the Life of an Aucilla River Mastodon, 12. In *First Floridians and Last Mastodons: The Page-Ladson Site in the Aucilla River*, Webb, S.D. (ed.), 343–377 (2006).

[46] Grigoriev SE (2017). A woolly mammoth (Mammuthus primigenius) carcass from Maly Lyakhovsky Island (New Siberian Islands, Russian Federation). Quat. Inter..

[47] Mayne B, Berry O, Davies C, Farley J, Jarman S (2019). A genomic predictor of lifespan in vertebrates. Sci. Rep..

[48] Promislow DEL, Harvey PH (1990). Living fast and dying young: A comparative analysis of life-history variation among mammals. J. Zool. Lond..

[49] Stearns SC (2000). Life history evolution: Successes, limitations, and prospects. Naturwiss.

[50] Herridge, V. L. Dwarf elephants on Mediterranean islands: a natural experiment in parallel evolution. PhD thesis, University College London (2010).

[51] Lister, A. M. Epiphyseal fusion and postcranial age determination in the woolly mammoth *Mammuthus primigenius*. In: *Mammoths and the Mammoth Fauna: Studies of an Extinct Ecosystem* (DEINSEA 6, Ed., 1999).

[52] de Magalhães JP, Costa J, Church GM (2007). An analysis of the relationship between metabolism, developmental schedules, and longevity using phylogenetic independent contrasts. J. Gerontol. Biol. Sci. Med. Sci..

[53] Kirkwood TBL, Rose MR (1991). Evolution of senescence: Late survival sacrificed for reproduction. Phil. Trans. R. Soc. Lond. B.

[54] Jones OR (2008). Senescence rates are determined by ranking on the fast–slow life-history continuum. Ecol. Lett..

[55] Lemaitre J-F (2015). Early-late life trade-offs and the evolution of ageing in the wild. Proc. R. Soc. B.

[56] Sandvig EM, Coulson T, Clegg SM (2019). The effect of insularity on avian growth rates and implications for insular body size evolution. Proc. R. Soc. B.

[57] Alcover JA, McMinn M (1994). Predators of Vertebrates on Islands. BioSci.

[58] Medawar PB (1952). An Unsolved Problem of Biology.

[59] Williams GC (1957). Pleiotropy, natural selection, and the evolution of senescence. Evol..

[60] Kirkwood TBL (2008). Understanding ageing from an evolutionary perspective. J. Internal Med..

[61] Ambrosetti P (1968). The Pleistocene dwarf elephants of Spinagallo (Siracusa, South-eastern Sicily). Geol. Romana..

[62] Voorhies MR (1969). Taphonomy and population dynamics on an early Pliocene vertebrate fauna, Knox County. Nebraska. Univ. Wyoming Contrib. Geol..

[63] Bielby J (2007). The fast-slow continuum in mammalian life history: An empirical reevaluation. Am. Nat..

[64] Köhler M, Moyà-Solà S (2009). Physiological and life history strategies of a fossil large mammal in a resource-limited environment. Proc. Nat. Acad. Sc..

[65] Köhler, M. Fast or slow? The evolution of life history traits associated with insular dwarfing. In: *Islands and Evolution*. (eds Perez-Mellado, V. & Ramon, C.) Institut Menorquí d'Estudis. Recerca, **19** 261–280 (2010).

[66] Jordana X, Köhler M (2011). Enamel microstructure in the fossil bovid *Myotragus balearicus* (Majorca, Spain): Implications for life history evolution of dwarf mammals in insular ecosystems. Palaeogeogr. Palaeoclimatol. Palaeoecol..

[67] Long ES, Courtney KL, Lippert JC, Wall-Scheffler CM (2019). Reduced body size of insular black-tailed deer is caused by slowed development. Oecologia.

[68] Kolb C (2015). Growth in fossil and extant deer and implications for body size and life history evolution. BMC Evol. Biol..

[69] Hayashi, S. *et al.* Variation and mechanisms of life history evolution in insular dwarfism as revealed by a natural experiment. bioRxiv (2020).

[70] Rozzi R, Lomolino MV (2017). Rapid dwarfing of an insular mammal: The feral cattle of Amsterdam Island. Sci. Rep..

[71] Berteaux D, Micol T (1991). Population studies and reproduction of the feral cattle (Bos taurus) of Amsterdam Island Indian Ocean. J. Zool. Lond..

[72] Nacarino-Meneses C, Köhler M (2018). Limb bone histology records birth in mammals. PLoS ONE.

[73] Orlandi-Oliveras G, Nacarino-Meneses C, Koufos GD (2018). Bone histology provides insights into the life history mechanisms underlying dwarfing in hipparionins. Sci. Rep..

[74] Werner J, Griebeler EM (2014). Allometries of maximum growth rate versus body mass at maximum growth indicate that non-avian dinosaurs had growth rates typical of fast growing ectothermic sauropsids. PLoS ONE.

[75] Griebeler EM (2013). Body temperatures in dinosaurs: What can growth curves tell us?. PLoS ONE.

[76] Erickson GM, Rogers KC, Yerby SA (2001). Dinosaurian growth patterns and rapid avian growth rates. Nature.

[77] Quince C, Abrams PA, Shuter BJ, Lester NP (2008). Biphasic growth in fish I: Theoretical foundations. J. Th. Biol..

[78] Quince C, Shuter BJ, Abrams PA, Lester NP (2008). Biphasic growth in fish II: Empirical assessment. J. Th. Biol..

[79] Boukal DS, Dieckmann U, Enberg K, Heino M, Jørgensen C (2014). Life-history implications of the allometric scaling of growth. J. Theor. Biol..

[80] Fitzhugh HA (1976). Analysis of growth curves and strategies for altering their shape. J. Anim. Sci..

[81] Toms JD, Lesperance ML (2003). Piecewise regression: A tool for identifying ecological thresholds. Ecology.

[82] Segura AM, Milessi AC, Vögler R, Galván-Magaña F, Muggeo V (2013). The determination of maturity stages in male Elasmobranchs (Chondrichthyes) using a segmented regression of clasper length on total length. Can. J. Fish. Aquat. Sci..

[83] Freeman EW, Whyte I, Brown JL (2008). Reproductive evaluation of elephants culled in Kruger National Park, South Africa between 1975 and 1995. Afr. J. Ecol..

[84] Moss CJ (2001). The demography of an African elephant (Loxodonta africana) population in Amboseli, Kenya. J. Zool. Lond..

[85] Moss, C. J. & Lee, C. J. *The Amboseli Elephants: A long-term perspective on a long-lived mammal* (University of Chicago Press, 2011).

[86] Fisher, D. C. In: *Proc. of the International Conference on Mammoth Site Studies*. (ed. West, D.) 121–135 (Publications in Anthropology 22. University of Kansas, Lawrence, 2001).

[87] Rountrey, A. N. Life Histories of Juvenile Woolly Mammoths from Siberia: Stable Isotope and Elemental Analyses of Tooth Dentin. PhD thesis, The University of Michigan (2009).

[88] Fisher, D. C. In *The Proboscidea: Evolution and Paleoecology of Elephants and Their Relatives*. (eds. Shoshani, J. & Tassy, P.) 296–315 (Oxford University Press, 1996).

[89] Pagel M (1999). Inferring the historical patterns of biological evolution. Nature.

[90] Blomberg SP, Garland T, Ives AR (2003). Testing for phylogenetic signal in comparative data: Behavioral traits are more labile. Evolution.

[91] Revell LJ (2012). phytools: An R package for phylogenetic comparative biology (and other things). Methods Ecol. Evol..

[92] R Core Team R: A Language and Environment for Statistical Computing. Vienna: R. (2019).

[93] Jordana, X. *et al*. 3rd International Symposium on Paleohistology. (eds. Canoville, et al.) 88 (University of Bonn, 2015).

