# Supplementary material for: Palaeohistology reveals a slow pace of life for the dwarfed Sicilian elephant
Source: Sci Rep. 2021 Nov 24;11:22862. doi: 10.1038/s41598-021-02192-4 (PMC8613187; doi:10.1038/s41598-021-02192-4)
Supplement: Supplementary file 1 — Supplementary Information. [file 41598_2021_2192_MOESM1_ESM.pdf]

Palaeohistology reveals a slow pace of life for the dwarfed Sicilian elephant.

Meike Köhler [1,2*], Victoria Herridge[3], Carmen Nacarino-Meneses[1,4], Josep Fortuny[1], Blanca Moncunill-Solé[5,6] Antonietta Rosso[7], Rossana Sanfilippo[7], Maria Rita Palombo[8], Salvador Moyà-Solà[1,2].

1 Institut Català de Paleontologia Miquel Crusafont, Universitat Autònoma de Barcelona. Cerdanyola del Vallès, Barcelona, Spain; 2 Institució Catalana de Recerca i Estudis Avançats (ICREA). Barcelona, Spain; 3 Earth Sciences, Natural History Museum, Cromwell Road, London, UK; 4 Department of Biological Sciences, University of Cape Town. Rondebosch, Cape Town, South Africa; 5 Dipartimento di Scienze, Università degli Studi Roma Tre. Roma, Italy. 6 Centro de Investigacións Científicas Avanzadas, Universidade da Coruña. A Coruña, Spain; 7 Dipartimento di Scienze Biologiche, Geologiche e Ambientali, Università di Catania, Catania, Italy; 8IGAG-CNR, c7o Earth Science Department, Sapienza University of Rome, Italy. *Corresponding author.

Supplementary material:

1. Geological and chronological context.

2. Body mass of *P. falconeri* and extant elephants.

3. Tibia histology and ontogeny.

4. Growth rate.

5. Tusk growth rate and lifespan inference.

6. Age at sexual maturity in *P. falconeri*.

7. Molar growth rates and crown formation time of *P. falconeri* cheek teeth

8. Phylogenetic generalized least square regressions (PGLS).

9. The Spinagallo mortality profile.

10. List of acronyms

11. References

1. *P. falconeri*, geological and chronological context.

The Spinagallo Cave faunal assemblage, which yielded the richest sample of *P. falconeri*, has been regarded as the epitome of the "*Elephas*" *falconeri* Faunal Complex, the oldest, and most endemic, of Sicily's Pleistocene fauna units (Belluomini and Bada, 1985). The Spinagallo fauna has been believed for decades to be early Middle Pleistocene in age, based on an Amino Acid Racemization (AAR) date of ca. 500 ka (Belluomini and Bada, 1985). This age must be considered with high caution, not only because of the uncertainties connected with the method, but also because the sample used for calibration of the isoleucine epimerization rate was that from the Isernia la Pineta deposit (Upper Volturno Basin), at the time dated at~700 ka (Delitala et al.,1983), while new 40Ar/39Ar radiometric dating demonstrated that it is substantially younger (~ 600 ka) (Coltorti et al., 2005). New dates [U-Th & Optically Stimulated Luminescence (OSL)] for the fossil elephant material at Spinagallo Cave suggest an age of deposition between 367 and 233

ka (Herridge et al., 2014, Richards et al., 2019). The putative mainland ancestor of the Sicilian and Malta endemic lineages, *P. antiquus*, is first recorded in NE Italy at Slivia by the end of the Early Pleistocene, and in central (Isernia La Pineta) and Southern Italy (Notarchirico, Basilicata) about 600 ka. Therefore, the straight tusked elephants may have reached Sicily at any time after this date, with the most suitable palaeogeographic conditions for colonisation during MIS 12 (at about 478 ka,).

This provides a 'window' of interest (ca 600 ka to ca 300k, although note the first appearance date for *P. antiquus* in Europe is 780ka) for understanding the contemporary geographic setting for the evolution of *P. falconeri*, setting – relevant for considering the importance of resource limitation.

2. Body mass of *P. falconeri* and of extant elephants

Body mass of P. falconeri. Amongst insular dwarf elephants, BM estimations from different skeletal parameters follow broadly similar patterns across all Mediterranean taxa, indicating isometric scaling trends. Nevertheless, equations based on intraspecific allometries of continental *Loxodonta africana* and *Elephas maximus* provide higher BM estimates from widths and circumferences than from lengths of long bone shafts (Herridge, 2010). These incongruences are due to the fact that the body plan of insular dwarf elephants differs substantially from that of continental elephants used as models for body mass inferences (Herridge, 2010), which importantly distorts body mass estimations. Thus, equations based on intraspecific allometries of extant elephants (skeletal dimensions and shoulder high), led to BM estimations between 58 and 1942 kg for *P. falconeri* (Roth, 1990; Christiansen, 2004; Palombo and Giovinazzo 2005) depending on the osteological parameter and the predictive equation used (Herridge, 2010). Furthermore, it is problematic to extrapolate mass-estimation equations beyond their model-sample size range. This is the case when applying data of extant (continental large) elephants to infer body masses of dwarf elephants. Therefore, we discarded the use of equations based on intraspecific allometries of extant elephants to infer BM in *P. falconeri*. 3D volumetric Reconstruction (Larramendi and Palombo, 2015; Romano et al., 2019) is an independent method based BM inferences from reconstructed body volumes in 3 dimensions, digitally sculpted on photogrammetric models of mounted skeletons. This method is especially relevant and useful in the case of endemic insular taxa, because it considers the specific adaptations and anatomical modifications selected under insularity. Two independent studies (Larramendi and Palombo, 2015; Romano et al., 2019) based on the same mounted skeletons of different sex and age of *P. falconeri*, provided very similar results, suggesting that these BM estimates are more accurate than those based on intraspecific scaling. However, this method can only be used with complete, mounted skeletons and is not applicable to the whole sample of isolated bones of Spinagallo; it, thus, does neither describe the degree of sexual size dimorphism, nor the size variability or the ontogenetic growth stages.

Considering that equations based on extant elephant intraspecific scaling do not provide accurate estimates and that 3D volumetric reconstructions cannot be applied to the sample of isolated bones from Spinagallo, we used a database of skeletal data and body masses of extant mammals (Christiansen, 1999) to infer realistic mean body masses from isolated bones for the entire population (Supplementary table 1, 2). Christiansen's database (Christiansen, 1999) includes the minimal shaft circumference of tibia and body mass of a sample of 98 specimens of extant terrestrial mammals, representing 79 species from seven orders. The database is divided into various sub-sets; we used the one that includes all mammals and another that includes mammals over 50 kg of body weight. The BM estimates obtained (Supplementary table 1, 2) with this method provided very similar results to those found by 3D volumetric reconstruction (Supplementary table 3) (Larramendi and Palombo, 2015; Romano et al., 2019; Christiansen, 1999; Anderson et al., 1985; Campione and Evans, 2012) which validates this approach. We used these results for comparative purposes to contextualise the life-history events. The mean BM value (males and females) used for comparative analysis is 242,38 kg (Supplementary table 3).

Body mass of extant elephants. In the present work, we used the body mass of extant elephant taxa, *Loxodonta africana* and *Elephas maximus*, for comparative purposes. The databases here employed to analyze the scaling of several life-history traits (Jones et al., 2009; Werner and Griebeler, 2014; Tacutu et al., 2018) contain extant elephant BM values that do not include the most recent information and revisions on elephant body mass. However, the latest review (Larramendi, 2016) provides a critical updating of the body mass of extant elephants. After a rigorous discussion, this author proposes a mean BM value for male and females *L. africana* (4.000.000 – 2.700.000 gr respectively; mean value 3.350.000 gr) and for *E. maximus* (6.000.000 – 3.000.000 gr. respectively; mean value 4.500.000 gr). Finally, the body mass of newborn elephants was obtained from Dale (2010), suggesting for *L. africana* a mean value of 103.550 gr. and for *E. maximus* a mean value of 117.900 gr. We included these data in the general databases here used.

TLC	Nº	Min	Mean	Max	SD
**AME**					
**Female**	26	111,4	158,47	238,98	29,75
**Male**	10	247,26	325,96	424,01	65,43
**LME**					
**Female**	26	111,4	153,36	238,98	30,88
**Male**	10	246,37	331,41	438,33	71,02

Supplementary table 1. Body mass estimates of *P. falconeri* based on tibia least circumference (TLC) of adult individuals (fused epiphysis) from Spinagallo using the equations based on the tibia TLC (Christiansen, 1999). All mammal equation AME (Least squares; a= 13,48; b= 0,36; n= 80; 95% CI= 0,014; R^2= 0,98). Large mammal equation LME (Least squares; a= 15,32; b= 0,39; n= 36; 95% CI= 0,040; R^2= 0,95).

TIBIA LCT	n	Mean	SD	SE	95% CI	Range	
Female	26	153,36	30,89	6,06	140,88-165,84	111,4	238,99
Male	10	331,41	71,03	22,46	280,60-382,22	246,37	438,33

Supplementary table 2. Basic statistics of the BM inference of *P. falconeri* on the basis of the tibia LCT.

Body mass	Male	Female	Juvenile male	New born male
Tibia LTC / AME Christiansen, (1999)	327,0	158,5		13,4*
Tibia LTC / LME Christiansen, (1999)	331,4	153,4		11,0*
Larramendi et al. (2015)	304,5	167,3		6,7
Romano et al. (2019)	249,9	150,5	38,8	7,8
Anderson et al., (1955)	342,1	161,8	51,0	14,0
Campione & Evans (2012)	401,2	188,5	58,9	15,9

Supplementary table 3. Comparisons of the mean body mass estimates of *P. falconeri* from the tibia least circumference (TLC) (Christiansen, 1999). with BM estimates from other authors using different methods or predictive equations: body mass inferred with 3D volumetric reconstructions of mounted skeletons (Larramendi and Palombo, 2015; Romano et al. 2015), and results based on other regressions (Anderson et al. 1985, Campione and Evans, 2012). AEM: All mammal equation and LEM: Large mammal equation (Christiansen, 1999). *Smallest tibia from the Spinagallo cave sample. Body mass in kg.

3. Tibia histology and ontogeny.

Inferring the growth rate (GR) and the age at sexual maturity (SM) of *P. falconeri* on the basis of the growth of the postcranial skeleton is the basic objective of this and the two subsequent sections. The reliability of the results depends on sample quality and quantity of the histological growth record of bones (and teeth), as well as on the selection of the empirical method or growth models capable to identify from this record the specific time when reproductive maturity (or other life-history events) took place. It is rare that bones show unambiguous indicators of sexual maturity (as is the case of medullary bone in birds and bird-like dinosaurs, an exception in vertebrates (Lee and Werning, 2008; Erickson et al., 2001; Griebeler and Werner, 2018). This goal is further complicated when the results obtained from fossil taxa are compared with extant species. In extant taxa, data on sexual maturity usually come from field observations, a very different method from those applied to fossil taxa. Therefore, we take special care in establishing the necessary methodological and conceptual protocols to compare homologous life-history events between fossil and extant taxa. In female *Loxodonta* tibial shaft length and shoulder height approach asymptote shortly after 10 years of age, the time close to which they also

begin to become sexually mature (Moss 2001, 2010; Herridge, 2010), with externally visible evidence of epiphyseal fusion from 15 years. We therefore chose the tibia for our analyses as its growth straddles the period of sexual maturation. We made histological thin sections from a large sample of all ontogenetic growth stages of *P. falconeri* for skeletochronology and used diaphyseal lengths (TDL) to infer age and size at sexual and skeletal maturity. Sexual and skeletal maturity normally do not coincide in mammals, which attain sexual maturity earlier than bone growth halts and before fusion of all epiphyses is completed (Padian et al., 2013). The selected sample consists of 24 right and 6 left tibiae, mainly of young individuals without epiphyses. We also included some almost mature individuals (with partially fused epiphyses) that we sexed by size considering that females are clearly smaller than males (Herridge, 2010; Raia, 2003; Larramendi and Palombo, 2015; Romano et al., 2019). With the selected sample, ageing was possible until 23 years.

Aging the tibiae: skeletochronology. We aged a sample of 24 right and 5 left tibiae of *P. falconeri* from Spinagallo site, representing an ontogenetic series from neonates to adults, by skeletochronology show examples for all ontogenetic stages (Figure 1a, 2; Supplementary table 4). The three smallest tibiae, T106 (IPS84887), T108 (IPS84888) and T109 (IPS84889) belong to newborn elephants and show either no or an only faint neonatal line but almost no newly deposited bone after birth, indicating that the individuals did not survive for more than one day after birth at most. 4 individuals are roughly one year old: ELEPH1-T IPS83761, T119 IPS84890, T127 IPS96267 and T27 IPS107629. They show a clear transition from prenatal (fibro-lamellar cortex with a high percentage of parallel-fibred bone) to postnatal tissue (longitudinal and radial-oriented fibro-lamellar bone). This 1st - year tissue is easy to recognize by its radial growth even in older individuals until around the age of 7 years, before Haversian systems erase large parts of the primary tissue. Until the age of 7 years, all specimens show clear LAGs within the zones that are invaded by Haversian systems. Between 8 and 12 years, Haversian systems become increasingly denser. For T3 (IPS83763) we counted 12 principal LAGs, which tended to appear in bundles associated with approximately 3 secondary LAGs from 9 years onwards. At this stage individuals are still vigorously growing as show the open blood vessels at the outer cortical surface (T45, IPS96270). External fundamental systems form from 14 years onwards (Roma6 IPS70096_c, T203 IPS- IPS96273), with countable LAGs especially along the anterior and / or internal crests where bone apposition continued over part of the year and, hence, resolution is best (Roma6 IPS70096, T203 IPS- IPS96273) (Supplementary figure 1).

Catalog nº	MA	TDL	TLC	DAPMS	PEF	DEF	EFS	SIDE	OB
T106	0	52,90	34,34	10,62	NO-EP	NO-EP	NO	R	
T108	0	53,70	40,13	11,53	NO-EP	NO-EP	NO	R	
T109	0	56,88	36,81	11,85	NO-EP	NO-EP	NO	R	
ELEPH1-T	1	63,00	40,98	12,39	NO-EP	NO-EP	NO	R	
T119	1	64,07	38,44	12,13	NO-EP	NO-EP	NO	R	
T127	1	71,80	43,70	14,15	NO-EP	NO-EP	NO	R	
T27	1	73,19	48,94	12,96	NO-EP	NO-EP	NO	R	
T124	2	69,29	41,32	13,00	NO-EP	NO-EP	NO	R	
T36	2	84,02	50,24	16,30	NO-EP	NO-EP	NO	R	
T138	2,5	86,50	50,63	16,44	NO-EP	NO-EP	NO	R	
T135	3	81,42	55,51	16,60	NO-EP	NO-EP	NO	R	
T136	3	81,66	50,77	14,94	NO-EP	NO-EP	NO	R	
T128	3,5	74,62	50,65	16,27	NO-EP	NO-EP	NO	R	
ELEPH2-T	4	93,00	60,45	16,50	NO-EP	NO-EP	NO	L	
T132	4	74,40	58,99	15,40	NO-EP	NO-EP	NO	R	
T133	4	79,50	59,62	19,00	NO-EP	NO-EP	NO	R	
T35	5	80,30	57,51	17,54	NO-EP	NO-EP	NO	R	
T140	7	97,76	65,14	19,60	NO-EP	NO-EP	NO	R	1
T139	7	92,40	70,21	20,19	NO-EP	NO-EP	NO	R	2
T42	9	109,50	69,50	21,80	NO-EP	NO-EP	NO	R	3
T45	9	112,98	73,97	23,00	NO-EP	NO-EP	NO	L	4
T142	11	139,50	74,62	24,30	NO-EP	NO-EP	NO	R	5
ELEPH3-T	12	125,00	79,17	24,00	NO-EP	NO-EP	NO	L	6
T46	13	132,70	77,98	24,11	NO-EP	NO-EP	NO/YES	L	7
T179	14	115,50	75,57	25,12	EP-SV	NO-EP	NO/YES	R	8
T181	18		82,23	26,99	EP-SV	?	YES	R	9
T ?	22	158,00	91,27	29,00	NO-EP	NO-EP	NO/YES	R	10
T203	22		85,31	28,20	?	EP-SV	YES	R	11
Roma 6	23	149,60	86,50	29,10	EP-SV	EP-SV	YES	L	12

Supplementary table 4. List of tibia specimens used in this work. The first column indicates the catalogue number of the Museum of Earth Sciences from the Catania University and Earth Sciences Museum (Rome University); ME= minimal age calculated using the number of lags; TDL= tibia diaphysis length; TLC= least diameter of the mid-shaft of the tibia; PEF: proximal epiphysis fusion; DEF= distal epiphysis fusion; EFS= presence / absence of external fundamental system; Side: R= right, L=left. OB= Observations based on histological sections: 1: appositional growth still vigorous; 2: appositional growth still vigorous, some HS in the outer cortex; 3: still growing with open blood vessels, too bad preserved as to show the presence of HSs (HSs= Haversian system); 4: growth still vigorous, no HSs in the outer cortex; 5: growth still vigorous, no HSs in the outer cortex; 6: growth still vigorous, a few HSs in the outer cortex; 7: slow growth, HSs invading outer cortex, some large resorption cavities in parts of the outer cortex, #84896-1 seems to show locally an EFS; 8: slow growth (dense disposition of LAGs) with some few blood vessels and little if any HSs in the outermost cortex, suggests that EFS is about to be deposited; 9: partially still slow growth, partially EFS, few HSs; 10: partially lamellar bone with densely spaced LAGs (partial EFS), resorption cavities and HSs within OCL, some active blood vessels within OCL; 11: clear EFS, only a few HSs in the outer cortex, strand line through local resorption of the outer cortex; 12: almost completely filled with HSs throughout, difficult to see primary bone, EFS only locally with countable LAGs. (T? indicates the absence of collection number).

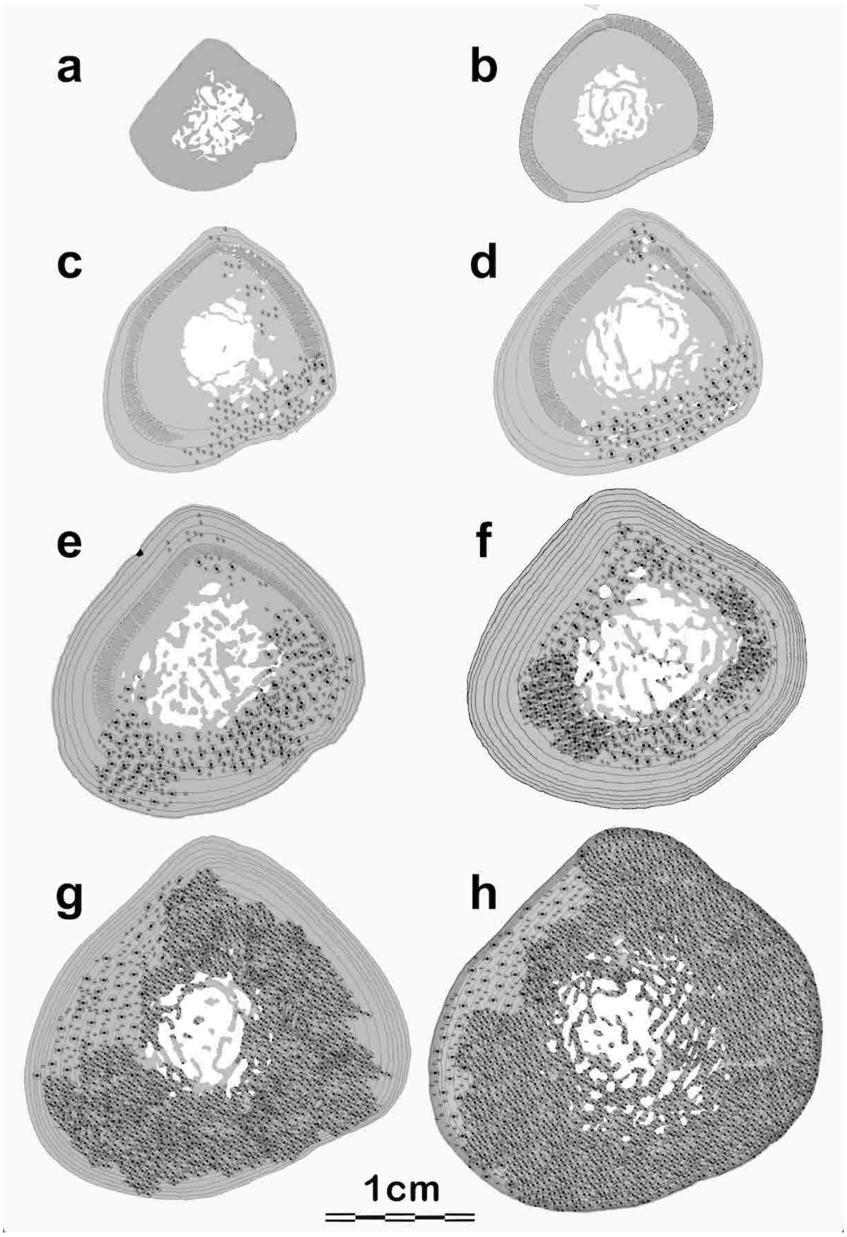

Supplementary figure 1. Schematic representation of tissue types in histological sections of an ontogenetic series of tibiae showing 1st year's radial growth (b-e), expansion of Haversian systems (c-h), and external fundamental system (closely-packed LAGs in h): a: neonate (T106, IPS84887); b: age ≈ 1y (ELEPH1-T, IPS83761); c: age 2,5y (T138, IPS84893); d: age 5y (T35, IPS107631); e: age 7y (T139, IPS-104359); f: age 11y (T142, IPS96266); g: age 12y (T3, IPS83763); h: age 23y (Roma6, IPS70096). Anterior crest points up. Graphic scale 1 cm. Drawing M.K.

4. Growth rate.

Scaling of the mean body mass growth rate from birth to maturity (*MBMGRB-M*). It is well known that extant elephants have a low growth rate compared with other mammals (Mumby et al., 2015; Hanks, 1972). The scaling of the mean body mass growth rate from birth to maturity (MBMGR, expressed in grams/day) in respect to adult body mass, using phylogenetic generalized least square regressions (PGLS), in a large sample (n=107) of species of extant ungulate mammals and elephants (Data from PanTheria, Jones et al., 2009) (see Supplementary material 8), provides a way to directly compare MBMGRB-M across ungulate taxa. In an allometric context, the analysis shows that *P. falconeri* has the lowest values of MBMGRB-M ever recorded for any ungulate species, including extant elephants (Figure1 c, d). The scaling exponent of MBMGRB-M and adult body mass indicates negative allometry; it implies that large taxa have lower MBMGRB-M, in other words that MBMGRB-M decreases with size. However, some taxa, as for example *Naemorhaedus goral*, *Hemitraus jemlahicus, Camelus dromedarius* and other taxa from ecological settings with low predation pressure are similar to extant elephants in that they show lower residuals. The very low values of *P. falconeri* surpass the limits of any extant ungulate, being in this respect an exceptional case. Distribution of the residuals of the PGLS log10MBMGRB-M (adult-neonatal body mass, gr/age from birth, days) (y) against log10 adult body mass (x) clearly shows that *P. falconeri* has the lowest MBMGRB-M values compared with extant ungulate, including elephants (Figure1 c, d).

5. *P. falconeri* tusk growth rate and lifespan inference.

The purpose of this section is to test whether *P. falconeri* had a short lifespan compared with extant elephants downscaled to a small size, as some authors suggested (Raia, 2003; Laramendi and Palomo, 2015; Bromage et al., 2002; Palombo, 2007) or if instead it had a longer lifespan than expected from scaling relationships, as predict life history models for evolution in insular environments (Stearns, 1999; Palkovacs, 2003). Under the first assumption, the scaling of longevity with body mass (BM) in extant ungulates, *P. falconeri* should have had a lifespan between 27 and 36 years (using mean values for pooled males and females) (Raia, 2003; Laramendi and Palomo, 2015). The objective of this section, hence, is to test this prediction using tusk histology in *P. falconeri*. Analysis of growth marks in dentine can provide absolute age estimates (Klevezal et al., 1998) and its application to structures that grow over the entire lifespan of the organism is the best method to infer longevity. The tusks of elephantids grow throughout life thanks to their open root that does not occlude with age. Calculating the repeat interval of incremental features in the dentine of life-long growing tusks, the tusk length allows calculating individual lifespan and age at death (Whyte and Hall-Martin, 2018). The lifespan inferred by this method is a minimal lifespan, considering that tusks are subject to apical wear during life (Whyte and Hall-Martin, 2018; Ambrosetti, 1968).

Tusk analysis.

CAT-5. This is the smallest specimen of the sample (Figure 4a, 5, Supplementary table 5). It is complete, showing wear in its anterior part; the missing part is probably small. In spite of its small size, it is the definitive (secondary) tusk. In this specimen, we measured daily secretion rate (DSR) and dentine extension rate (DER). DSR and DER have been measured at different points of the tusks, providing a mean value of 12.57 mm/y measured along the axis of the tusk (Supplementary table 6). Considering that this tusk is straight, the lower and the upper edges have the same length, so that the DER values are similar at both edges and at the central axis. For these reasons, the mean extension rate of this tusk used is the mean value of the DER daily increments measured along the central axis (12.57 mm/y). On its basis, the age of the tusk is 4.05±0.74 years along its preserved length (Supplementary table 6).

CAT-24. This is the second smallest tusk of the analysed sample (Figure 4a, 5, Supplementary table 5). This specimen was scanned and histological thin sections were obtained (Figure 5). DDSR and DER were measured on the slides. Calculation of the number of days between incremental lines indicates the annual nature in seven of them – called First Order Increments FOIs, with a mean number of 351 daily increments (Figure 5b). DER has been calculated along the central axis providing a mean value of 8.86±2,29 (mm/y); first order increments on the lower edge of the tusk provide a longer estimation, consequence of the strong curvature of the tusk, with a mean value of 10,34±1,73 (mm/y). We inferred an age of 10.95±0.93 years for length of the tusk (Supplementary table 6).

CAT-51. This specimen is a partial tusk, relatively thin and lacking its anterior part (Figure 4a, 5, Supplementary Table 5). The specimen was scanned, sectioned along the sagittal plane and histological thin sections were obtained. The distal portion of the pulp cavity is preserved in the proximal part of the tusk. Probable first order and second order increments are visible on the sagittal section. However, the bad preservation of the dentine, showing a large number of secondary cracks, hampers the accurate counting of the first order increments and second order increments in this tusk, discouraging the use of these structures for inferences of age at death of this specimen. Furthermore, the second-order increments could not be calibrated in this tusk by means of daily increments. DER was therefore based on daily increments only. The mean value of DER is 10.56±1.71, providing a minimal age of 12 years for this tusk (Supplementary table 5).

CAT-100. This specimen (Figure 4a, 5, Supplementary table 5) was scanned, the anterior half was sectioned and histological thin sections were obtained. First-order increments are weak in this tusk; however, second-order increments are clearly visible in the CT-scans (monthly SOIs, Figure 5d) and in histological thin sections (monthly SOIs: Figure 5d; circum-monthly and fortnightly SOI). Considering that first and second-order increments may be recognised by counting included third-order features (El Adli, 2018; Fisher and Fox, 2006), we have calculated the number of daily increments within second-order increments of this tusk. The biological rhythmic cycles of organisms have different

duration. One of them is the monthly rhythm, which length depends on the calendar utilised. The seven second-order increments measured in the CAT- 100 tusk have a mean value of 29.54 days thus fitting the length of the synodic month, the complete cycle of phases of the moon as seen from Earth, which averages 29.530588 mean solar days in length (Supplementary table 7). Changes in nighttime light intensity that depend on the monthly changes in the position of the moon in relation to earth and sun, alter physiological processes such as heart rate and body temperature associated with modifications in activity even in diurnal animals (Portugal et al., 2019). Changes in heart rate and body temperature, in turn, impact bone apposition rate Köhler et al. in prep.), which might explain the monthly synodic cycles seen in the tusks of Sicilian elephants. Using second-order monthly groups of 12, first-order increments have been identified and counted. Annual extension rates based on daily increments along the inferior tusk surface show a mean value of 10.23±2.17 mm; FOIs, based on 12 monthly SOIs result in an annual extension rate of 10.32±1.96 mm. Thus, the total length of CAT-100, measured along the inferior tusk surface, provides an estimated age of 23,95 and 23,72 years, respectively (Supplementary table 6).

CAT-102. This specimen (Figure 4a, 5, Supplementary Table 5) was only scanned; it has not been cut for thin sections. First-order increments are visible, but SOIs are not visible in this specimen. FOIs are clearly visible as pairs of bands of dark-light dentine on longitudinal sections or concentric annulations in transversal sections. The annual bands are, in some places difficult to follow. This is the case when FOIs approximates the external edges of the tusk, as some authors have recognized (El Adli, 2018). The visibility of some FOIs is discontinuous, which hampers the counting process. We delimitated the analysis of FOIs to the places where they are visible, and we measured their width perpendicular to the increments in neat sagittal sections. By this method, we have inferred annual secretion rate that is transformed into DSR to compare these results with those of the other DSRs obtained from other tusks by means of histological thin sections. This procedure allows us to test the reliability of the FOIs observed on the CAT-102_tusk, by comparing the obtained DSRs with those obtained by different methods from the other tusks. DSRs thus inferred from the CAT-102 tusk does not statistically differ from those calculated on the other tusks (Supplementary table 8,9), thus confirming the reliability of the FOIs observed on this tusk. FOIs measured along the central axis provide a mean value of 7.74±0.64, and measured along the lower edge of 10,.2±2.09. This way, the age of the tusk ranges from 23,81 to 33,59 years (Supplementary table 6).

CAT-77-76-78. This is the longest tusk analysed (Figure 4a, 5, Supplementary table 5). Unfortunately, the preservation of the dentine of this specimen is not good, and the information obtained from it is poor. In this specimen, we measured DSRs and FOIs. DSRs were measured in fragment CAT-76. It was only possible to measure two annual increments in this specimen; these provided a similar rate (mm/y) as in the other tusks analysed. Values of DSRs are the lowest from all tusks (Supplementary table 8, 9), a fact that could be related to the advanced ontogenetic age of this specimen (Supplementary table 6).

Tusk	L inf	L sup	Proximal	0 cm	5 cm	10 cm	15 cm	20 cm	25 cm
CAT-5	47,4	45,8	H	8,90	8,90	8,88			
			W	8,44	7,78	7,82			
CAT-24	97	85	H	17,27	17,25	15,90			
			W	14,05	14,33	13,00			
CAT-51	127	121	H	14,10	14,90	16,00	16,20		
			W	12,80	14,00	15,10	15,37		
CAT-100	245	221	H	34,40	33,30	33,00	30,60	28,80	
			W	28,70	30,60	30,70	29,20	26,10	
CAT-102	260	225	H	37,00	36,09	36,72	37,90	38,36	
			W	32,67	32,20	33,60	34,00	34,64	
CAT-76-78-77	298	260	H	35,20	36,00	37,40	39,00	40,17	39,70
			W	30,10	31,67	32,30	33,00	33,80	32,44

Supplementary table 5. Measurements of the tusks used in this work. L: length; H: height; A: width. H and A are measured every 5 cm, beginning at the most proximal part (pulp cavity). Measurements in mm.

ER (mm/y)	CAT-5	CAT-51	CAT-24		CAT-100		CAT-102		CAT-77-76-78
TYPE DEI	DI	DI	DI	FOI	DI	SOI (FOI)	FOI (2)	FOI (3)	FOI
PLACE ON TUSK	CA	CA	CA	LE	CA	LE	CA	LE	LE
SAMPLES	13,34	10,26	8,1	11,85	12,94	9,64	7,27	13,8	12,23
	13,88	16,93	8,39	10,97	15,55	11,78	7,30	12,2	8,67
	18,71	12,30	7,45	9,53	12,90	9,75	7,55	8,69	
	13,70	7,63	5,96	11	10,30	8,50	7,47	9,73	
	13,53	8,01	7,05	8,35	6,99	14,50	7,07	11,8	
	12,85	9,29	7,61		5,45	9,43	9,70	9,3	
	13,86	7,18	13,13		4,25	8,70	8,49		
	14,03	10,14	13,15		10,98		7,21		
	11,30	15,59			9,66		8,35		
	14,68	8,52			8,49		8,68		
	8,62	9,88			11,9		6,60		
	7,35	7,89			13,3		9,77		
	7,50	8,91					5,95		
		15,20					7,00		
		10,66							
**MEAN ER**	**12,57**	**10,56**	**8,86**	**10,34**	**10,23**	**10,33**	**7,74**	**10,92**	**10,45**
**MEAN ER TUSK**	**12,57**	**10,56**		**9,6**		**10,28**		**9,33**	**10,45**
**YEARS IN TUSK**	**4,00**	**12,03**	**10,95**	**9,3**	**23,95**	**23,72**	**33,59**	**23,81**	**28,52**
**LLE**	**47,40**	**127**		**97**		**245**		**260**	**298**

Supplementary table 6. Extension increments (mm/y) raw data for the tusk sample. Type of extension increment includes: DI= Daily increment; FOI: First Order increment; SOI: Second order increment. In the CAT-100 specimen SOIs have been used to infer FOIs (annual); ER: Extension rate; LLE: Length of tusk from the lower edge. CA= measured on the central axis; LE= measured on the lower edge.

SOI	nº Days/SOI	Given mean	29,53
1	28	Sample mean:	29,29
2	24	95% conf. Interval:	(25,87 - 33,82)
3	33	Difference:	0,01
4	25	95% conf. Interval:	(-3,67 - 3,69)
5	30		
6	31	t:	-0,169
7	34	p (same mean):	0,87

Supplementary table 7. Number of daily increments in FOI of the CAT-100 tusk and results of one-sample t-test, testing whether the mean of nº of daily increments is significantly different from the annual rhythm. Results indicate that the mean number of days in first-order increments does not differ from the annual tempo.

Tusk dentine daily secretion rate (DDSR). The daily dentine secretion rates of *P. falconeri* tusks have been calculated for the six tusks. The range of DDSRs varies from 2 to 7 µm/day (Supplementary table 8). Box-and-whisker plots depicting DSRs (µm/day) of the different *P. falconeri* tusks shows (Supplementary figure 2) that the youngest and smallest tusk (CAT-5) has higher DDSR values while the oldest tusk (CAT-76-77-78) has the lowest values. This is in accordance with the usually higher growth rates in younger, and the lower growth rates in older individuals; this is confirmed by ANOVA analysis (Supplementary tables 8-9).

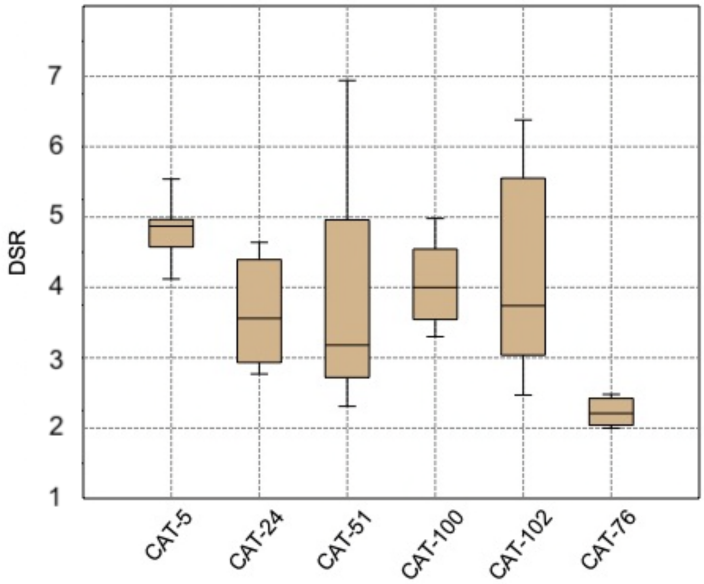

Supplementary figure 2. Box-and-whisker plots depicting dentine DSR (µm/day) of the different *P. falconeri* tusks studied. The only significant differences are the higher DSR of the youngest tusk (CAT-5) and the lower DSR of the oldest one. Horizontal lines denote the median, boxes the interquartile range (25–75 percent quartiles), and whiskers the maximum and minimum values; percentile ranks are computed by linear interpolation between the two nearest ranks.

Tusk	n	Mean	SD	SE	95% CI	Range
**CAT-5**	21	4,69	0,23	0,11	4,33 - 5,04	4,38 - 4,90
**CAT-24**	8	4,03	0,78	0,39	2,78 - 5,29	2,90 - 4,64
**CAT-51**	15	4,84	1,61	0,80	2,28 - 7,40	3,19 - 6,94
**CAT-100**	5	3,80	0,36	0,18	3,23 - 4,37	3,30 - 4,11
**CAT-102**	17	5,04	1,45	0,73	2,73 - 7,35	2,88 - 6,00
**CAT-76**	4	2,22	0,20	0,09	1,90 - 2,54	2,00 - 2,21

Supplementary table 8. Descriptive statistics for DDSRs (μm/d) of the six tusks analysed in this work.

**ANOVA**	**F=5,951**		***p* =0,0001**			
**Tukey's *p***	**CAT-5**	**CAT-24**	**CAT-51**	**CAT-100**	**CAT-102**	**CAT-76**
**CAT-5**		0,057	**0,031**	0,626	0,438	**0,000**
**CAT-24**	4,079		0,999	0,976	0,759	0,171
**CAT-51**	4,407	0,468		0,994	0,827	0,059
**CAT-100**	2,208	1,045	0,757		0,999	0,067
**CAT-102**	2,626	1,901	1,720	0,444		**0,006**
**CAT-76**	6,917	3,395	4,058	3,987	5,191	
**Kruskal-Wallis**	**H= 21,82**		***p* =0,0005**			
**Dunn's *p* (uncorrected)**	**CAT-5**	**CAT-24**	**CAT-51**	**CAT-100**	**CAT-102**	**CAT-76**
**CAT-5**		0,008	**0,006**	0,160	0,081	**0,000**
**CAT-24**	**0,008**		0,688	0,479	0,226	0,063
**CAT-51**	**0,006**	0,688		0,659	0,333	**0,019**
**CAT-100**	0,160	0,479	0,659		0,814	**0,021**
**CAT-102**	0,810	0,226	0,333	0,814		**0,003**
**CAT-76**	**0,000**	0,063	**0,019**	**0,021**	**0,002**	
**Dunn's *p* (Bonferroni)**	**CAT-5**	**CAT-24**	**CAT-51**	**CAT-100**	**CAT-102**	**CAT-76**
**CAT-5**		0,120	0,092	1	1	**0,000**
**CAT-24**	O,120		1	1	1	0,950
**CAT-51**	0,093	1		1	1	0,295
**CAT-100**	1	1	1		1	0,325
**CAT-102**	1	1	1	1		**0,044**
**CAT-76**	**0,000**	0,950	0,295	0,325	**0,045**	

Supplementary table 9. Results of ANOVA and Kruskal-Wallis comparisons for DDSR in the six tusks analysed and pairwise comparisons based respectively on Tukey's and Dunn's post hoc tests (for the latter, both uncorrected and Bonferroni-corrected *p* values are shown). Bolded and coloured values are significant at $p < 0.05$.

Dentine extension rate (DER). Though DSRs show statistically significant, though small, differences between “young” (CAT- 105), “young-adult”, “adult” (CAT-24, CAT-51, CAT100, CAT-120), and (possibly) “senescent” individuals (CAT-76), DERs do not shows differences (Supplementary figure 3, 4, Supplementary table 10). ANOVA analysis of tusk DERs, considering each increment type (daily increment or first order increment), place of measurement (central axis or lower edge) and tusk separately, only shows statistical differences between CAT-5 and CAT-102 (Supplementary table 10). Bonferroni-corrected *p* values clearly show that all other combinations do not show significant differences at $p < 0.05$. CAT-5 DI shows the highest value and CAT-102 FOI CA shows the lowest. However, these two DER records do not differ from the other records sampled on the other tusks. This means that, whatever the periodicity of the increment (daily or FOI) or the place of measurement (on the central axis or on the lower edge), are exceptions of a uniform tendency of lack of ER differences between tusks. Furthermore, considering that the sampled tusks cover an important part of the ontogeny (4 to 34 y) there is no ontogenetic trend in DER. Young and older individuals have similar values. This fact strongly contrasts with the pattern observed in *L. africana* (Figure 4b). This evidence uncovers a pattern of ontogenetic tusk growth rate constancy in *P. falconeri*.

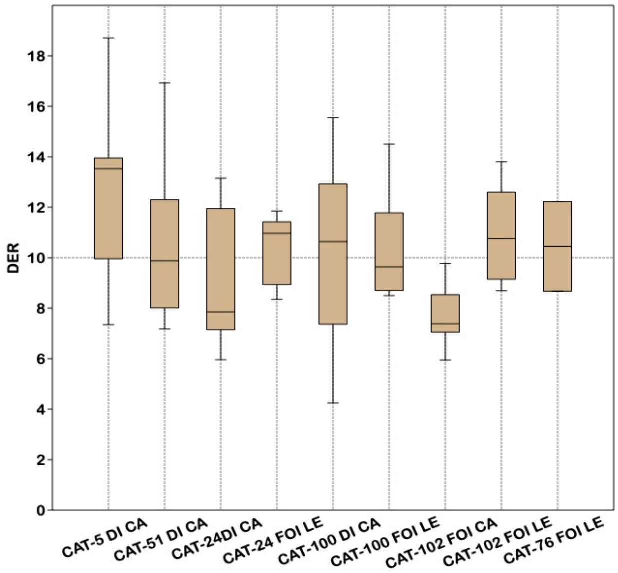

Supplementary figure 3. Box-and-whisker plots depicting DER for the different tusks analysed and for the type of increment used (DI: daily; FOI: first order increment; CA: central axis; LE: Lower edge). ANOVA analysis only shows significant statistical differences between CAT-5 and CAT-102 (Supplementary table 10). Bonferroni-corrected *p* values clearly show that all other combinations do not show significant differences at $p < 0.05$. CAT-5 DI shows the highest value and CAT-102 FOI CA shows the lowest. However, these two DER records do not differ from the other records obtained on the other tusks. Horizontal lines denote the median, boxes the interquartile range (25–75 percent quartiles), and whiskers the maximum and minimum values; percentile ranks are computed by linear interpolation between the two nearest ranks.

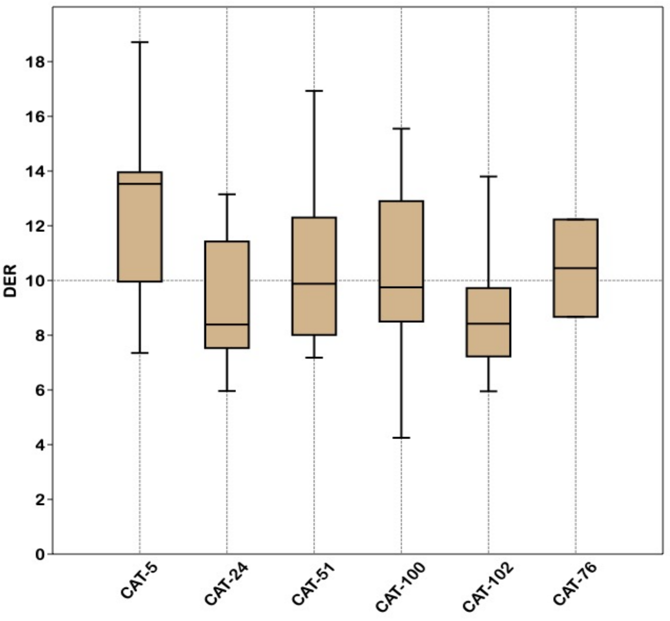

Supplementary figure 4. Box-and-whisker plots depicting DER (mm/y) of the different mean values for the analysed tusks of *P. falconeri* analysed. Horizontal lines denote the median, boxes the interquartile range (25–75 percent quartiles), and whiskers the maximum and minimum values; percentile ranks are computed by linear interpolation between the two nearest ranks.

**ANOVA**	**F=3,463**		***p*=0,007**			
**Tukey's *p***	**CAT-5**	**CAT-24**	**CAT-51**	**CAT-100**	**CAT-102**	**CAT-76**
**CAT-5**		**0,047**	0,381	0,186	**0.001**	0,908
**CAT-24**	4,166		0,879	0,955	0,974	0,996
**CAT-51**	2,756	1,557		0,999	0,347	1,000
**CAT-100**	3,298	1,211	0,445		0,471	1,000
**CAT-102**	5,653	1,066	2,839	2,547		0,952
**CAT-76**	1,450	0,075	0,075	0,130	1,231	
**Kruskal-Wallis**	**H= 14,62**		**P=0,12**			
**Dunn's *p* (uncorrected)**	**CAT-5**	**CAT-24**	**CAT-51**	**CAT-100**	**CAT-102**	**CAT-76**
**CAT-5**		**0,009**	0,108	0,075	**0,000**	0,479
**CAT-24**	**0,009**		0,283	0,295	0,446	0,529
**CAT-51**	0,107	0,283		0,930	**0,047**	0,240
**CAT-100**	0,075	0,295	0,930		**0,043**	0,891
**CAT-102**	**0,000**	0,446	**0,047**	**0,043**		0,312
**CAT-76**	0,479	0,529	0,924	0,891	0,312	
**Dunn's *p* (Bonferroni)**	**CAT-5**	**CAT-24**	**CAT-51**	**CAT-100**	**CAT-102**	**CAT-76**
**CAT-5**		0,144	1	1	**0,004**	1
**CAT-24**	0,144		1	1	1	1
**CAT-51**	1	1		1	0,706	1
**CAT-100**	1	1	1		0,647	1
**CAT-102**	**0,004**	1	0,706	0,647		1
**CAT-76**	**1**	1	1	0,900	1	

Supplementary table 10. Results of ANOVA and Kruskal-Wallis comparisons for DER in the six tusks analysed and pairwise comparisons based respectively on Tukey's and Dunn's post hoc tests (for the latter, both uncorrected and Bonferroni-corrected *p* values are shown). Bolded values are significant at $p < 0.05$.

***P. falconeri* tusk growth rates compared with those of continental elephants.** Probably, one of the most unexpected results of this work is the absence of relevant differences in tusk GR during the ontogeny of *P. falconeri*, an unusual fact in elephants. In spite of some anecdotic records (Colyer and Miles, 1957), direct evidence of DERs of extant elephant tusks is almost inexistent. The only available evidence is the information that correlates tusk length with age. However, this is not a real extension rate, as this approach does not take into account the material lost during the wear process to which the tusks are exposed during life; anyway, it is an approximation. GR inferred from tusk length and age data in these estimates is likely lower than DERs measured by other methods (dentine increments or 14C, for example). A study (Whyte and Hall-Martin, 2018) on the growth pattern of *L. africana* tusks from the Kruger National Park allows distinguishing between growth patterns of males and females (Figure 4b). It can be observed that there is a clear sexual dimorphism in the growth rate. The rate is considerably higher in the first ten-to-twelve years of life, and it decreases from that age onwards until senescence. The only direct measurement of extension rate of *L. africana* tusks is that inferred by means of 14C (Uno et al., 2013). However, the GRs of the two tusks analyzed by this method (Uno et al., 2013) fit the model based on length and age data (Whyte and Hall-Martin, 2018) (Figure 4b). When GR data of fossil continental elephant taxa are included, they also fit the *L. africana* pattern, though with minor differences (Figure 4b). The data on *P. falconeri* are mean values of individual tusks and the ontogenetic ages were estimated using the respective DERs and the preserved lengths. In all cases these are minimal ages considering that all specimens are incomplete for different reasons (they are either fragments, or they are partial tusks broken and / or worn during lifetime, such as CAT-120 for instance).

It is remarkable that the GR of the Spinagallo tusks not only does not change over ontogeny, but it is even lower than that of extant female *Loxodonta* already in the senescence phase (Figure 4b). This surprising result is congruent with the growth rate patterns of *P. falconeri* tibiae. In the tibiae, differences in GR between immature and mature individuals are minor (the growth rates provided by the segmented equation exponents from young/adult individuals are 5/2 in *P. falconeri*, while in L. *africana* they are 49/2 (Figure 3a). Furthermore, this growth pattern with little or no differences between young and mature ages parallels those of other insular taxa as far as hitherto studied. This is the case of the arctic reindeer from Svalbard archipelago (*Rangifer tarandus platyrhynchus*) that do not show the relevant ontogenetic changes in histological features during the last growth cycle before reproductive maturity seen in other artiodactyles, which reflects the slowdown of growth as maturity approaches (Jordana et al., 2016). Similarly, the extinct skink from Cabo Verde did not show an increased juvenile growth rate but rather constant growth over ontogeny (Andreone and Guarino, 2013).

P. falconeri lifespan estimation. To infer the longevity of *P. falconeri* on the basis of the longest Spinagallo tusk, we relied on the DERs of the six tusks analysed. The absence of significant statistical differences between the tusk DERs (Supplementary table 11) during ontogeny makes it easy to infer the lifespan using the length of the longest tusk. The direct application of DERs to the longest tusk provides an approximation to the longevity (Supplementary table 12). The mean value of tusk DERs of *P. falconeri* is 10.16±0.65 mm/y. Applying this GR to the longest tusk of *P. falconeri* (Ambrosetti, 1968; L= 710 mm), and considering that the tusk suffered wear during life, yields a minimal lifespan of 67.96 years (62.55 – 73.36 95% CI) (Supplementary table 12,13). This result clearly suggests that, contrary to widely accepted previous hypotheses (Raia, 2003; Larramendi and Palombo, 2015; Bromage et al., 2002; Palombo, 2007) *P. falconeri* had a long lifespan similar to or even longer than extant elephants. Lifespan calculations of fossil elephantid species by means of highly effective lifespan biomarkers from some modifications of DNA (including genes critical for longevity) (Mayne et al., 2019), estimate the longevity of the woolly mammoth (*M. primigenius*) and the straight-tusked elephant (*P. antiquus*), putative ancestor of *P. falconeri*, as 60.0 years (Mayne et al., 2019), which is within range of the modern-day elephant taxa of similar size (Figure 4 c). This suggests that *P. falconeri* had a similar or an even longer lifespan compared with its putative ancestor. In absolute values, the lifespan of *P. falconeri* is similar to that of extant elephants and differs importantly from predictions based on allometry and body mass (Supplementary figure 5; figure 4 c, d; table 3).

	n	Mean	SD	SE	95% CI	Range
DER	80	10,16	2,94	0,33	9,51 - 10,82	4,25 - 18,71

Supplementary table 11. Descriptive statistics of the pooled DER of the six tusks analysed. The absence of significant differences in the DER of the tusks analysed allow to infer the basic statistics of pooled data.

Tusks / (mm/y)	DailyER	FOI	LS TUSK DER	LS TUSK FOI
CAT-5	12,57		56,48	
CAT-51	10,56		67,23	
CAT24	8,86	10,33	80,14	68,73
CAT-100	10,23	10,33	69,40	68,73
CAT-102		10,92		65,02
CAT-77-76-78		10,45		67,94

Supplementary table 12. Mean values for dentine extension type (Daily and FO) and inference of the age for the longest Spinagallo tusk (710 mm). LS TUSK DER: Longevity estimation based on Daily Extension Rates; LS TUSK FOI: Longevity estimation based on First Order Increments.

	n	Mean	SD	SE	95% CI	Range
Lifespan	11	67,96	6,46	2,28	62,55 - 73,36	55,48 - 80,14

Supplementary table 13. Descriptive statistics for lifespan inference from pooled data of the six tusks analysed in this work.

Scaling of lifespan against body mass in P. falconeri, extant elephants and other ungulate mammals. Log–log phylogenetic generalized least square regressions (PGLS) of lifespan against body mass is the best method to account for longevity in the context of body mass (Müller et al., 2011; Western, 1979) (See Supplementary material 8). Thus, a comparative allometric analysis of longevity and body mass across ungulate mammal species (ungulates and proboscideans) using AnAge database (Tacutu et al., 2018), provides the best framework to interpret the scaling relationships between lifespan and body mass of *P. falconeri*. From the AnAge data base (Tacutu et al., 2018), we updated the body masses of the extant elephant species used for comparison, *L. africana* and *E. maximus*. A recent revision of body masses of fossil and extant elephants (Larramendi, 2016) suggests that the mean body masses for extant elephants (mean values for male plus females) used in AnAge data base (Tacutu et al., 2018) are underestimated. For this reason, and considering that the use of correct BM of the comparative sample is critical, we used the body masses proposed in recent revisions (Larramendi, 2016). For extant elephants, we used AnAge data base (Tacutu et al., 2018).

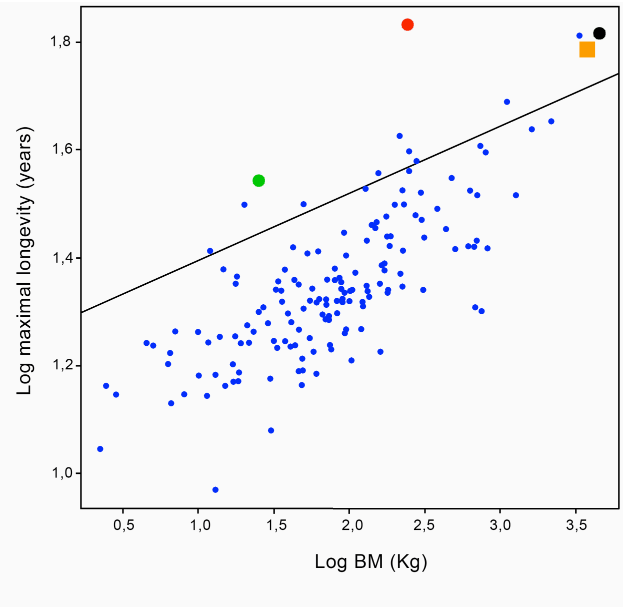

Supplementary figure 5. Phylogenetic generalized least square regressions (PGLS) of longevity (y; years) against adult body mass (x; kg) in log10. (Results of PGLS: see fig 13) in ungulate mammals (small blue dots), orange square *L. africana* (AnAge data base, Tacutu et al, 2018), black dot *H. maximus* (AnAge data base, Tacutu et al, 2018), red dot *P. falconeri*; green dot *Myotragus balearicus* from the Pleistocene of the island of Mallorca (Marin-Moratalla et al., 2011).

6. Age at sexual maturity in *P. falconeri*.

Age of epiphyseal fusion of tibiae in P. falconeri and extant elephants. Developmental maturity. The sample of 29 tibiae of *P. falconeri*, aged by skeletochronology, allows inferring the timing of the epiphyses/diaphysis fusion until the age of 23 years. There is considerable variation in the age of onset of epihysieal fusion: the earliest evidence of the beginning of the process of epiphyseal fusion in *P. falconeri* is the partial fusion of the proximal epiphysis at 14 years (T179), while T? does not show any fusion of either proximal nor distal epiphyses at 22 years (Supplementary table 1). The first evidence of distal fusion is at 22 years in T203, which lacks the proximal epiphysis, while the slightly younger T181 only preserves the proximal fused epiphysis. This pattern is not very different from that observed in *L. africana*, where the first evidence of epiphyseal fusion has been recorded at 15 years in females, but with a number of male individuals still unfused proximally and distally in their early-mid twenties[26]. In *E. maximus*, the onset of the fusion of the proximal epiphysis is at 25 years, apparently later than in *P. falconeri* and *L. africana*, though this may simply reflect sex differences in the timing of fusion owing to a male-biased dataset (Supplementary figure 6). This suggests that *P. falconeri* does not differ in timing of epiphyseal fusion of tibiae from extant *Loxodonta*, or possibly *E. maximus*, despite the important size differences.

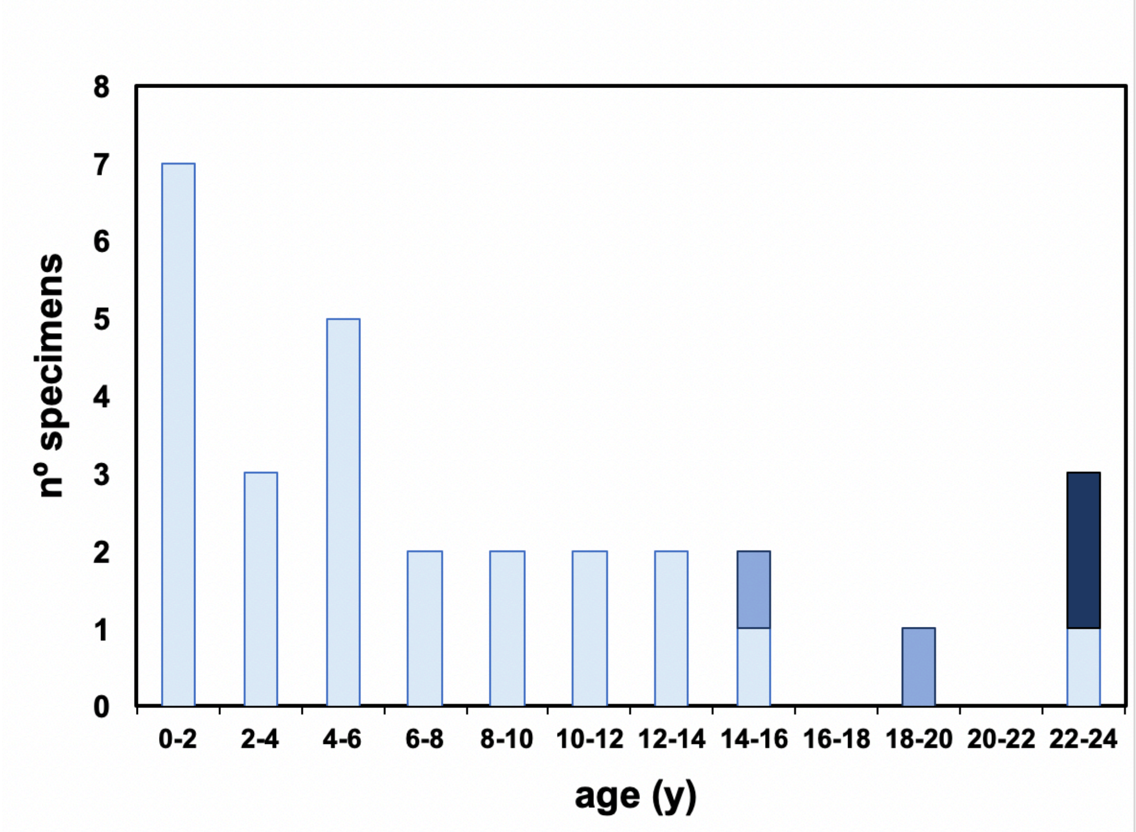

Supplementary figure 6. Histogram showing the age distribution of fusion status of the epiphyses in the sample of 29 aged tibiae from Spinagallo. Light blue: epiphysis unfused; medium blue: fusing; dark blue: fused. (In this sample, proximal and distal epiphysis show the same pattern of fusion).

Piecewise regression and the inference of age at sexual maturity in P. falconeri. Following the same rationale as for growth models, the meaning of Breakpoints (BP) in the analysis of reproductive ontogeny marks the transition from high to low growth rates, which in mammals is usually close to the age of sexual maturity. Considering that organisms are limited by energy, both in absolute terms and in the rate at which energy can be acquired and processed (Case, 1978; Lemaître et al., 2015), somatic and reproductive growth should be separated in time to maximize reproductive output (Kozłowski and Wiegert, 1987). Therefore, during sexual maturation an important part of the energy devoted to growth is channelled to reproduction and associated traits (such as energy storage) while somatic growth rate slows down. Accordingly, models of biphasic somatic growth have been proposed where maturation is accompanied by a deceleration of growth due to the allocation of energy to reproduction (Quince et al., 2008; Quince et al., 2008). Theory (Stearns, 1999) and empirical evidence from reptiles (Lee and Werning, 2008; Erickson et al., 2001; Griebeler and Werner, 2018; Padian et al., 2013) and mammals (Lee and Werning, 2008; Erickson et al., 2001; Griebeler and Werner, 2018), support the concept that the inflection point in the growth curve indicates the beginning of reproduction. In fact, the breakpoints obtained from piecewise regression can be interpreted as a threshold value beyond which relevant energetic changes occur in the organism.

Analysis of diaphyseal length (TDL) in L. africana tibiae. Sexual maturation in extant Loxodonta and biological meaning of the inflexion point. Our results of piecewise regression of TDL in *L. africana* provide statistically significant differences between two linear regression equations with a single breakpoint (BP). BP is placed at 8.13 years, and separates two regression equations (Figure 3a) with different slopes. The pre-BP equation has a high exponent (41.62) compared with the low exponent of the post-BP (2,25), indicating a considerable reduction of the growth rate post-BP (Table 2).The placement of the BP in the ontogenetic process of sexual maturation of the sample of *L. africana* from Kruger National Park (Smuts, 1975; Freeman et al., 2008) suggests that TDL BP is placed early in ontogeny in respect to the age of sexual maturity and first calving, coinciding approximately with the beginning of the pubertal phase of sexual development. The initial onset of sexual maturity is a period in the life of the individual which is difficult to define. For example, from the point of view of reproductive physiology, the initial production of viable gametes would be sufficient to mark the onset of sexual maturity. Actually, the only in-depth analysis of reproductive ontogeny in *Loxodonta* comes from the physiological status of reproduction of a culled sample of *L. africana* from Krüger National Park (South Africa) (1975-1995) (Smuts, 1975; Freeman et al., 2008).

Two phases of the ontogeny of sexual maturation have been identified (Smuts, 1975) (Figure 3b). Thus, during the pubertal phase, the process of physical changes through which the body matures into an adult body capable of sexual reproduction, females show at least one follicle > 5 mm in the uterus, but no *corpora lutea* or *corpora albicantia*. The following phase of sexual maturity, when females have the capacity to

ovulate, can be determined by the presence in the ovaries of *corpora lutea* and/or *corpora albicantia* (Smuts, 1975; Freeman et al., 2008; Whyte, 1982). The pubertal phase of reproduction is between 7 and 12 years of age, and sexual maturity between 7 and 15 years of age (Laws and Parker, 1968). Using the percentage of pregnant and reproductively active cows, the pubertal interval has been suggested to be around 12 years of age, when 50% of the population is reproductively active and, on average, cows attain maturity (Smuts, 1975) (Figure 3b). This indicates that sexual maturity (ovulation) in this population has a very low incidence at 6-7 yr, but increases from 8yr until 12 yr (Figure 3b). Data on age of sexual maturity and/or first calving in extant *Loxodonta* are known from other populations in Africa. The age of sexual maturity in elephants varies in different populations across Africa (Eltringham, 1982) and there is considerable individual variation in the age at first birth, but most females conceive their first calf between 11 and 13 years of age (median of 12 yr) (Moss, 2001). Nevertheless, some females can conceive below 10 yrs though the probability is of only around 1%. Female elephants can potentially conceive as young as 7 years old in Amboseli; however, such conceptions were rare, and calves born by these young females had only 50% survival probability (Moss, 2001). These patterns are congruent with a mean age at sexual maturity of 12 years proposed by other authors (Smuts, 1975). Hence, the BP of TDL at 8.13 years in *L. africana* is placed early in ontogeny within the pubertal reproductive phase, before sexual maturity and first calving. The placement of the inflection point on the asymptotic region on growth models early in ontogeny with respect to sexual maturity and first calving seems in fact to be a generalized phenomenon in amniotes as some authors observed (Erickson et al., 2001). Our results confirm these observations and suggest that the BP in the piecewise regression of *L. africana* represents the beginning of the draining of energy from somatic growth to reproduction that coincides with puberty or the puberty/sexual maturity transition. Thus, the beginning of the energy drainage toward reproduction in *Loxodonta* starts in the pubertal phase of sexual development.

Piecewise regression of P. falconeri tibiae and comparison with L. africana. To explore possible breakpoints (BPs) at a specific age during sexual development of *P. falconeri*, and following the procedure used in *L. africana*, we used TDL ontogeny and age estimation from the sample of 29 aged tibiae from Spinagallo. The piecewise regression for TDL provides a later BP position in ontogeny for *P. falconeri* (11.03 yr) (Figure 3a) than for *L. africana*. Considering that the piecewise regression based on TDL growth during ontogeny of *L. africana* and *P. falconeri* follows the same methodology and is based on the growth of the same anatomical element, the results are directly comparable, and by consequence the BP reflects the same biological event. The results strongly suggest that the beginning of the puberty/sexual maturity transition in *P. falconeri* is postponed to the age of 11 years, three years later than *L. africana*, indicating a delayed maturation in respect to *L. africana*. Because the mean value of sexual maturity in the *Loxodonta* sample of the Kruger National Park (Smuts, 1975) is 12 years, 4 years later than the position of the BP, the mean age at sexual maturity in *P. falconeri*, by analogy, may have been close to 15 years.

Finally, there is a remarkable difference in slopes in the piecewise regression equation of the prenatal phase between *L. africana* and *P. falconeri*. In *L. africana*, the exponent of the initial growth phase is much higher (41.62) than in *P. falconeri* (5.77), which is comparatively very low and demonstrates the extremely low growth rate of this insular taxon. Interestingly, the growth posterior to the break point is similar in both taxa, being even lower in *L. africana* than in *P. falconeri*. The small differences in GR between pre- and post-sexual puberty in P. falconeri is a phenomenon similar to that observed in tusks growth rate differences between young and adult individuals, which again contrasts with the important change in GR observed in *L. africana* (Figure 3a).

P. falconeri age at sexual maturity in an allometric context. Taking into account the important size difference between continental and island elephants, life-history traits should be analysed in the context of body size rather than by its absolute value (Müller et al., 2011; Western, 1979). Log–log phylogenetic generalized least square regressions (PGLS) is the best method to account for age at sexual maturity in the body mass context, considering phylogenetic information. The PGLS (see Supplementary material 8) of log10 age at sexual maturity (y) against log 10 body mass (x) for extant ungulate including elephants (Figure 3c) shows that compared with other ungulate, elephants attain sexual maturity relatively late even taking into account their elevated body mass. Insular herbivores such as *P. falconeri* and the insular extinct bovid from the island of Mallorca (Balearic Islands, Spain), *Myotragus balearicus*, however, are complete outliers as they reach sexual maturity much later than predicted from body mass (Figure 2c) (Marín-Moratalla et al., 2011).

7. Molar growth rates and crown formation time of *P. falconeri* cheek teeth

Plate formation time (PFT) & crown formation time (CFT) in elephantids. Enamel extension rate of P. falconeri cheek teeth. The special characteristics of elephantid teeth make calculation of crown formation times (CFT) difficult. During development, elephant teeth not only increase in plate height but they also grow by horizontal, sequential accretion of new plates at the same time as the tooth wears out through grinding. Thus, CFT depends on both, the plate formation time (PFT) that is the rate at which a single plate increases in height, and the rate of plate addition, the rate at which plates are added to build the whole tooth. In dP/2 and dp/3 teeth are almost fully formed at birth, whereas in dp/4 to M/3, the staggered nature of crown formation can be observed postnatally (Figures 6, 7). CFT calculation is further complicated because the number of anterior plates that enter into wear while the posterior ones are still under formation depends on the tooth. Hitherto, CFT has only been calculated for *L. africana* (Uno et al., 2014). Using isotopes (14C), PFT for four lamellae of a lower M/3 (TE-95) and for two plates in a second M/3 (R37) has been calculated as 7.3 ± 0.6 years (mean value of the unworn plates) and CFT of the entire M/3 has been inferred as 10 years (Uno et al., 2014) (based on 14C ageing of the mesial root of the TE-95 specimen). This shows that mean PFT and CFT are not equivalent and that it is the delay in the development of

successive plates that accounts for this 27% difference between PFT and CFT. Because of these difficulties in reconstructing CFT, the few histological studies hitherto conducted on elephant teeth are limited to estimation of PFT (Uno et al., 2014; Jordana et al., 2015). With this in mind, we used PFT in our comparative analysis while our estimation of M/3 CFT in *P. falconeri* is only an approximation, based on Uno's model (Dirks et al., 2011) of PFT and CFT in *L. africana*. However, some aspects must be taken into account to correctly infer PFT. First of all, the plates must be fully grown (they must have at least incipient roots) and second, they should be unworn because an incomplete apex leads to underestimation of PFT. A second requirement is the correct anatomical identification of the tooth. From dp/3 to M/3 there is an increase in crown height which has a bearing on PFT. Thus, milk teeth and permanent first molars have lower crowns than subsequent molars which are expected to have higher PFTs. Therefore, in comparative studies it is mandatory to compare PFT of homologous teeth if time and not rate of formation is calculated. However, too frequently these factors are not sufficiently taken into account (Jordana et al., 2015; Dirks et al., 2011). To avoid these problems, we densely sampled several fully-grown plates without wear (Figure 6), reliably assigned to two identified teeth, throughout their full lengths. We chose a lower third molar (M/3) because the only available information that allows comparison of our insular dwarf elephant with a large continental taxon comes from the 14C aged lower M/3 of *L. africana* mentioned above (Uno et al., 2014). We further chose an upper fourth premolar (dP4/; **Supplementary** figure 7) from the milk dentition to account for any possible change in one or more of the growth parameters (DSR, EER, PFT) and thus to gain a global perspective of the time of formation of *P. falconeri* teeth.

Our results confirm previous findings that there are no differences in DSR between small and large elephants (Jordana et al., 2015; Dirks et al., 2011). EER varies from lamella to lamella, with a tendency to decrease toward the posterior part of the crown in both the lower M3 and the upper dp4 (Figure 6, 7). Within the same plate, EER shows first a rapid increase from apex to the middle of the plate followed by a constant but accentuated decrease towards the roots (cervix). This differs somewhat from the pattern described for *P. cypriotes* (Dirks et al., 2011), where extension rate is found to speed up towards the end of plate formation. ANOVA analysis ($F(5,60) = 6{,}938$, $p = 0.0001$) and Tukey post hoc test reveals that M/3 9A,M/3 9P and dp4/P have a significantly lower EER than M/3 8A-8P and dp4/A. This suggest that posterior plates have lower EER than anterior plates. However, comparing the total EER sample of the lowerM3 with that of the upper dp4/, a t-test indicates that there are no significant differences between both teeth ($t\ (64) = -1{,}336$, $p=,186$). The result that milk and definitive molars have similar EERs is another evidence of the only minor differences in GR ontogeny in *P. falconeri* (Figure 6e, 7). In Figure 6c growth in plate height is plotted against plate formation time (years) in the upper dp4/ (CAT-183) and in the lower M/3 (CAT-114) (Red dots). Plates of both teeth show a similar growth trajectory with a low extension rate, suggesting that the pace of plate growth in these two teeth is similar. The only variation between both teeth is the formation time of the plates. This indicates that differences in crown height between teeth is a function of the time they take to form and not of the rate at which they

grow (Figure 6c). For this reason, taxon-specific conclusions for CFT should be reached with caution; they should be based on complete plates and they must take into account the tooth type. As we show bellow, plates of the upper dp4/ and the lower M/3 of *P. falconeri* provide long PFTs, supporting the hypothesis of prolonged enamel deposition related to an extended longevity.

Comparison of PFTs between P. falconeri and L. africana. The only available information on PFT and CFT of *L. africana* or other extant elephants, comes from a lower M/3 aged by means of 14C isotopes (Uno et al., 2014). This is why we calculated EER and the PFT in a lower M/3 of *P. falconeri*, using two unworn and completely formed posterior plates (P8 and P9) comparable to those used for *Loxodonta*, to directly compare PFTs between the fossil insular dwarf and the extant large continental elephant. In Supplementary table 14 we show the main variables calculated in the CAT-114 specimen of *P. falconeri* and the relevant accessory information to correctly interpret EER (or GR in the case of *L. africana*), and PFT. Particularly relevant are plate wear and developmental degree, which determine if the PFT record should be considered complete or only partial. In the latter case, PFT is only a minimal estimate. This is the situation with the plate 9P of the *L.* africana TE-95 specimen (Uno et al., 2014). Considering that in the specimen of *L. africana* plates 2 and 4 show minimal wear and plate 9 is not completely formed, PFT in this taxon would be somewhat higher than that reflected by the PFT data (Uno et al., 2014). Considering that the wear is minimal, however, this fact would have an only low impact on the mean PFT for this tooth. Comparison of EER and PFT of M/3 between *L. africana* and *P. falconeri* shows that EER is lower in *P. falconeri*, which resulted in an extended PFT in this taxon despite the notably smaller size of the plate compared with that of *L. africana*. (Figure 6d, supplementary tables 15, 16). From the M/3 data of *L. africana* a formation time not lower than ten years has been inferred (Uno et al., 2014). The higher PFT mean of *P. falconeri* suggest a longer formation time for the M/3 of this insular endemic taxon compared with continental elephants. The upper dp4/(CAT-183) PFT also provides interesting information on the developmental timing of the *P. falconeri* dentition (Figure 7). Unfortunately, there are no direct data for PFT of the milk dentition of extant elephants. However, some data on dental development can be used to compare with *P. falconeri*. The posterior two plates of the *P. falconeri* specimen CAT-183 are not fully formed and can therefore not be used (Figure 7). The anterior plate is completely formed, but its poor preservation makes any calculation of PFT impossible. The second plate provides a formation time of 4 years. This information is not known with precision for extant elephants; nevertheless, considering that dp4/ in *L. africana* starts calcification perinatally (Eales, 1930, Sikes, 1966), and that at Laws's elephant age VI (4 years) (Laws, 1966, Stansfield, 2015) the wear affects all plates and consequently, all plates are formed at 4 years, this strongly suggests a dp4/ plate formation time similar to that of *P. falconeri*. The similarities in M/3 and dp4/ PFTs between *P. falconeri* and *L. africana* are consistent with a long lifespan inferred from dentine extension rate in tusks.

	PLATE	PLATE LENGTH (mm)	GR (mm/y) ± 2σ	PFT (y)	PLATE STATUS	LENGTH MEASURRED	CFT (y)
***L. africana* (1)**			14c				
**(TE-95)**	2A	101,00	14,90 ± 0,54	6,80	minimal wear	complete	
	4P	116,00	14,60 ± 0,59	7,90	minimal wear	complete	
	7P	123,00	16,30 ± 0,14	7,50	no wear	complete	
	9P	111,00	16,20 ± 0,14	6,90	root still open	incomplete	
**Mean**				7,27			**10**
P. falconeri			EER mm/y				
**(CAT-114)**	8A	71,23	10,68 ± 1.36	8,81	no wear	complete	
	8P	76,37	10,03 ± 1.96	9,99	no wear	complete	
	9A	67,20	7,91 ± 1.52	9,10	no wear	complete	
	9P	66,32	6,72 ± 0.74	7,91	no wear	complete	
**Mean**				8,95			**11,47**

Supplementary table 14. Measurements and variables used for comparison of M/3 growth rate and PFT between *L. africana* and *P. falconeri*. Values of M/3 plate formation time in *P. falconeri* and *L. africana* (Uno et al., 2013). In the second column, the numbers indicate the plate position (A: anterior lamellae; P: posterior lamellae). PFT expressed in years.

CFT in large continental and dwarf insular elephants. As we have shown above, the total sample of upper milk and lower definitive molar plates of *P. falconeri* have similar mean EERs, which enables data from different teeth to be plotted together in a size – time graph. Growth in plate height plotted against plate formation time (years) for upper and lower teeth pooled together and using all available data from complete and incomplete plates of insular and continental taxa (including *Mammuthus columbi* from Dirks et al., 2011 and *P. antiquus,* the putative sister taxon of *P. falconeri* and *P. cypriotes* from our own data) show a good fit to linear regression (y = 17.226x – 0.0022, R. = 0.97) (Figure 6c). The insular elephant sample includes a fragmented molar plate of *P. cypriotis* (Cyprus) (Dirks et al., 2011) and all analysed specimens of *P. falconeri* (this work), and fits a polynomic regression of second-order (y = -0,2934x2 + 9,8626x + 2,6693, R. = 0.98). While the growth rate in plate height is different in continental and in insular elephants, the growth rates are homogeneous within both groups. Thus, continental and insular taxa follow two clearly different growth trajectories with different rates: a high rate in large continental elephants and a low rate in dwarf insular taxa (Figure 6c). This phenomenon was described previously (Dirks et al., 2011), comparing the growth rates in plates of *M. columbi* (continental) and *P. cypriotis* (insular). The relatively slow enamel extension rates of *P. falconeri* and *P. cypriotis*, hence, indicate that the teeth of dwarf elephants grow relatively slowly compared to the teeth of their large ancestor and other continental elephants. Nevertheless, PFT does not differ substantially between continental and insular taxa, which is not in line with the shorter PFT described for *P. cypriotis* (Dirks et al., 2011). However, the selected plate of *P. cypriotis* was broken and it is unknown how much is lost (Dirks et al., 2011). This is supported by the comparatively high EER values close to the end of plate formation found in *P. cypriotis* (Dirks et al., 2011) that might suggest formation was incomplete (see above). Furthermore, the attribution of this tooth to either a M2/ or a M3/ was tentative and, as we have shown above, plate length depends on both the tooth itself and its position within the tooth, and has a considerable bearing on the time of formation. Therefore, we consider not the time

but the rate of plate formation in the Cypriote dwarf elephant to be the most reliable parameter for comparison. Insular taxa combine a slower pace of dental growth (plate height against plate formation time) with an unchanged time of formation. The slower extension rate of insular elephants (Figure 6c) reflects a slow rate of tooth formation, combined with a prolonged life history relative to body size.

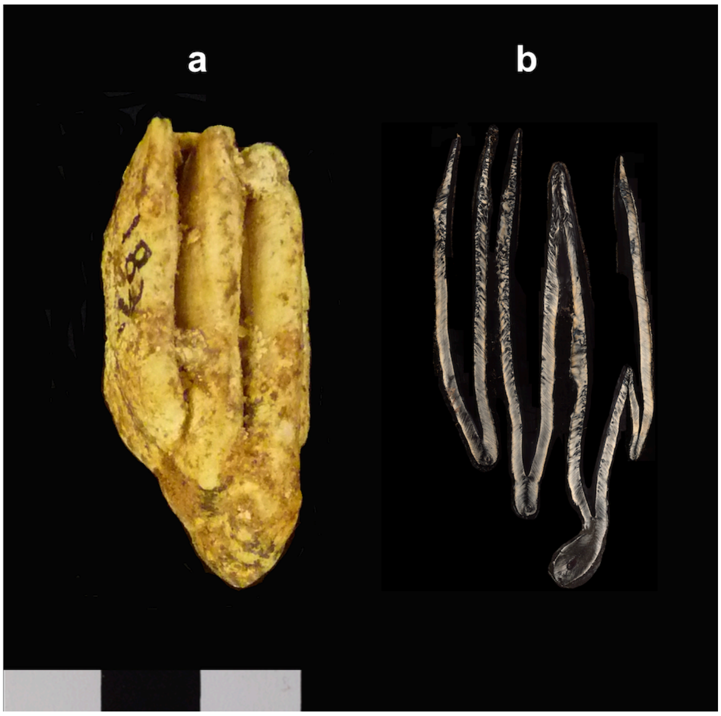

Supplementary figure 7. a: Lingual view of the anterior three plates of an upper dp4/ (CAT-183). b: Sagittal thin section of CAT-183. b: Zeiss Scope A1 microscope with integrated digital camera (AxioCam ICc5). Graphic scale 3 cm.

	n	Mean	SD	SE	95%CI	Range
**M3**						
**P8A**	15	10,68	2,63	0,68	7,08-12,14	11,17-14,55
**P8P**	6	10,03	2,39	0,98	7,52-12,55	7,76-14,40
**P9A**	13	7,91	2,27	0,76	6,24-9,57	4,20-11,60
**P9P**	14	6,72	1,37	0,37	5,93-7,50	4,70-9,30
**dp4**						
**AP**	9	11,3	2,75	0,92	9,17-13,40	7,90-15,33
**PP**	9	8,17	1,87	0,62	6,73-9,61	5,20-10,80

Supplementary table 15. Descriptive statistics for EER of the plates of lower M/3 (CAT-114) and upper dP4/ (CAT-183) of *P. falconeri* from Spinagallo cave.

	n	Mean	SD	SE	95%CI
P. falconeri	4	8,95	0,86	0,43	7,59-10,32
L. africana	4	7,27	0,52	0,26	6,45-8,10

Supplementary table 16. Descriptive statistics for lower M/3 plate formation time (PFT, years) in *P. falconeri* and *L. africana* (Uno et al., 2013). Results from an independent-samples t-test conducted to compare the mean M3 PFT for *P. falconeri* and *L. africana*. There was a significant difference in the means of M/3 PFT of *P. falconeri* (8,86 y) and *L. africana* (7,27), t(6)= 3,34, p=0,015.

8. Phylogenetic generalized least square regressions (PGLS).

To test the impact of an independent variables (body mass) on a dependent variable (age at sexual maturity, mean body mass growth rate from birth to maturity, MBMGRB-M and longevity) while controlling for potential influence of phylogeny in any of these variables (non-independence of the residuals), phylogenetic generalized least square regressions (PGLS) were performed to evaluate potential phylogenetic signal. The phylogenetic generalized least square regression (PGLS) and Pagel's lambda (Pagel, 1999) and Blomberg's K (Blomberg et al., 2003) was performed using package phytools v.0.7-90 using 10000 simulation rounds to compute the p value.

Results from PGLS (Supplementary figures 9-14) show that in the case of continental ungulates the distribution of residuals shows an important degree of variance and residual dispersion. In the phylogenetic-corrected residuals the insular taxa, goal of this study (*P. falconeri*; *M. balearicus* for comparison) are still identified as clear outliers for the regressions of ASM, MBMGRB-M and longevity against body mass, as consequence of the high value of the residuals of these parameters in respect to body mass even after phylogenetic correction (Supplementary figures 8-13, Supplementary table 17).

These results suggest that the insular taxa show an evolutionary response that is independent from phylogenetic constraints and, instead, a consequence of their life history adaptation to the insular ecosystem. The result is further strengthened by the recovered phylogenetic signal showing that, despite the presence of a phylogenetic patterning in the distribution of the variables (Pagel's lambda close to 1 in all instances) (Table 17), the evolution of these traits is most likely more driven by function rather being stochastic. This is due to the low values obtained for the Blomberg's *K* ($K < 1$ in all instances) (Table 17), which denotes an accumulation of the variance within clades, thus implying that neighbor taxa resemble one another less than expected and that the mode of evolution is not aleatory, possibly as the results of homoplastic adaptations.

Scaling of the mean body mass growth rate from birth to maturity (MBMGRB-M):

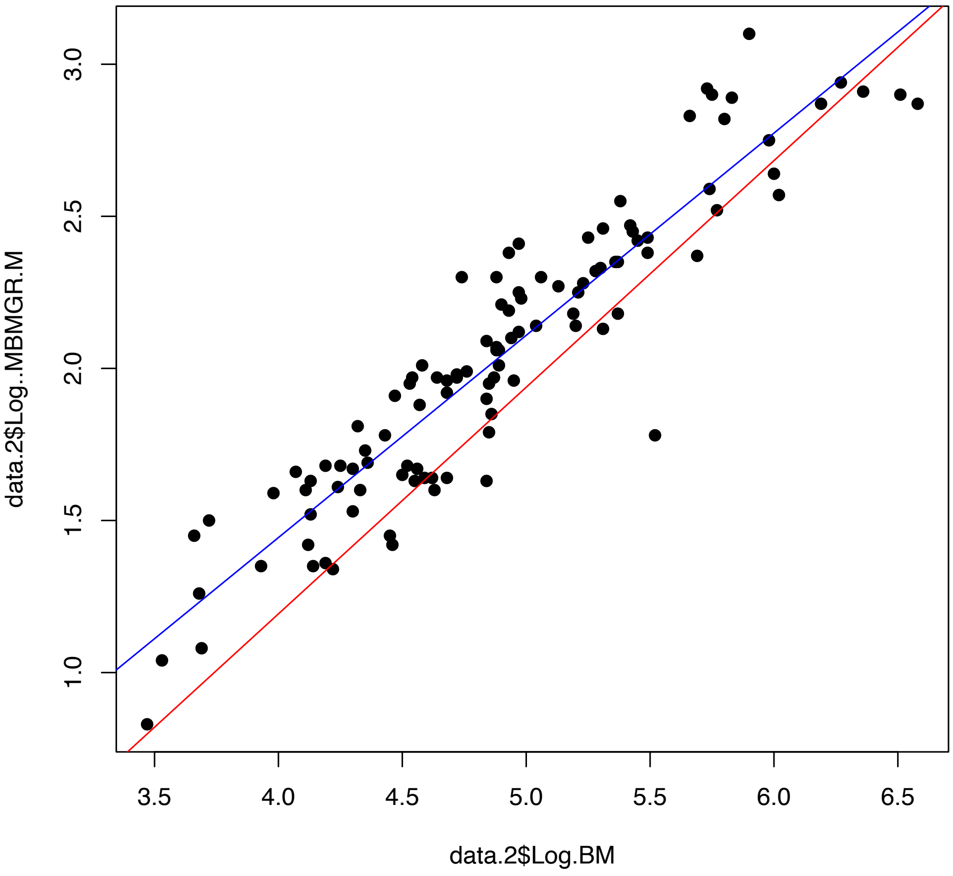

Supplementary figure 8. Scatterplot of MBMGRB-M (adult body mass-neonatal body mass, gr./age from birth to sexual maturity, days) (y) against adult body mass (x) in log10. Results of PGLS: Y=a+bx, Intercept (a) -1.787490, Slope (b) 0.745115, Slope (b) 0.745115, Multiple R-squared: 0.8626, Multiple R-squared: 0.8588, Adjusted R-squared: 0.8574, F-statistic: 665.5 on 1 and 106 DF, p-value: < 2.2e-16 F-statistic: 644.4 on 1 and 106 DF, p-value: < 2.2e-16. The scaling of the mean body mass growth rate from birth to maturity (MBMGR, expressed in grams/day) in respect to adult body mass in a large sample (n=107) of species of ungulate mammals (Data from PanTheria, Jones et al., 2009), provides a way to directly compare MBMGRB-M across ungulate taxa. Blue line: Regression line without PGLS; Red line: Regression line with PGLS.

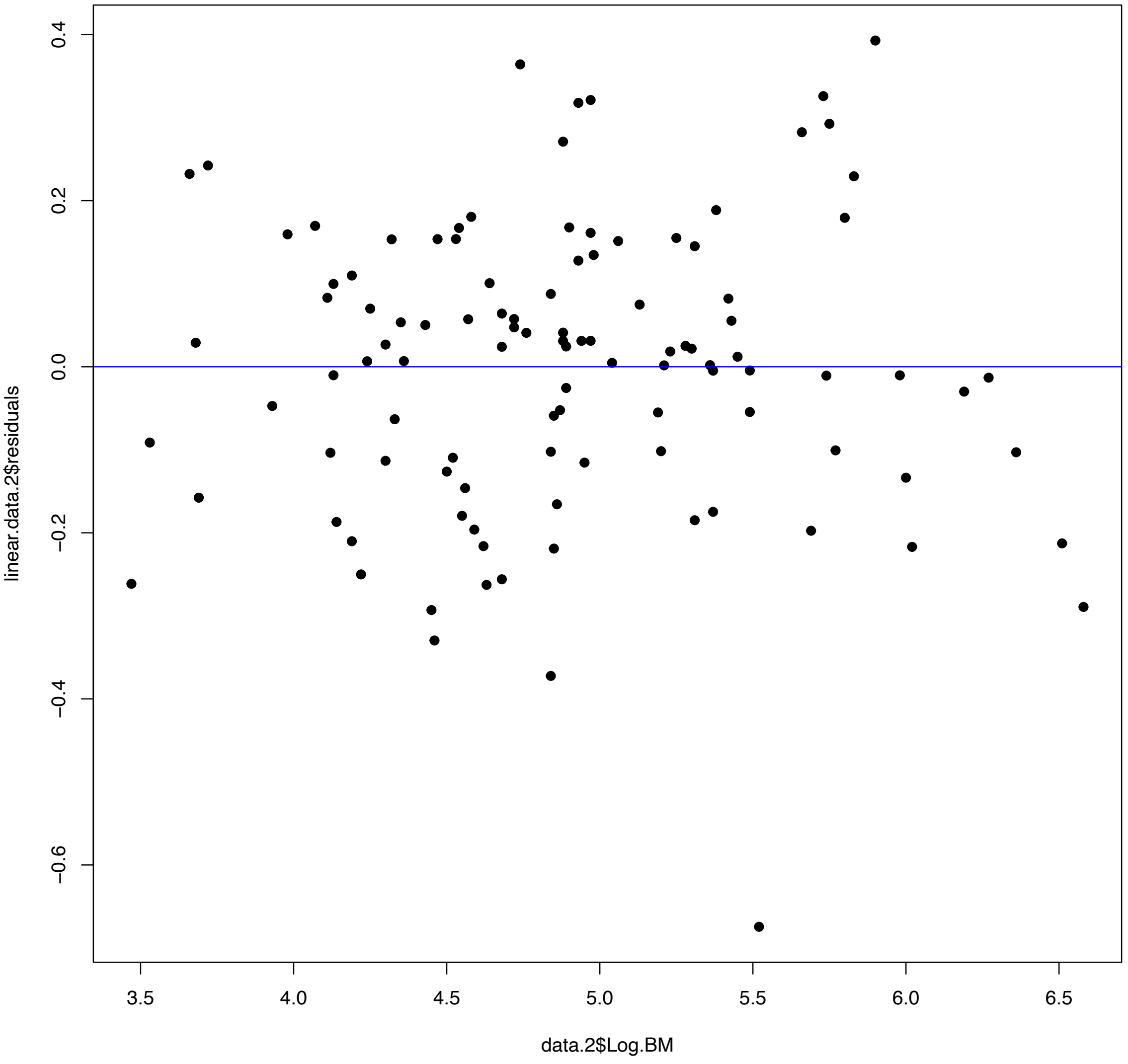

Supplementary figure 9. Distribution of the residuals of the log10 MBMGRB-M (adult-neonatal body mass, gr/day from birth, days) (y) against log10 adult body mass (x) regression of figure 9.

Scaling of age at sexual maturity in P. falconeri:

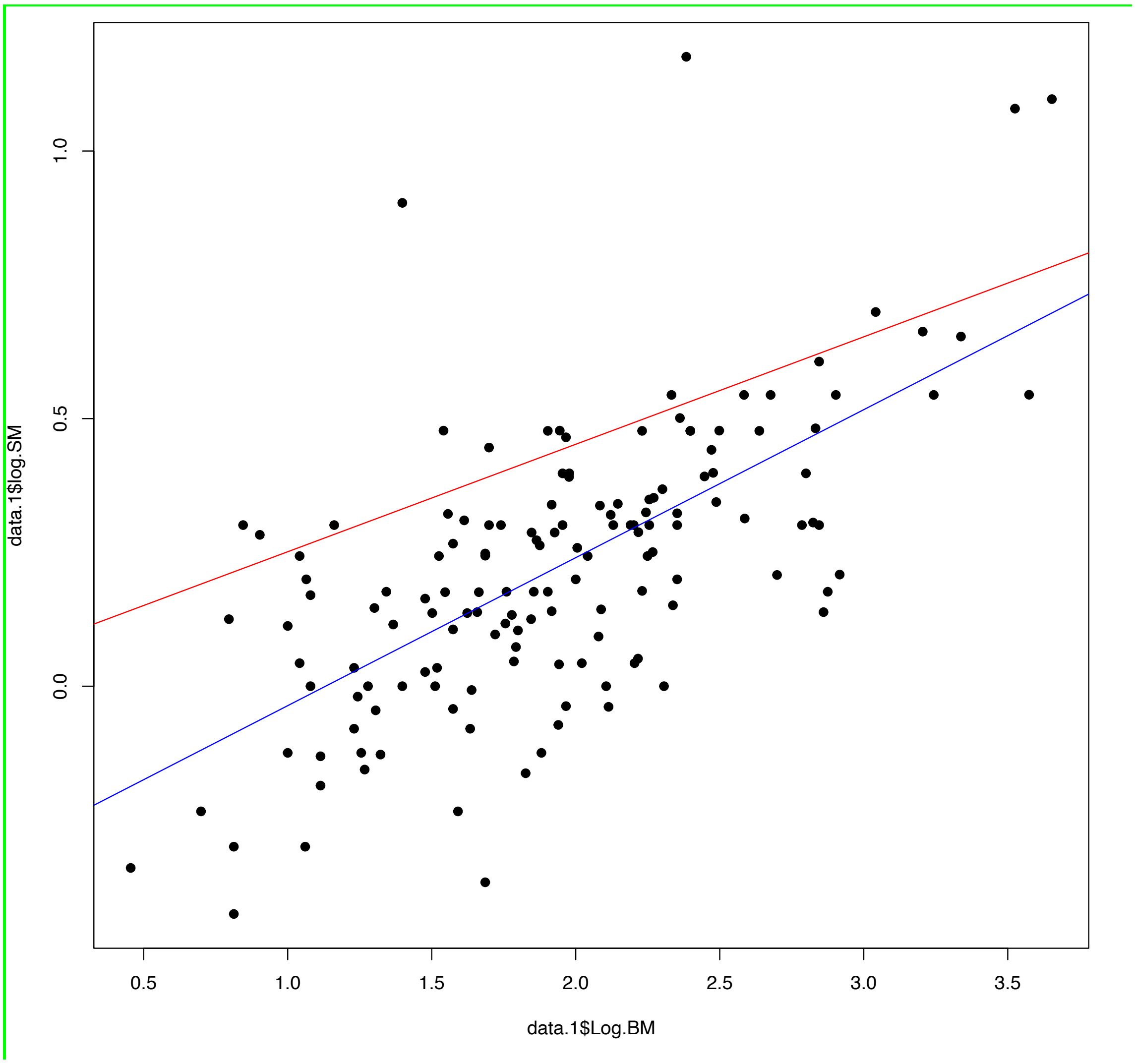

Supplementary figure 10. Scatterplot of age at sexual maturity (y; years) against adult body mass (x; kg) in log10. Results of PGLS: Y=a+bx, Intercept (a) 0.052366, Slope (b) 0.200756, Residual standard error: 0.02968 on 145 degrees of freedom, Multiple R-squared: 0.2165, Adjusted R-squared: 0.2111, F-statistic: 40.06 on 1 and 145 DF, p-value: 2.893e-09). The scaling of the age of sexual maturity in respect to adult body mass is based on a large sample of species of ungulate mammals (n=145) (Data from PanTheria, Jones et al., 2009). Blue line: Regression line without PGLS; Red line: Regression line with PGLS.

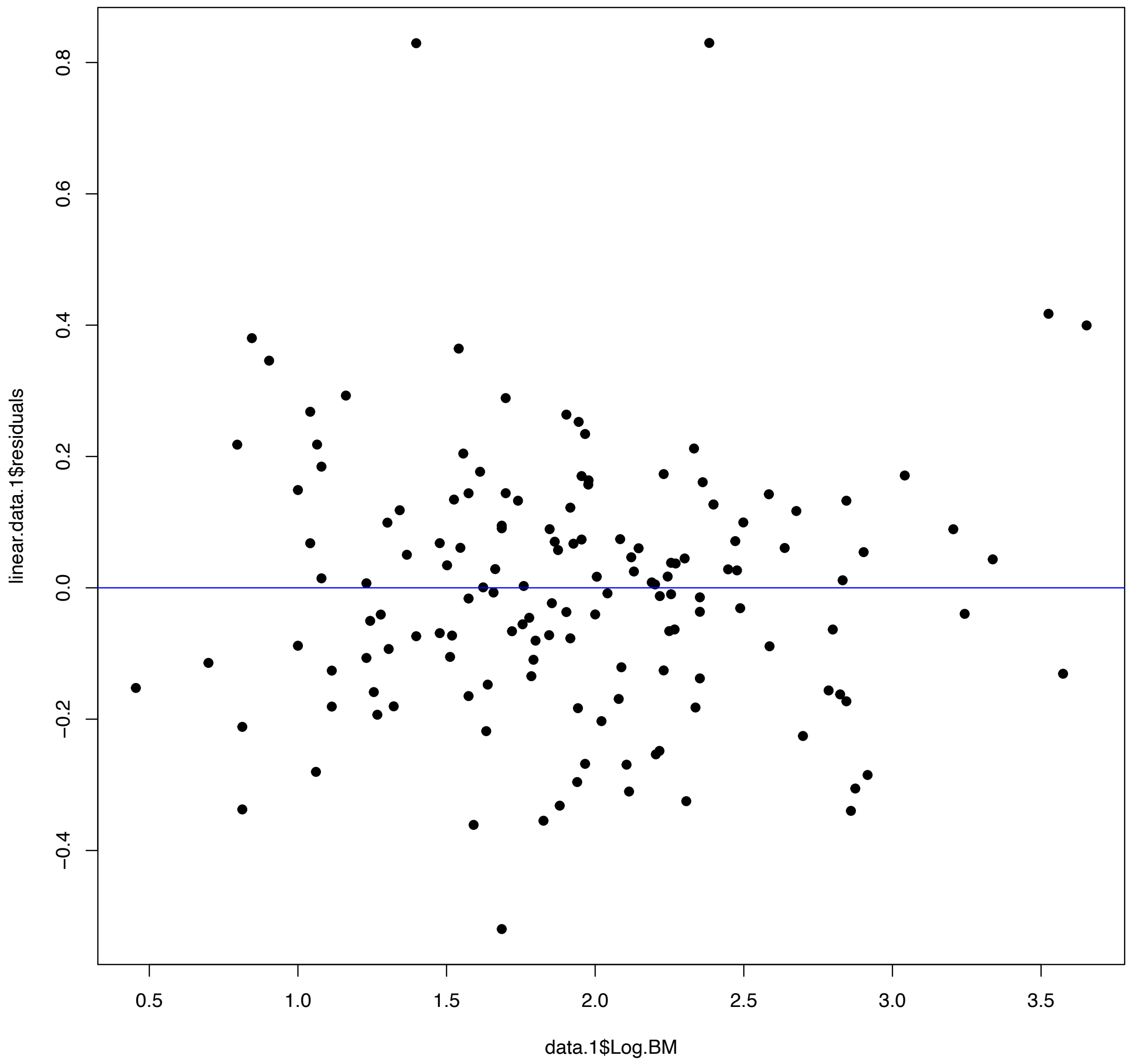

Supplementary figure 11. Distribution of the residuals of log 10 Age at Sexual maturity (y) against log10 adult body mass (x) regression of figure 11.

Scaling of longevity in P. falconeri.

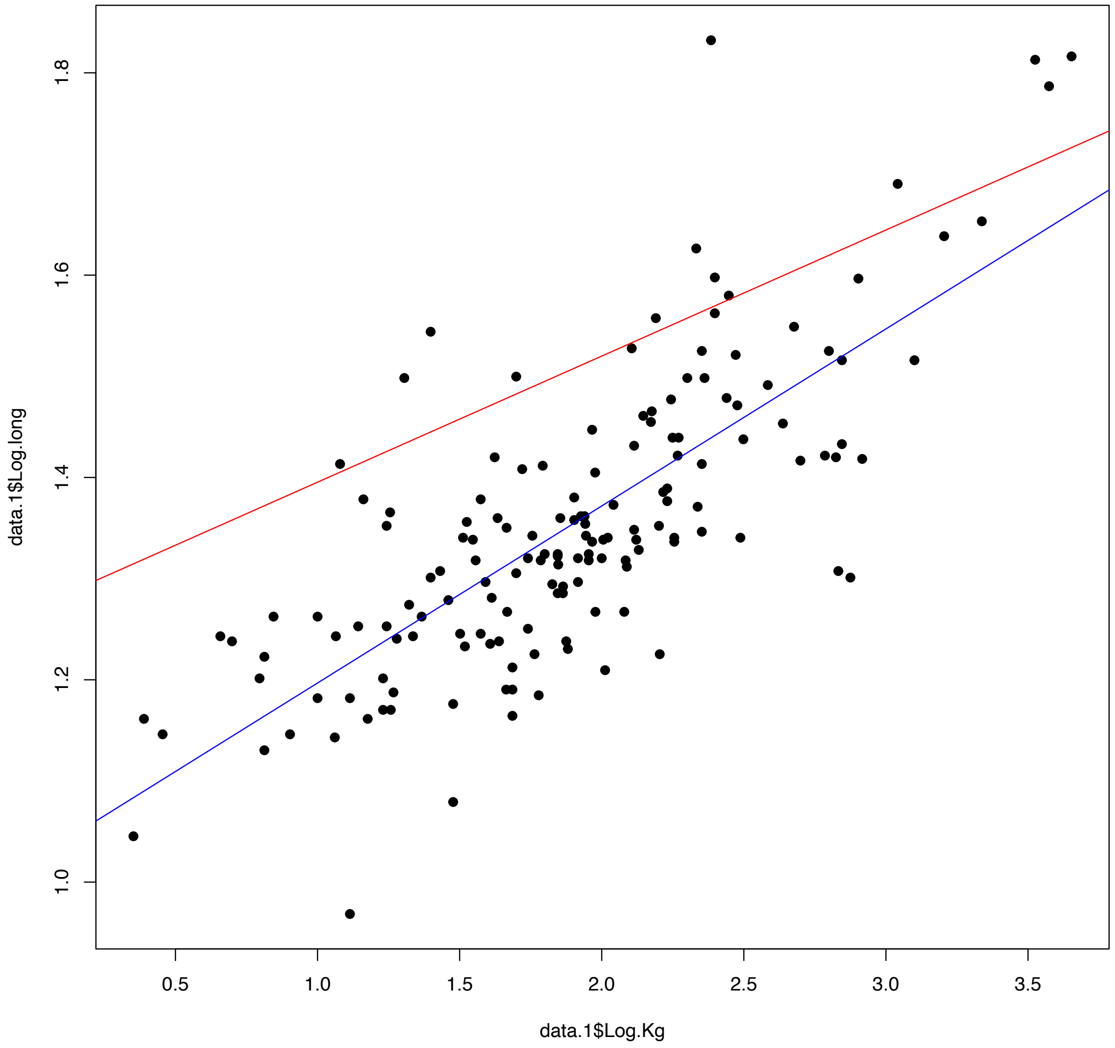

Supplementary figure 12. Scatterplot of longevity (y; years) against adult body mass (x; kg) in log10. Results of PGLS: Y=a+bx, Intercept (a) 1,270706, Slope (b) 0,124650, Residual 0.01456 on 155 degrees of freedom, Multiple R-squared: 0.3132, Adjusted R-squared: 0.3088, F-statistic: 70,69 on 1 and 155 DF, p-value: 2,546e-14. The scaling of longevity in respect to adult body mass is based on a large sample of species of ungulate mammals (n=156) (Data from PanTheria, Jones et al., 2009). Blue line: Regression line without PGLS; Red line: Regression line with PGLS.

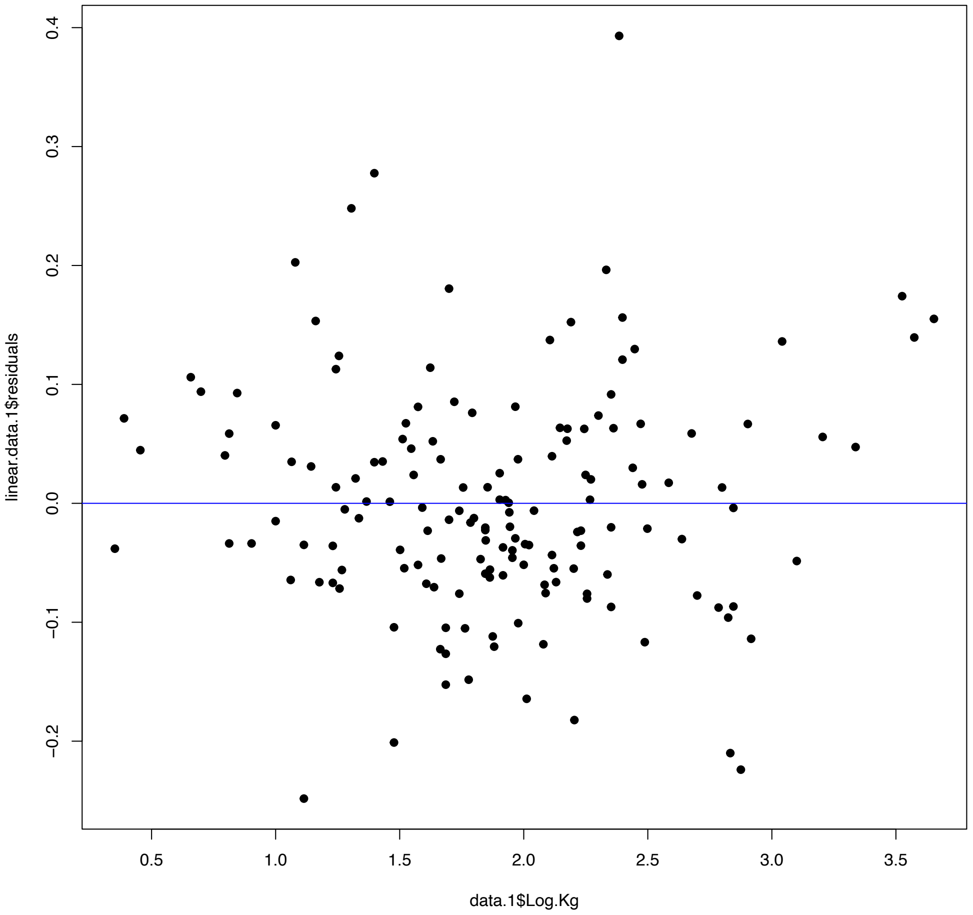

Supplementary figure 13. Distribution of the residuals of log 10 longevity (y) against log10 adult body mass (x) regression of figure 13.

	Growth rate (MBMGRB-M)	Sexual Maturity	Longevity
Phylogenetic signal lambda	0,9654	0,9006	0,9597
logL(lambda)	-53,9822	28,0823	147,0840
LR(lambda=0)	37,4599	79,5058	133,1880
P-value (based on LR test)	9,3309E-10	4,8081E-19	8,22E-26
Phylogenetic signal K	0,2139	0,3832	0,6862
P-value (based on 10000 randomiza	0,0001	0,0001	0,0001

Supplementary table 17. Values and statistics of the phylogenetic signal lambda and K for PGLS equations of growth rate (mean body mass growth rate from birth to maturity - MBMGRB-M), sexual maturity and longevity of extant ungulates and *P. falconeri* (Sicily) and *M. balearicus* (Mallorca).

9. The Spinagallo mortality profile.

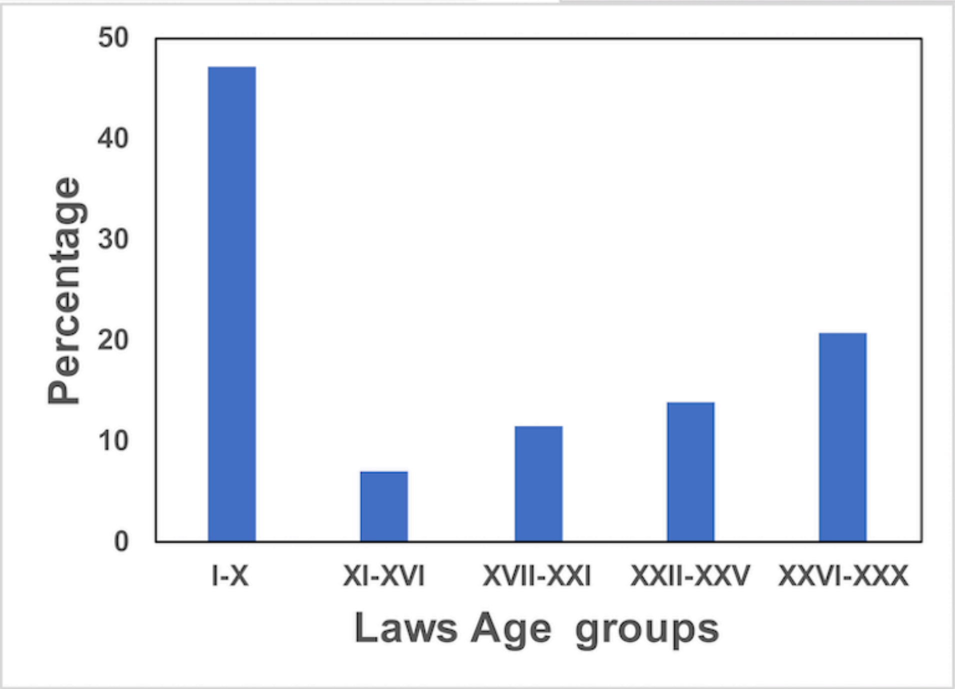

Supplementary figure 14. Mortality profile of the Spinagallo site based on the study of the lower dentition (n=87 individuals, right side). Age classes follow the Laws age groups (Laws, 1966). The mortality profile based on the ontogeny of the lower dentition provides a reliable mortality pattern of the Spinallo site. The profile shows a clear U-shape with a moderately high juvenile mortality (47%), a low prime adult-adult group and a high number of old-senescent individuals. This profile fits well with the "attritional" type, in which the very young and the old are best represented (Voorhies 1969). It is characteristic of fossil sites that represents important time periods and reflects mortality due to accidents, predation, endemic disease and other factors that ordinarily have their greatest impact on the very young and the old. This pattern contrast with the "catastrophic" model in which successive age classes contain progressively fewer individuals, reflecting the structure of a living population (Voorhies 1969). The "attritional" mortality profile of Spinagallo clearly discard the possibility that, as previously suggested (Ambrosetti, 1968, Raia, 2003), Spinagallo represents a population.

10. List of acronyms

ASM: age at sexual maturity
BM: body mass
BP: break point of Piecewise Regression
CAT: Catania; acronym of the specimens from the collection of the Museum of Paleontology of University of Catania.
CFT: Crown formation time
DSR: Tusk, dentine daily secretion rate
DER: Tusk, dentine extension rate
ESR: Molar daily enamel secretion rate
EER: Molar enamel extension rate
EFS: External fundamental system
FOI: tusk, first order increment
GR: growth rate
K: growth parameter of von Bertalanffy growth model
LAGs: lines of arrested growth
LH: Life-history
MBMGRB-M: mean body mass growth rate from birth to maturity
PGLS: Phylogenetic generalized least square regressions
PFT: cheek teeth, plate formation time
PGLS: Phylogenetic generalized least square regressions
SOI: tusk, second order increment
TDL: tibia diaphyseal length
TOI: tusk, third order increments (daily)
vBGM: Von Bertalanffy growth model

11. References

Ambrosetti P. 1968. The Pleistocene dwarf elephants of Spinagallo (Siracusa, South-eastern Sicily). *Geol. Romana*. **7**, 277-398.

Anderson JE, Hall-Martin A, Russell DA. 1985. Long bone circumference and weight in mammals, birds and dinosaurs. *J. Zool.* **207**, 53–61. https://doi.org/10.1111/j.1469-7998.1985.tb04915.x

Andreone F, Guarino FM. 2013. Giant and long-lived? Age structure in *Macroscincus coctei*, an extinct skink from Cape Verde. *Amphibia-Reptilia* **24**, 459-470. https://doi.org/10.1163/156853803322763927

Belluomini G, Bada JL. 1985. Isoleucine epimerization ages of the dwarf elephants of Sicily. *Geology* 13, 451–52. https://doi.org/10.1130/0091-7613(1985)13<451:IEAOTD>2.0.CO;2

Blomberg S.P., Garland T., Ives A.R. 2003. Testing for phylogenetic signal in comparative data: behavioral traits are more labile, *Evolution*, 57: 717-745.

Bromage T, Dirks W, Erdjument H, Huck M, Kulmer O, Öner R, SZandrock O, Schrenk F. 2002. A life history and climate change solution to the evolution and extinction of insular dwarfs: A cypriot experience. In: Waldren, W. H. & Ensenyat, J. A (Eds). World islands in prehistory: International insular investigations, Deia conference of prehistory, British Archeological Reports International Series p. 420-427.

Campione NE, Evans DC. 2012. A universal scaling relationship between body mass and proximal limb bone dimensions in quadrupedal terrestrial tetrapods. *BMC Biol.*, **10**, 60. https://doi.org/10.1186/1741-7007-10-60

Case TJ. 1978. On the evolution and adaptive significance of postnatal growth rates in terrestrial vertebrates. *Q. Rev. Biol.* **53** 243–282. DOI: 10.1086/410622

Christiansen P. 1999. Scaling of the limb long bones to body mass in terrestrial mammals. *J. Morph.* **239**, 167-90. https://doi.org/10.1002/(SICI)1097-4687(199902)239:2<167::AID-JMOR5>3.0.CO;2-8

Christiansen P. 2004. Body size in proboscideans, with notes on elephant metabolism. *Zool J Linn Soc.* **140**, 523–549. https://doi.org/10.1111/j.1096-3642.2004.00113.x

Coltorti G, Feraud A, Marzoli C, Peretto T, Ton-That P, Voinchet J-J, Bahain A, Minelli U, Thun H. 2005. New ^{40}Ar/^{39}Ar, stratigraphic and palaeoclimatic data on the Isernia La Pineta Lower Palaeolithic site, Molise, Italy. *Quat. Inter.* 131, 11–22. https://doi.org/10.1016/j.quaint.2004.07.004

Colyer F, Miles AE. 1957. Injury to and Rate of Growth of an Elephant Tusk. *Jour. Mam*., **38**, 243–247. https://doi.org/10.2307/1376316

Dale RHI. 2010. Birth statistics for African (*Loxodonta africana*) and Asian (Elephas maximus) elephants in human care: history and implications for elephant welfare. *Zoo Biology* **29**, 87–103. https://doi.org/10.1002/zoo.20234

Delitala M C, Fornaseri M, Nicoletti M. 1983. Datazioni Argon- Potassio sulla serie pleistocenica di Isernia la Pineta. In: Coltorti T. (ed.), Isernia La Pineta. Un accampamento piu` antico di 700.000 anni. Calderini, Bologna, p. 65–66.

Dirks W, Bromage TG, Agenbroad LD. 2011. The duration and rate of molar plate formation in *Palaeoloxodon cypriotes* and *Mammuthus columbi* from dental histology. *Quat. Int.* **225**, 79–85. https://doi.org/10.1016/j.quaint.2011.11.002

Eales NB. 1930. The Development of the Mandible in the Elephant. *Proc. Zool. Soc. London,* **101**, 115-127.

El Adli JJ. 2018. Reproductive Life Histories of Mammoths. *PhD thesis,* University of Michigan.

Eltringham SK. 1982. Elephants. Amherst: Poole & Dorset, Blandford Press.

Erickson GM, Rogers KC, Yerby SA. 2001. Dinosaurian growth patterns and rapid avian growth rates. *Nature* **412**, 429–433. https://doi.org/10.1038/35086558

Fisher DC, Fox D L. 2006. Five Years in the Life of an Aucilla River Mastodon. In: Webb, D. (Ed), First Floridians and Last Mastodons: The Page-Ladson Site in the Aucilla River, New York, Springer-Verlag p. 343–377.

Freeman EW, Whyte I, Brown JL. 2008. Reproductive evaluation of elephants culled in Krugger National Park, South Africa between 1975 and 1995. *Afr. J. Ecol*. **47**, 192-201. https://doi.org/10.1111/j.1365-2028.2008.00957.x

Griebeler EM, Werner J. 2018. Formal comment on: Myhrvold (2016) Dinosaur metabolism and the allometry of maximum growth rate. *Plos One*, **11** e0163205. https://doi.org/10.1371/journal.pone.0184756

Hanks J. 1972. Growth of the African elephant (*Loxodonta africana*). *E. Afr. Wildl. J*., **10**, 251- 272. https://doi.org/10.1111/j.1365-2028.1972.tb00870.x

Herridge VL. 2010. Dwarf elephants on Mediterranean islands: a natural experiment in parallel evolution. *PhD thesis*, University College London.

Herridge V, Nita D, Schwenninger J, Mangano G, Bonfiglio L, Lister A, Richards D. 2014 New chronology for Spinagallo Cave (Sicily): implications for the evolution of the insular dwarf elephant *Palaeoloxodon falconeri*. In: Abstract Book of the VI International Conference on Mammoths and Their Relatives S.A.S.G. 102, 70, Grevena- Siatista, Greece.

Jones KE, Bielby J, Cardillo M, Fritz SA, O'Dell J, David C, Orme L, Safi K, Sechrest W, Boakes EH, Carbone Ch, Connolly C, Cutts MJ, Foster JK, Grenyer R, Habib M, Plaster Samantha CA, Price SA, Rigby EA, Rist J, Teacher A, Bininda-Emonds ORP, Gittleman JL, Mace GM, Purvis A. 2009. PanTHERIA: a species-level database of life history, ecology, and geography of extant and recently extinct mammals. *Ecology*, **90**, 2648 (2009). https://doi.org/10.1890/08-1494.1

Jordana X, Köhler M, Madurell-Malapeira J, Rosso A, Sanfilippo R, Sciuto F. 2015. Dental histology of the sicilian dwarf elephant palaeoloxodon falconeri. In: Canoville A, Mitchell J, Stein K, Konietzko-Meier D, Teschner E, van Heteren H, Sander PM. (Eds). 3rd International Symposium on Paleohistology, University of Bonn.

Jordana X, Marín-Moratalla N, Moncunill-Solé B, Nacarino-Meneses C, Köhler M. 2016. Ontogenetic changes in the histological features of zonal bone tissue of ruminants: A quantitative approach. *Comp. Rend. Palevol.* **15**, 265–276. https://doi.org/10.1016/j.crpv.2015.03.008

Klevezal GA, Mina MV, Oreskhkin AV. 1998. Recording structures of mammals: determination of age and reconstruction of life history. First Edition. Taylor & Francis. https://doi.org/10.1201/9780203741146

Kozłowski J, Wiegert RG. 1987. Optimal age and size at maturity in annuals and perennials with determinate growth. *Evol Ecol* **1,** 231–244. https://doi.org/10.1007/BF02067553

Larramendi A. 2016 Shoulder height, body mass, and shape of proboscideans. *A. Palaeont, Pol.* **61**, 537–574. http://dx.doi.org/10.4202/app.00136.2014

Larramendi A, Palombo MR. 2015. Body size, biology and encephalization quotient of palaeoloxodon ex gr. *P. falconeri* from Spinagallo Cave (Hyblean plateau, Sicily). *Hystrix*, **26**, 102-109. https://doi.org/10.4404/hystrix-26.2-11478

Laws RM. 1966. Age criteria for the African elephant, *Loxodonta a. africana*. *E. Afr. Wildl.* J. **4**, 1–37. https://doi.org/10.1111/j.1365-2028.1966.tb00878.x

Laws RM, Parker IS. 1968. C. Recent studies on elephant populations in East Africa. *Symp. zool. Soc. Lond.* **21**, 319-359.

Lee AH, Werning S. 2008. Sexual maturity in growing dinosaurs does not fit reptilian growth models. *Proc. Natl. Acad. Sci. USA*, **105**, 582–587. https://doi.org/10.1073/pnas.0708903105.
Lemaître J-F, Berger V, Bonenfant Ch, Douhard M, Gamelon M Plard F, Gaillard J-M. 2015 Early-late life trade-offs and the evolution of ageing in the wild. Proc. R. Soc. B. B **282**, 20150209. http://doi.org/10.1098/rspb.2015.0209

Lister AM. 1999. Epiphyseal fusion and postcranial age determination in the woolly mammoth Mammuthus primigenius. In: Haynes, G., Klimowicz, J. and Reumer, J.WF. (Eds). Mammoths and the Mammoth Fauna: Studies of an Extinct Ecosystem. DEINSEA 6. pp 79-88.

Marín-Moratalla N, Jordana X, García-Martínez R, Köhler M. 2011. Tracing the evolution of fitness components in fossil bovids under different selective regimes. *C. R. Palevol* **10**, 469–478. https://doi.org/10.1016/j.crpv.2011.03.007

Mayne B, Berry O, Davies C, Farley J, Jarman S. 2019. A genomic predictor of lifespan in vertebrates. *Sci Rep* **9**, 17866. https://doi.org/10.1038/s41598-019-54447-w

Moss CJ. 1983. Oestrous behaviour and female choice in the African elephant. *Behaviour* **86**, 167-196. https://doi.org/10.1163/156853983X00354

Moss CJ. 2001. The demography of an African elephant (*Loxodonta africana*) population in Amboseli, Kenya. *J. Zool., Lond.* **255**, 145-156. https://doi.org/10.1017/S0952836901001212

Müller DWH, Zerbe P, Codron D, Clauss M, Hatt JM. 2011. A long life among ruminants: giraffids and other special cases. *Schweiz. Arch. Tierheilkd.* **153**, 515–519. https://doi.org/10.1024/0036-7281/a000263

Mumby HS, Chapman SN, Crawley JAH, Mar KU, Htut W, Soe, AT, Lummaa V. 2015. Distinguishing between determinate and indeterminate growth in a long-lived mammal. *BMC Evol. Biol.* **15**, 214. https://doi.org/10.1186/s12862-015-0487-x

Padian K, de Boef Miara M, Larsson HCE, Wilson, L, Bromage. T. 2013. Research applications and integration. Chapter 10 In: Padian, K. and E.-T. Lamm (eds.). Bone Histology of Fossil Tetrapods: Advancing Methods, Analysis, and Interpretation. University of California Press, Berkeley. p. 265-285.

Pagel, M. Inferring the historical patterns of biological evolution. 1999. *Nature* 401, 877–884. https://doi.org/10.1038/44766.

Palkovacs EP. 2003. Explaining adaptive shifts in body size on islands: a life history approach. *Oikos* **103**, 37–44. https://doi.org/10.1034/j.1600-0706.2003.12502.x

Palombo MR. 2007. How can endemic proboscideans help us understand the ‘‘island rule’’? A case study of Mediterranean islands. *Quat. Int* **169–170**, 105-125. https://doi.org/10.1016/j.quaint.2006.11.002

Palombo MR, Giovinazzo C. 2005. *Elephas falconeri* from Spinagallo Cave (South-Eastern Sicily, Hyblean Plateau, Siracusa): a preliminary report on brain to body weight comparison. In Alcover JA, Bover P. Eds. Proceedings of the International Symposium "Insular Vertebrate Evolution: the Palaeontological Approach”. *Monografies de la Societat d'Història Natural de les Balears*, 12: 255-264.

Portugal SJ, White CR, Frappell PB, Green JA, Butler PJ. 2019. Impacts of “supermoon” events on the physiology of a wild bird. *Ecol. and Evol.* **9**, 7974–7984. https://doi.org/10.1002/ece3.5311

Quince C, Abrams PA, Shuter BJ, Lester NP. 2008. Biphasic growth in fish I: Theoretical foundations. *Jour. Th. Biology*, **254**, 197-206. https://doi.org/10.1016/j.jtbi.2008.05.029

Quince C, Shuter BJ, Abrams PA. Lester NP. 2008. Biphasic growth in fish II: Empirical assessment. *Jour. Th. Biololigy*. **254**, 207– 214. https://doi.org/10.1016/j.jtbi.2008.05.030

Raia P, Barbera C, Conte M. 2003. The fast life of a dwarfed giant. *Evol. Ecol.*, **17**, 293-312. https://doi.org/10.1023/A:1025577414005

Revell, L. J. 2012. phytools: An R package for phylogenetic comparative biology (and other things). *Methods Ecol. Evol.* 3 217-223. doi:10.1111/j.2041-210X.2011.00169.x

R Core Team 2019. R: A Language and Environment for Statistical Computing. Vienna: R Foundation for Statistical Computing. Available online at: http:// www.R-project.org/

Richards D, Herridge V, Nita D, Schwenninger J, Lister A, Mangano G, Bonfiglio L. 2019. Constraining the chronology of dwarf elephant evolution using Quaternary deposits in coastal caves of the Mediterranean. In: 20th Congress of the International Union for Quaternary Research; Dublin, Ireland. Abstract P-2810.

Romano M, Manucci F, Palombo MR. 2019. The smallest of the largest: new volumetric body mass estimate and in-vivo restoration of the dwarf elephant *Palaeoloxodon* ex gr. *P. falconeri* from Spinagallo Cave (Sicily). *Hist. Biol.* https://doi.org/10.1080/08912963.2019.1617289

Roth VL. 1990. Insular dwarf elephants: a case study in body mass estimation and ecological inference. In: Damuth J, MacFadden BJ, Eds. Body size in Mammalian Paleobiology: Estimation and Biological Implications. Cambridge University Press. P. 151–180.

Sikes SK. 1966. The African elephant, *Loxodonta africana* field method for the estimation of age. *J. Zool. Lond.*, **150**, 279-295. https://doi.org/10.1111/j.1469-7998.1966.tb03009.x

Smuts GL. 1975. Reproduction and population characteristics of elephants in Kruger National Park. *J. Sth. Afr. Wildl. Mgmt. Assoc*. **5**, 1–10.

Stearns SC. 1999. The evolution of life histories. Oxford University Press.

Stansfield FJ. 2015. A novel objective method of estimating the age of mandibles from African elephants (*Loxodonta africana africana*). *PLOS One* **10**, e0124980. https://doi.org/10.1371/journal.pone.0124980

Tacutu R, Thornton D, Johnson E, Budovsky A, Barardo D, Craig T, Diana E, Lehmann G, Toren D, Wang J, Fraifeld VE, Magalhães JP. 2018. Human Ageing Genomic Resources: new and updated databases. *Nucleic Acids Res.* **46**,1083-1090. https://doi.org/10.1093/nar/gkx1042

Uno KT, Quadeb J, Fisherc DC, Wittemyere G, Douglas-Hamilton, I, Andanjeh S, Omondih P, Litoroh M, Cerlinga TE. 2013. Bomb-Curve Radiocarbon Measurement of Recent Biologic Tissues and Applications to Wildlife Forensics and Stable Isotope (Paleo) Ecology. *Proc. Natl. Acad. Sci. U.S.A* **110**, 11736–11741. https://doi.org/10.1073/pnas.1302226110

Werner J, Griebeler EM. 2014. Allometries of maximum growth rate versus body mass at maximum growth indicate that non-avian dinosaurs had growth rates typical of fast growing ectothermic sauropsids. *PLoS One.* **9** e88834. https://doi.org/10.1371/journal.pone.0088834

Western D. 1979. Size, life history and ecology in mammals. *Afr. J. Ecol*., **17**, 185-204. https://doi.org/10.1111/j.1365-2028.1979.tb00256.x

Whyte I. 1996. Collecting Data from Dead Elephants. In: Kangwana K, (Ed.) Studying Elephants. Nairobi-Washington: African Wildlife Foundation. 171-177.

Whyte IJ, Hall-Martin A. 2018. Growth characteristics of tusks of elephants in Kruger National Park. *Pachyderm*, **59**, 31-40. https://pachydermjournal.org/index.php/pachyderm/article/view/78
